# Supplementary material for: Watching videos of a drawing hand improves students’ understanding of the normal probability distribution
Source: Mem Cognit. 2024 Feb 20;53(1):262–81. doi: 10.3758/s13421-024-01526-7 (PMC11779760; doi:10.3758/s13421-024-01526-7)
Supplement: Supplementary file 1 — Supplementary file1 (DOCX 9279 KB) [file 13421_2024_1526_MOESM1_ESM.docx]

**Watching Videos of a Drawing Hand Improves**

**Students’ Understanding of the Normal Probability Distribution**

**Abstract**

Understanding normal probability distributions is a crucial objective in mathematics and statistics education. Drawing upon cognitive psychology research, this study explores the use of drawings and visualizations as effective scaffolds to enhance students' comprehension. Although much research has documented the helpfulness of drawing as a research tool to reveal students’ knowledge states, its direct utility in advancing higher-order cognitive processes remains understudied. In Study 1, qualitative methods were utilized to identify common misunderstandings among students regarding canonical depictions of the normal probability distribution. Building on these insights, Study 2 experimentally compared three instructional videos (static slides, dynamic drawing, and dynamic drawings done by a visible hand). The hand drawing video led to better learning than the other versions. Study 3 examined whether the benefits from observing a hand drawing could be reproduced by a dynamic cursor moving around otherwise static slides (without the presence of a hand). Results showed no significant learning difference between observing a hand drawing and a moving cursor, both outperforming a control. This research links the cognitive process of drawing with its educational role and provides insights into its potential to enhance memory, cognition, and inform instructional methods.

*Keywords:* drawing, dynamic representation, embodied cognition, multimedia learning, statistics instruction

**Watching Videos of a Drawing Hand Improves**

**Students’ Understanding of the Normal Probability Distribution**

In college-level introductory statistics classes, understanding and using the normal probability distribution is an important learning outcome interrelated with many other skills and concepts (Ainsworth, 2008; Batanero et al., 2004; Cohen & Chechile, 1997). Teachers employ a number of representations, such as static and dynamic visualizations and drawings, to help students learn about the normal distribution. However, we lack an understanding of how and why students struggle to understand the normal probability distribution and what instructional practices can be useful to alleviate the struggle.

The studies reported have two objectives. First, we explored what students do and do not understand about normal distributions and the visualizations teachers use to represent them (Study 1). To this end, we surveyed a small sample of students nearing the end of a college-level introductory statistics class that had been explicitly taught about normal distributions. Qualitative work diving into what students do not understand about normal probability distributions is a crucial foundation for experiments that explore the effect of different instructional tools because the qualitative work helps identify concepts to teach and pain points to address, which may reveal student misunderstandings that teachers have assumed students to all understand (Airey & Linder, 2009; Rau, 2017; Uttal & O’Doherty, 2008). For example, when teachers shade in some area under a normal curve to represent a corresponding probability, do students understand that the normal curve is a probability distribution and thus that the total area under the curve equals 1? Do students understand the relationship between a continuous probability distribution and a discrete histogram, or why it might be helpful to overlay one on top of the other? In essence, teachers may be asking students to make sense of novel concepts via visualizations they do not understand (Airey & Linder, 2009).

To foreshadow our results, we found evidence that many students sampled in the current study, even after instruction, had a relatively weak understanding of what normal distributions were, with many misconceptions and misinterpretations of the canonical visualization. This finding raises troubling questions about what teachers should expect students to learn from visualizations of the normal distribution, a common foundational visualization that instructors rely on to communicate new ideas about statistics (Chance et al., 2004).

Second, based on these findings, we attempted to teach students how to interpret commonly used representations of normal probability distributions via a targeted intervention (Study 2 and Study 3). We created our instructional materials in the form of brief videos, and grounded their design in theories and findings from the cognitive sciences and educational psychology. We drew on three research literatures: static vs. dynamic visualization, embodied cognition, and drawing. We endeavored to make the interventions brief so they could be more easily added as supplementary materials to an ongoing statistics class. Although the video interventions were brief, our goal was to produce lasting learning that may enable students to better interpret future explanations of statistical concepts couched in terms of the normal curve. Across the two studies, we developed several interventions and tested their effects via both immediate and delayed posttests. Below, we discuss the rationale behind the design of our interventions.

**Literature Review**

**Static and Dynamic Visualizations**

Visualizations can help people learn new concepts (Shepard, [1967](https://link.springer.com/chapter/10.1007/978-3-030-20969-8_5#ref-CR98)), as predicted by many theories in psychology (e.g., visual argument hypothesis; Vekiri, 2002; dual coding theory; Clark & Paivio, [1991](https://link.springer.com/chapter/10.1007/978-3-030-20969-8_5#ref-CR30)). Visualizations can be either static, such as pictures, or dynamic, as such animations. Static visualizations, such as diagrams, flowcharts, graphs, maps, and schematic illustrations of objects or processes are ubiquitous in Science, Technology, Engineering, and Mathematics (STEM) education, both in textbooks and formal instruction (Gilbert, 2005; Gilbert et al., 2007). Visualizations benefit learning across topics/domains, assessment types, and visualization formats (Bauer & Johnson-Laird, 1993; Bartram et al., 1980; Carney & Levin, 2002; Holliday, 1977; Scanlan, 1989; Rau, 2017).

Dynamic visualizations, defined in their prototypical form, are animations of some visible phenomenon, with visual representations appearing gradually on the screen (Castro-Alonso et al., 2015; Hegarty, 2004). Dynamic visualizations can benefit learning in STEM domains by showing typically unseen processes and abstract concepts in a concrete way that unfolds over time (e.g., chemical reactions on the molecular level; Zhang & Linn, 2011). Dynamic visualizations have been widely used by instructors and researchers for decades as a way of teaching complex concepts and systems (Ayres & Paas, [2007](https://link.springer.com/article/10.1007/s10648-008-9094-3#ref-CR3); Mayer, [2005](https://link.springer.com/article/10.1007/s10648-008-9094-3#ref-CR39); Van Gog et al., 2009).

Dynamic visualizations might be superior to traditional static visualizations for three reasons. First, dynamic visualizations can explicitly represent not just the end result of processes but, rather, the processes themselves (Castro-Alonso et al., 2014; Chandler, 2004; Hegarty, 2004; Mayer & Moreno, 2002). Second, research has shown that dynamic visualizations may reduce cognitive load by “distributing” new information across time (for a review, see Ainsworth, 2008) and, relatedly, better match the computational demands of learning (Tversky et al., 2002; Wong et al., 2009). Third, dynamic visualizations may be more engaging or motivating than static ones (Rieber, 1991). A meta-analysis of 47 independent comparisons of static versus dynamic representations found that dynamic representations promote conceptual development and inferences in science domains to a higher degree than static visualizations (McElhaney et al., 2015). However, in cases where dynamic visualizations do show learning benefits, it is unclear whether the observed benefits are simply a result of longer time spent interacting with the dynamic visualizations.

Despite some evidence suggesting that the benefit of dynamic visualizations may lie in their ability to alleviate cognitive load (e.g., Ainsworth, 2006), others argue that such visualizations may, in fact, tax working memory because of their inherently transient nature (Ayres et al., 2009; Chandler, 2004; Hegarty, 2004; Höffler & Leutner, 2007; Lowe, 2004). As a video or animation changes over time, the earlier parts are gone and can no longer be accessed by learners (Castro-Alonso et al., 2014). Even when learners can replay the video, they still need to mentally rehearse the information to keep it activated once the video has advanced, which taxes working memory (Sweller et al., 2011). A potential way to counteract this effect might be to connect dynamic visualizations, through bodily actions, to the learner’s own physical experience in the world (de Koning & Tabbers, 2011). In other words, "embodying" visualizations could aid students in reaping the benefits of dynamic visualizations.

**Embodied cognition**

An embodied cognition framework assumes that people’s physical movements can shape cognition and learning (Da Rold, 2018; Tran et al., 2017). A growing body of work demonstrates that embodiment can even support the learning of highly abstract concepts and higher-order skills such as problem-solving and reading comprehension (Glenberg et al., 2008; Thomas & Lleras, 2009; Zhang et al., 2021). Importantly, the facilitative effect of bodily movements during learning has been demonstrated even when the learners themselves are not the source of the bodily action but are simply observing others’ bodily movements in a video (Da Rold, 2018; de Koning & Tabbers, 2013; Glenberg et al., 2008, 2011; Son et al., 2018; Thomas & Lleras, 2009; Tran et al., 2017).

In the specific context of multimedia learning, students’ learning is enhanced by including an instructors’ gestures in instructional videos (Rueckert et al., [2017](https://onlinelibrary.wiley.com/doi/full/10.1111/cogs.12940?casa_token=5ptumI1Ly-sAAAAA%3Aoc8K_wh3qjBuiD1lYRQYphzpvem0QkEqpisAAB345ICtcurKCviNbbiCVqYgHGn-5y6-ZeQxFX3X2nQ#cogs12940-bib-0027); Son et al., [2018](https://onlinelibrary.wiley.com/doi/full/10.1111/cogs.12940?casa_token=5ptumI1Ly-sAAAAA%3Aoc8K_wh3qjBuiD1lYRQYphzpvem0QkEqpisAAB345ICtcurKCviNbbiCVqYgHGn-5y6-ZeQxFX3X2nQ#cogs12940-bib-0030)). For instance, in one study, students who watched an instructional video with a human-like pedagogical agent who performed gestures and displayed facial expressions learned more than students who watched a video with a static pedagogical agent (Mayer & DaPra, 2012). In another study, students who learned about electrical circuit analysis by attending to a pedagogical agent’s deictic movements learned more than students who were similarly guided by an animated arrow or who were not provided any elements designed to guide their attention (Moreno et al., 2001, 2010).

Mayer (2014) has referred to the idea that the instructor’s bodily movement can enhance instruction and learning as the *embodiment principle* in multimedia learning. According to this principle, embodiment cues are hypothesized to be most beneficial when they serve to guide cognitive processes that specifically support learning (e.g., helping students allocate attention to or process important information) (Mayer, 2014). But the embodiment principle encompasses other mechanisms as well. For example, embodiment cues may communicate to students that the instructor cares about them, which in turn creates a sense of social partnership between the students and the instructor (Mayer, 2014; Moreno et al., 2010).

To unpack the potential cognitive mechanisms underlying the effects of embodiment, we consider the potential benefits that come from recruiting multimodal systems during learning (Garcia et al., 2020). The presence of embodiment during learning may recruit additional sensorimotor systems for processing stimuli, which might not otherwise be engaged. This sensorimotor engagement could create more embodied representations of the content (e.g., spatial, temporal) to augment other less embodied representations (e.g., linguistic, notational, or a-modal / abstract). These embodied representations may be more robust and interconnected with other representations, or simply easier to retrieve later on.

Research has provided preliminary evidence that learners’ previously established visual representations mediated the effect of embodiment on learning (Author et al., under review). Specifically, learners who watched videos with hands-on demonstrations referred back more to those visual representations during a posttest than learners who did not watch videos with embodiment, and learners who referred back more to visual representations performed better on the posttest. Embodiment may have led to the encoding of richer visual representations, more distributed across cognitive systems, thereby decreasing the chances of forgetting the information or by making it easier to retrieve that information. Embodiment may have also eased the cognitive load from the transient nature of dynamic multimedia presentations, by providing additional sensorimotor pathways for encoding and processing temporal information alongside the already active pathway of language processing (Sepp et al., 2019).

**Drawing**

An interesting case to explore the combination of dynamic visualizations and embodied cognition is instructional drawings presented to students. Learners might benefit directly from performing generative drawings (Schemeck et al., 2014) but they also may benefit from watching others draw (e.g., on blackboards), as commonly happens in instructional contexts (Quillin & Thomas, 2015). Indeed, students learned more from a biology lecture accompanied by dynamic drawing (as in a Khan Academy video) than from a lecture that only featured the final product of the drawing (Fiorella et al., 2019). Similarly, students who watched an instructor drawing and explaining the doppler effect learned more than students who viewed only the final product of the drawings while listening to the same verbal explanation (Fiorella & Mayer, 2016).

Both watching the instructor’s full body and watching only the instructor’s hand had positive effects on students’ learning of the doppler effect when compared to a control group that observed completed, already-drawn diagrams. In contrast, watching a dynamic drawing of the same diagrams without a visible hand did not improve learning of the doppler effect when compared to the control group (Fiorella & Mayer, 2016). These findings suggest that watching the hand generate the drawing⎯an embodied component⎯may be a necessary component of drawings as an effective teaching device.

From these few studies, we can see at least two features of drawings that seem to have a beneficial effect on learning. First, the dynamic unfolding of the drawing over time is better than a static drawing. Dynamic unfolding can slow down and guide the comprehension process, allowing learners to observe individual components, notice subtleties, and process and integrate components over time leading to a wholly different processing experience (Fiorella & Zhang, 2018; Quillin & Thomas, 2015). It also may support joint attention because the instructor's drawing activity temporally matches the parts of the diagram/figure that students are supposed to be attending to (Suthers, 2014).

Second, embodied dynamic drawings, generated by a visible hand, are better than a *static* drawing without a hand. Fiorella and Mayer (2016) found that watching drawing generated by a visible hand was significantly better than viewing a static drawing; but watching drawing without a visible hand was not. What we do not know is whether a dynamic drawing with a visible hand is better than a dynamic drawing without a hand. Further, given that the research on drawing is not well connected with research on visualizations more generally, we do not know how dynamic drawings (embodied or not) compare to non-drawn static or dynamic visualizations such as those generated by software (such as R) and used in statistics courses.

**Current Studies**

In the current research, we tested whether watching a hand drawing, which is both dynamic and embodied, can improve learners’ understanding of the normal probability distribution. First, in order to identify where learners’ understanding needs improving, we leveraged the benefits of qualitative analysis to explore what students knew about drawings of the normal distribution (Study 1). Then, we designed two experiments to investigate (i) whether observing a hand drawing could improve students’ learning over and above what they might get from static slides (Study 2); and (ii) whether the learning benefits of drawing could be achieved by an enhanced “dynamic” version of the static slides where a moving cursor directs the student's attention (Study 3). In both Study 2 and 3, we were interested in cognitive and metacognitive effects of the intervention. The outcome variables of interest were students’ performance on assessment questions as well as the accuracy of their judgments of learning. These research questions are especially important because many instructional videos on the internet consists of narrated static slide decks, which only sometimes include a small “talking head”; and in previous research, we found that hands were visible at all in only 32% of a sample of YouTube instructional videos focused on the concept of standard deviation (Son et al., 2018).

# Study 1

## Method

### Participants

Participants were 39 undergraduate students at a large public research institution, taking a 10-week introductory statistics course. Due to the COVID-19 pandemic, the entire course was taught remotely (online). Because COVID also limited ways of recruitment, students volunteered to participate in the study for extra credit toward their final course grade and did not get any other form of compensation. All students were presented with the opportunity and the extra credit was worth 0.5% of students’ final course grade. The amount of extra credit was not high so that students would not risk discomfort to participate. The survey activity also provided potential educational value because the content (the normal distribution) was part of the course's learning outcomes. These characteristics of recruitment meet the justice and beneficence criteria of using course extra credit to recruit participations (Fuad & Jones, 2012). The institutional review board approved the study. Consent was obtained from participants online.

### Design & Procedure

Students were emailed an invitation to participate in the study near the end of the 10-week course. By that point, students had already been taught the basics of the normal distribution. Students who wished to participate clicked a link to complete a Qualtrics survey (Qualtrics, Provo, UT). As they worked through the survey, students could not go back to revise their answers to previous questions. Upon completion, participants were asked to rate the difficulty of the survey as a whole, on a scale from 0 to 10 (0 = not hard at all), so that we could understand whether participants perceived the survey to be challenging or not. The time to complete the survey ranged from 30 minutes to one hour.

### Materials

The survey consisted of 15 questions about probability (Appendix A), including basic characteristics of a normal curve, as well as more advanced topics such as sampling distributions, *p*-values, and related statistical topics. The survey was used to assess students’ understanding of a range of topics covered in the course. Only the four open-response questions specifically about interpreting drawings of the normal probability distribution were used for the current study (see Results). For example, we asked what is the total area under a curve drawn to represent the normal probability distribution. We also asked questions to test whether students understood the symmetric property of a normal probability distribution and could use it to estimate probabilities.

**Coding**

The first author, who has taught the class many times using the same curriculum as used by the students, graded each response to the four questions as either correct or incorrect based on a predetermined coding rubric co-developed with the instructor of the class. Then, the same experimenter read participants’ incorrect answers to find common misconceptions. After identifying the misconceptions, all incorrect responses were categorized as to whether or not they provided evidence of each misconception. Although there are disadvantages to using only one coder, the advantage in this case is that the coder was highly experienced in interpreting students’ responses in the context of this particular curriculum. Findings that result from Study 1 are tested more objectively in Study 2.

## Results

Below, we report on findings from the four questions related to normal probability distributions.

**Table 1**

*Summary of Study 1 Results*

| Question | Correctness (out of 35) | Type of errors | | |
| --- | --- | --- | --- | --- |
| What is the probability represented under the entire curve? | 64.1%(25) | Calculating the probability requires knowing the numbers on the *x-*axis. | Calculating the probability requires knowing the z-score | |
| What is the probability represented by a shaded region (everything above the mean)? | 100% (39) | NA | | |
| Do two normal distributions of different widths differ in their total area? | 35.9% (14) | The probability will change with the shape of the distribution | Only focusing on the shape of the distribution without reasoning about probability | Concluding the probability will not change but cannot explain why |
| What is the probability of falling above a certain value?( requires knowledge of the symmetric property and of the entire area under the curve) | 35.9%(14) | Do not know how to calculate the probability given the graph | | |

On average, students rated the difficulty of the questions as 6.44 on a 0 to 10 scale (SD = 1.89), which suggests that students perceived the questions to be somewhat challenging.

In the first question, students were presented with a curve described as a normal probability distribution, with the entire area under the curve shaded (Figure 1). They were asked whether it was possible to estimate the probability represented by the shaded region and elaborate on their answer in an open response.

**Figure 1**

*A Normal Curve in which All Area under the Curve is Shaded, from Question 1*


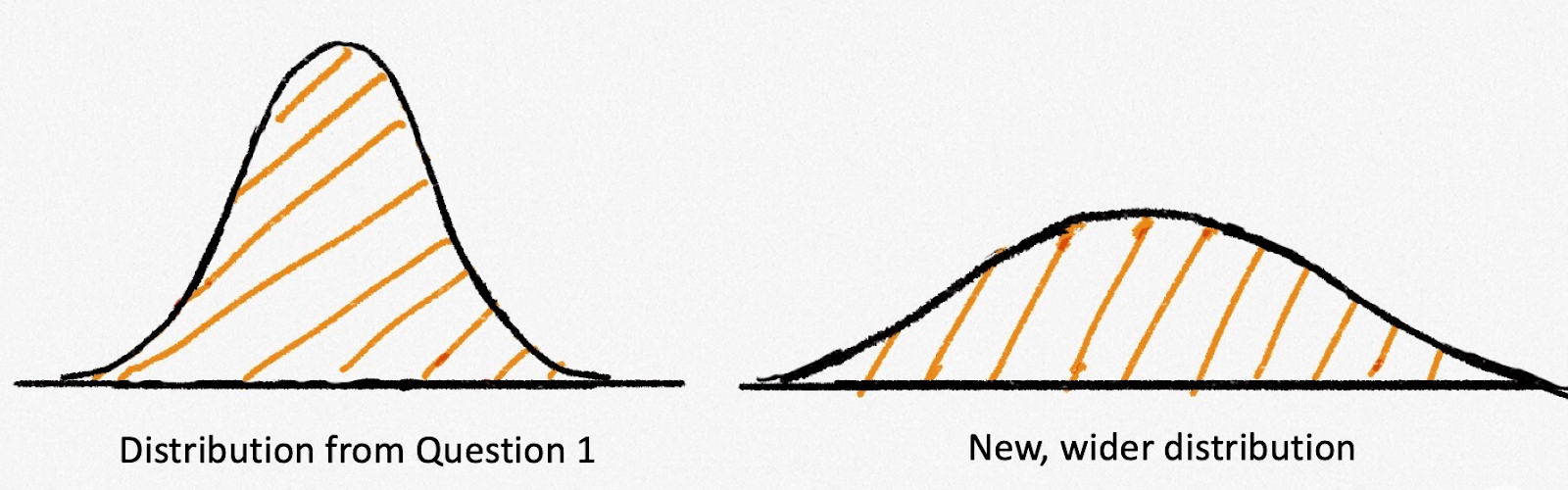


Out of 39 students, about one-third (14 students) did not know that the total probability under the curve is equal to 1. Out of these, 10 students (25.6%) said that the probability could not be estimated because there were no numbers on the *x-*axis. One student wrote:

*“Since this is a bell shaped curve there is an equal amount of values before and after the median in the center of the curve. Given that there are no values on the graph I wouldn't be able to estimate a specific number to represent the shaded region.”*

Other than these 10 students, the remaining four students who answered the question incorrectly either said that they could estimate the probability under the curve but did not give a specific value, or gave a wrong explanation. One of these students wrote: “*Yes you can [estimate the probability], why, because the area under the curve, my estimate would be depending on what it is asking and deal with Z scores.*” Many students who said that the probability could not be estimated similarly thought, erroneously, that computing this probability required a *Z*-score.

The second question presented students with another normal curve, this time with a vertical line marking its center. The area to the right of the center line was shaded (Figure 2), and students were again asked whether they could estimate the probability represented by the shaded area. In contrast to question 1, all 39 students answered that they could, though two of them did not provide specific values. Most students—including those who previously had said they could not estimate the probability when the total area under the curve was shaded—seemed to understand that half of the area under the curve represented a probability of 0.5. Thus, the same students who previously thought they would need numerical values on the *x*-axis in order to estimate total probability under the curve had no problem generating a probability when only half the region under the curve was shaded. One of these students said, “*The probability is 50% since half of the data points fall under the shaded region*.”

**Figure 2**

*Normal Curve with the Upper Half Shaded, from Question 2*


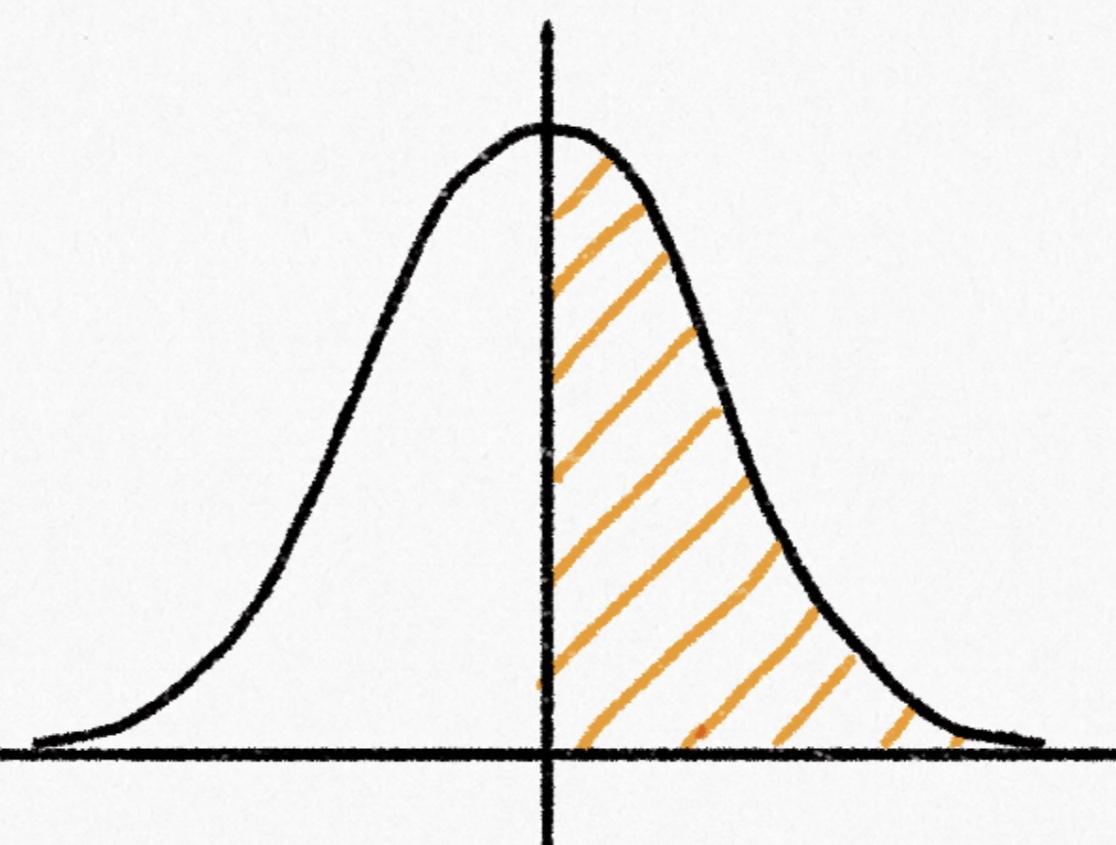


The third question again presented students with the curve from Question 1, but this time, paired it with a wider normal curve. The total area under each curve was shaded (Figure 3). We asked students in a multiple-choice question: “*If we draw a normal distribution that is wider than the one in Question 1 (as shown below), how would the probability represented by the shaded part under the distribution change?*” and then asked students to explain their answers. Only 14 students (35.9%) answered correctly that the probability would not change and provided a reasonable explanation of their answer. For example, one student answered: “*The probability is still 100% because the whole distribution is shaded in*"; another said: “*It would not change at all. The area under the curve still represents the entire probability*.”

**Figure 3**

*Two Normal Curves Differing in Width, from Question 3*


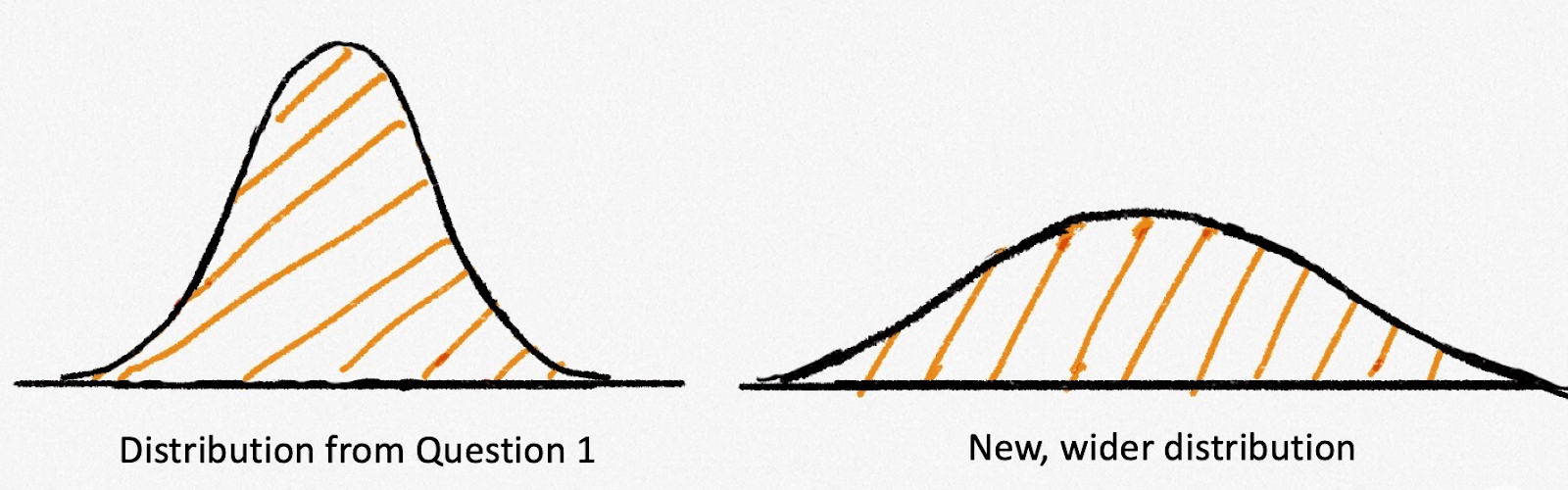


The 24 students who answered Question 3 incorrectly made three main types of errors: (1) 11 students said that the probability would change if the distribution became wider. One of these students said: “*The probability would change to encompass fewer Y values and more X values.*” Another said: “*The original distribution is normal and the wider distribution is not. The empirical rule* [a shorthand to remember the percentage of values that fall within each standard deviation of the normal distribution] *only applies to normal distributions. So indicators of 68% or 2.5% would not exist.*” (2) Seven students did not say whether the probability represented by the shaded region would change or not. For example, one student said only that “*the peak is higher than the wider one.*” (3) The remaining seven students said that the probability would not change, but were not able to provide a sensible explanation of their answer. For example, one said: “ *I don't think it would change, making it wider would only help people clearly see the distinction between the x-axis, but I don't think anything more.*”

Finally, Question 4 tested whether students could use the symmetry property of the normal distribution, together with the knowledge that the entire area under the curve was 1, to reason about probabilities. Students were presented with the normal curve shown in Figure 4. One vertical line marked the mean, which was labeled as 8, and another marked the value 10 on the *x*-axis. The area under the curve greater than 10 was shaded. A standard deviation was not provided. The question read:

*“The probability of a randomly sampled data point being greater than 10 is 0.2. Based on this, what is the probability of a randomly sampled data point being greater than 6? Explain your answer.”*

**Figure 4**

*A Normal Curve with a Mean of 8 and the Region Greater than 10 Shaded, from Question 4*


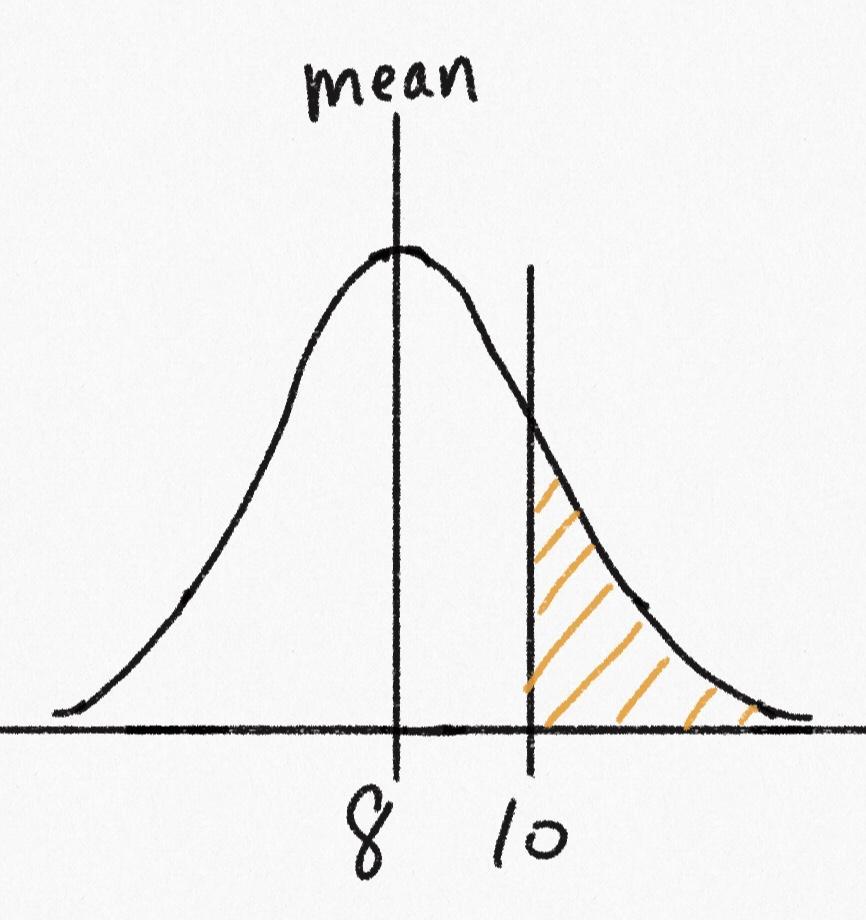


Nearly two-thirds of the sample students (25 students, or 64%) generated incorrect probabilities (the correct answer is 0.8) and provided a variety of explanations. As before, five students erroneously tried using the concept of *Z*-scores or the "empirical rule" to explain their answers. One, for example, said:

“*If the probability of a random data point being greater than 10 is .2, then 10 has a z score of 2. This means that a change in value from 8 to 10 is measured in 2 standard deviations, so 6 to 8 is another 2 z scores. So the probability of a randomly sampled data point being greater than 6 is about 98%, because it is represented by the area of the normal distribution above -2 standard deviations from the mean.”*

Another student said that the probability would be 0.6 “*because there is a z-score of -2*.”

## Discussion

The results of Study 1 shed light on what students might not have understood about the normal probability distribution when they were nearing completion of an introductory statistics course at a highly selective university. Both the content and prevalence of the identified misunderstandings provide important insights into how students in this course interpret drawings of the normal probability distribution. In particular, many students surveyed in this study do not fully and consistently understand how the area under a normal curve can be used to represent probability, or that the total area under a probability distribution would add up to 1.

It is intriguing that more students were able to understand half of the area under the normal curve equates to 0.5 than to understand the entire area under the curve adds up to 1. Future studies could further investigate whether concepts tested in these two questions are fundamentally different or whether there was an order or question specific effect. For example, answering the first question about the entire area under the curve might have caused students to pay attention to some features that might have helped them to answer the second question. Another possibility is that seeing “half of the area” in the visualization cued students to the concept of “half” (i.e., a probability of 0.5). This connection between a visual half and a probability of 0.5 might be stronger than the connection between seeing “the entire area” under a curve and the probability of 1.

Students also failed to infer probabilities based on the symmetric property of a normal probability distribution, often resorting to ideas such as *z*-scores, inappropriately applying unnecessary (albeit strongly associated) statistical concepts to the drawing at hand.

Nonetheless, the current study is exploratory in nature. It was meant to provide qualitative insights of common misconceptions that would guide the design of forthcoming experimental studies. Because we only surveyed 39 students from one class, it remains unclear whether the findings would apply more broadly to, e.g., students in other departments or institutions. What the findings enabled us to do, instead, is to lay out the misconceptions and help us design experiments to test whether these misconceptions could be remedied by interventions based on theories in cognitive psychology. The findings of our next experiments support the validity of the qualitative analysis.

It's worth pointing out that the goal of this study was not to identify misconceptions that are universal. Instead this qualitative approach is a part of our continuous improvement approach to designing learning interventions (Stigler et al., 2020). First, we want to understand the "current state" (Rother, 2009) by identifying the misconceptions students have. We then hypothesize the causes of the misconceptions, design interventions, and only then conduct randomized experiments. This way, interventions can be designed in a targeted fashion and experimental findings can be interpreted in the context of what learners actually need.

Given the results of Experiment 1, we next tested whether learners would benefit from an intervention intended to help them interpret drawings and visual representations of the normal probability distribution. Therefore, in Study 2 and Study 3, we designed experiments to explore whether drawings and other dynamic visualizations could remedy students’ misconceptions about probabilities under the normal curve through a brief instructional intervention.

# Study 2

Study 2 set out to answer two questions: would students understand the normal probability distribution better with the aid of videos that depict drawing (with or without hands) compared to a video with static visualizations? If the answer is yes, would the effect of the intervention produce lasting and transferable knowledge?

We created a brief instructional intervention to provide students with the fundamental knowledge they would need for interpreting visual representations of probability distributions that teachers commonly use to explain more advanced statistical concepts. The focus of the instruction was on the normal curve and its use as a probability distribution for modeling the distribution of a variable. Effects of the intervention were assessed on both immediate and delayed posttests. The instruction was implemented in the form of a brief (15 minutes) video, of which we created three versions (experimental conditions): *Drawing+Hand* (dynamic and embodied), *Drawing Only* (dynamic but not embodied), and *Static Slides* (neither dynamic nor embodied). Students were randomly assigned to view one of the three versions. Based on concerns raised in the previous literature, we tried to equate as much as possible the information contained across the three conditions of the video.

In the *Drawing+Hand* condition, participants watched an instructional video that contained drawings dynamically created by a hand. In the *Drawing Only* condition, participants watched only the screen recording of the drawing without the hand. In the *Static Slides* condition, participants watched a series of static slides depicting computer-made visualizations equivalent to the final state of the drawings in the other videos.

The immediate posttest, described below, was administered right after students viewed the instructional video. A delayed posttest was administered three weeks later. Measuring delayed effects is important for two reasons: (1) to test whether learning lasts and can generalize beyond a single, controlled laboratory session ([Halpern](https://naspa.tandfonline.com/author/Halpern%2C+Diane+F) & [Hakel](https://naspa.tandfonline.com/author/Hakel%2C+Milton+D), 2002; Stigler et al., 2020); and (2) because sometimes the impact of an intervention, especially on tests of transfer, is evident only after a delay (Adams et al., 2014; McLaren et al., 2015).

If dynamic drawing aids learning over and above static presentation of the same content, then both the *Drawing Only* group and the *Drawing+Hand* group would perform better on the posttest than the *Static Slides* group. If embodiment further contributes to learning, then the *Drawing+Hand* group would perform better on the posttest than the *Drawing Only* group (and the *Static Slides* group).

## Method

### Participants

Seventy-nine undergraduate students taking a summer-session introductory statistics course at a large public research institution participated in the study. Of these students, 71 completed the study and took both the immediate and delayed posttests. Eight additional participants were further excluded from the study based on predetermined exclusion criteria, which included (1) spending either less than 400s or more than 7200s on the survey; (2) reporting significant technical difficulties or disruptions while completing the survey (e.g., not having a quiet enough study environment for them to watch the instructional video); or (3) writing the same response for every free response question (e.g., “Not sure”). The final sample consisted of 63 students (16 in *Drawing+Hand*, 23 in *Drawing Only*, and 24 in *Static Slides*). They reflected the ethnic diversity of the campus: 50.8% Asian, 4.8% Black or African American, 12.7% Hispanic or Latino, 23.8% White, and 7.9% multiracial or other.

It is worth noting that because the study was conducted at the outbreak of COVID-19, the class was taught online. As in Study 1, students were offered extra credit to participate in the study. The study was approved by the institutional review board at the university.

Although our sample size was predetermined by the number of students from the statistics course who voluntarily participated, we conducted a power analysis to understand the minimum effect size that could be detected with our sample size using the pwr package in R (Champely et al., 2017; Cohen, 1988). Based on an α of .05, a power of .80, and a sample size of 16 per group (which is the smallest group we had), the minimum Cohen’s f this study could detect was 0.46.

### Design & Procedure

Students who volunteered to participate clicked on a link for a Qualtrics survey, at which point the survey software randomly assigned them to one of the three conditions: *Drawing+Hand*, *Drawing Only*, or *Static Slides*.

All three conditions included an initial survey, followed by an instructional video, a self-judgment about their level of learning, an immediate posttest and, three weeks later, a delayed posttest. In the initial survey, participants completed a pretest with seven questions assessing their understanding of frequency histograms, density histograms, and probability distributions. Then, participants watched a 16-minute instructional video, which varied depending on condition. Students then rated the pace of the video and how much of the video they felt they understood on a scale of 0 to 100. Afterward, they answered 15 posttest questions, three survey questions that elicited their opinions of the video, and one screening question asking if anything went wrong during the experiment.

Three weeks later, when students took their final course exam, they were informed that they could get additional extra credit by taking the delayed posttest. Students participated voluntarily in this activity. Importantly, the material covered in the introductory statistics course between the immediate and delayed posttests, which focused on constructing statistical models to explain variation, did not vary across experimental groups.

***Materials***

For the *Drawing+Hand* condition (Figure 5, left), we videotaped a hand as it was drawing illustrations on an iPad; to this end, we used an external camera that captured both the hand and the iPad’s screen. We then recorded a voice narration to accompany the video. This format resulted in a drawing video that was both dynamic (unfolding through time) and embodied (showing the body part that generates the drawing).

For the *Drawing Only* condition, we used the same audio track, but this time paired it with the iPad's screen recording of the drawing as it was being produced for the drawing hand condition. Thus, the only difference between these first two conditions was in whether the hand could be seen producing the drawing or not, which allowed us to gauge the effect of this minimal level of embodiment over and above the effect of the dynamic representation (i.e., drawing unfolding over time) without the hand.

Finally, the *Static Slides* condition used the same audio track, but instead of showing the drawing unfold dynamically over time, it displayed a series of static slides. The slides were designed to match the final state of different illustrations in the other two conditions, but were produced using standard, computerized drawing tools such as those included in PowerPoint. For example, the two drawing videos contained a manually drawn histogram while the static slides video contained a histogram made using R and PowerPoint. All videos were accompanied by the same audio track. (See Figure 5 for several screenshots from each condition.) All

## video materials can be accessed through the OSF wiki page: <https://osf.io/af3p9/?view_only=e0668f936b584577b2b5ffacb66d6d2f>.

**Figure 5**

Screenshots of Videos from the Three Experimental Conditions.

|  | 13:48: “a common misunderstanding is to think the probability of getting a number higher than the mean is always 0.5” | 13:55: “this is true only when the distribution is normal” | 14:07: “If the distribution is skewed to the left, the probability… is less than 0.5” |
| --- | --- | --- | --- |
| Drawing+Hand | 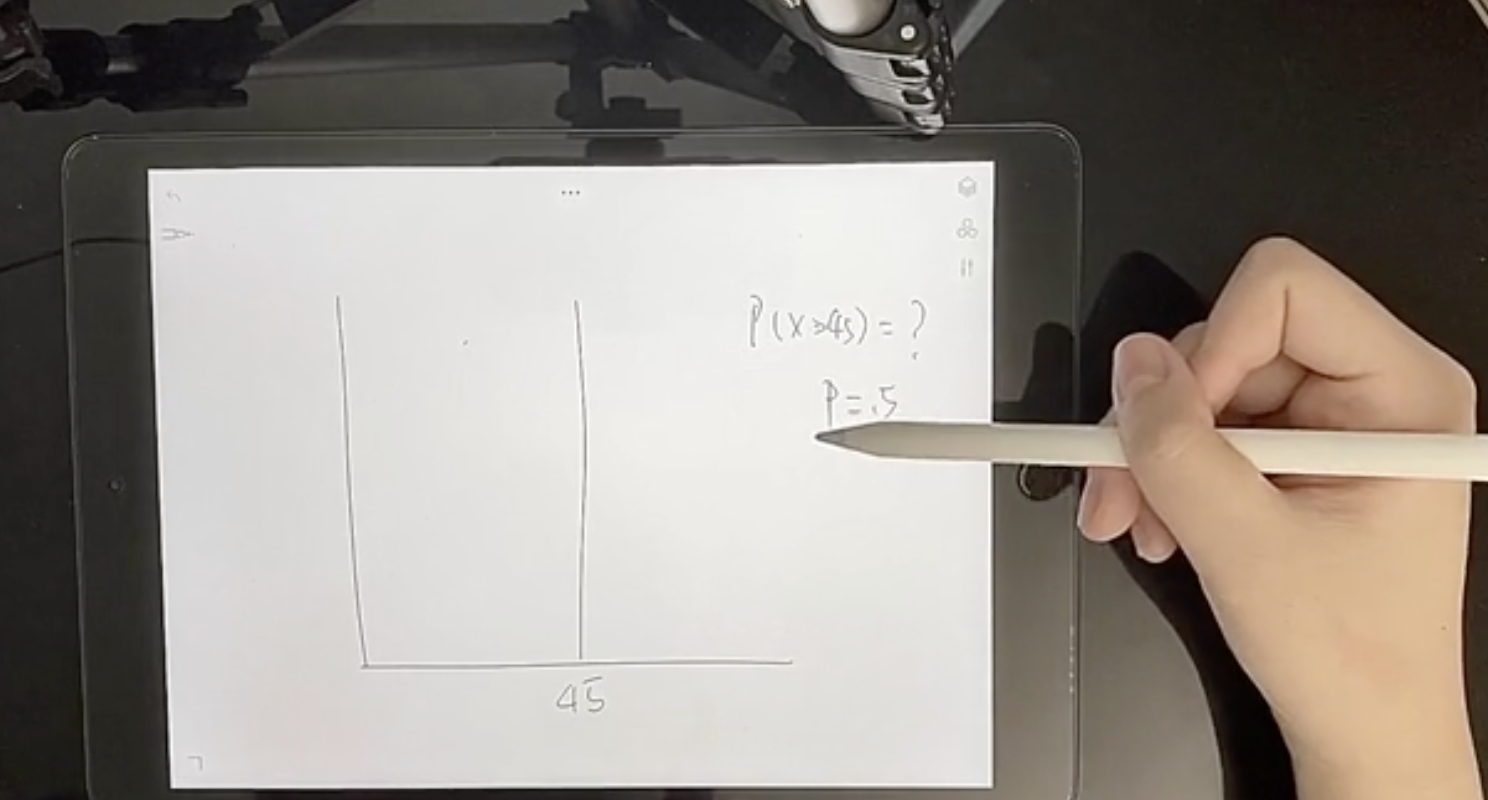 | 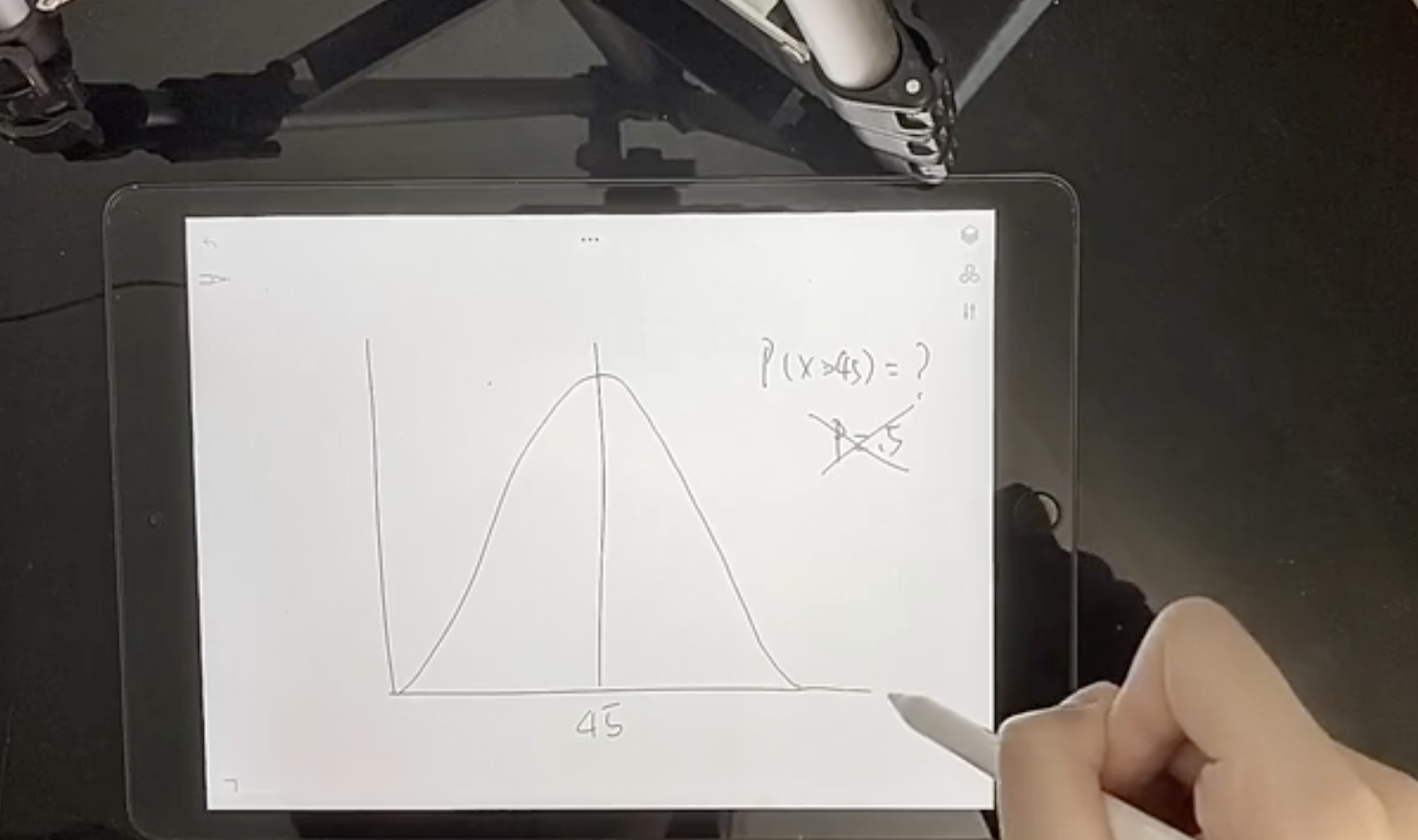 | 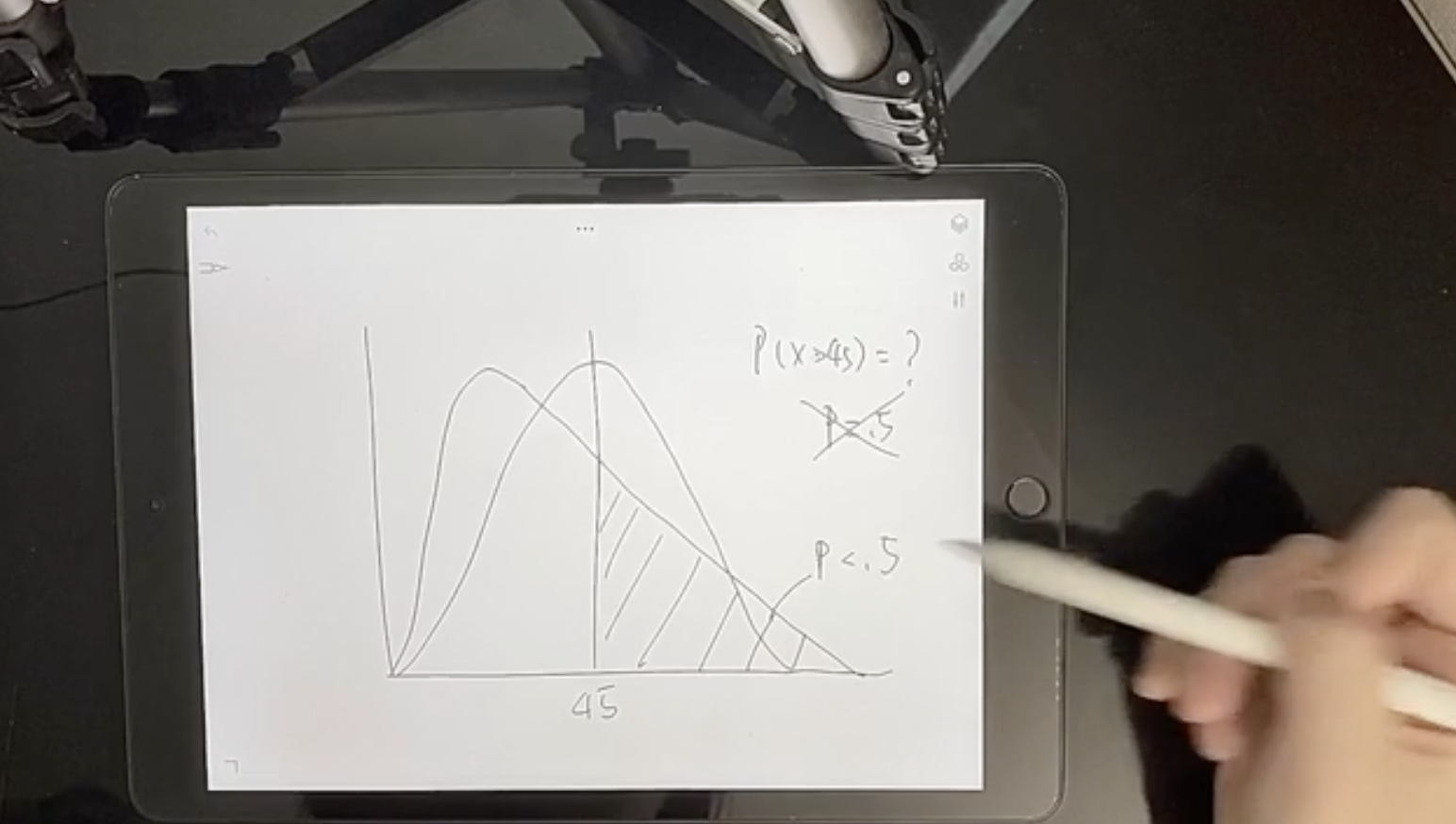 |
| Drawing Only | 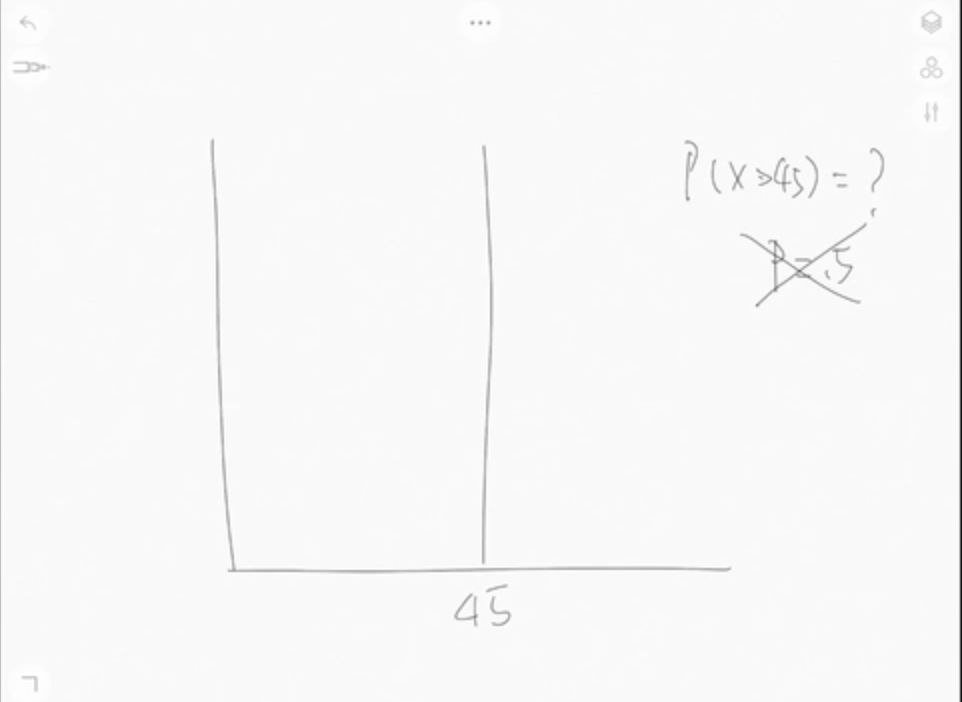 | 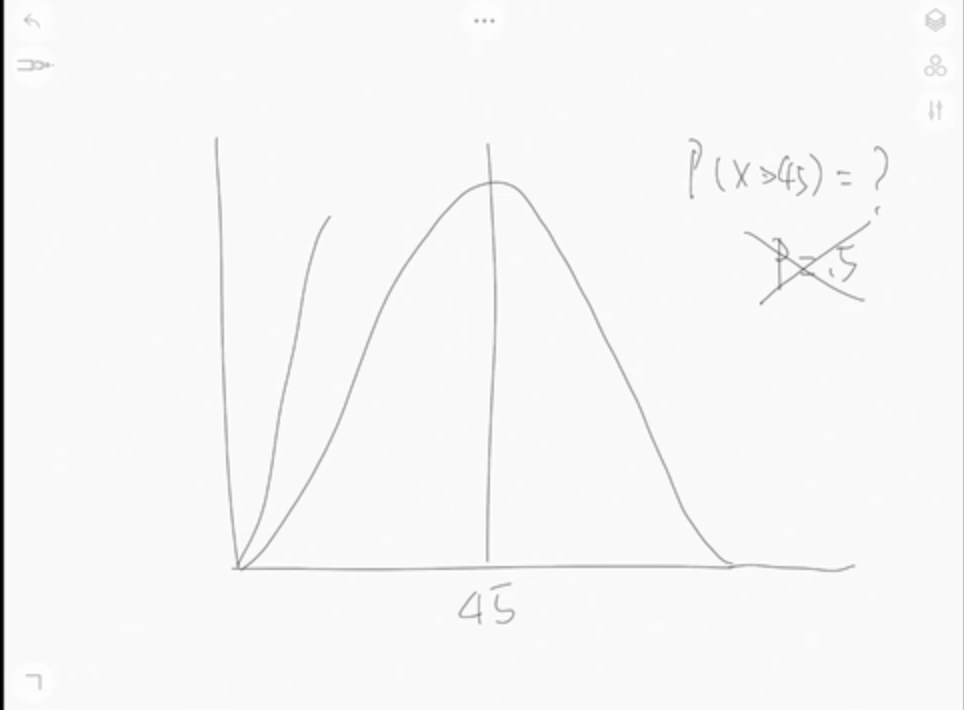 | 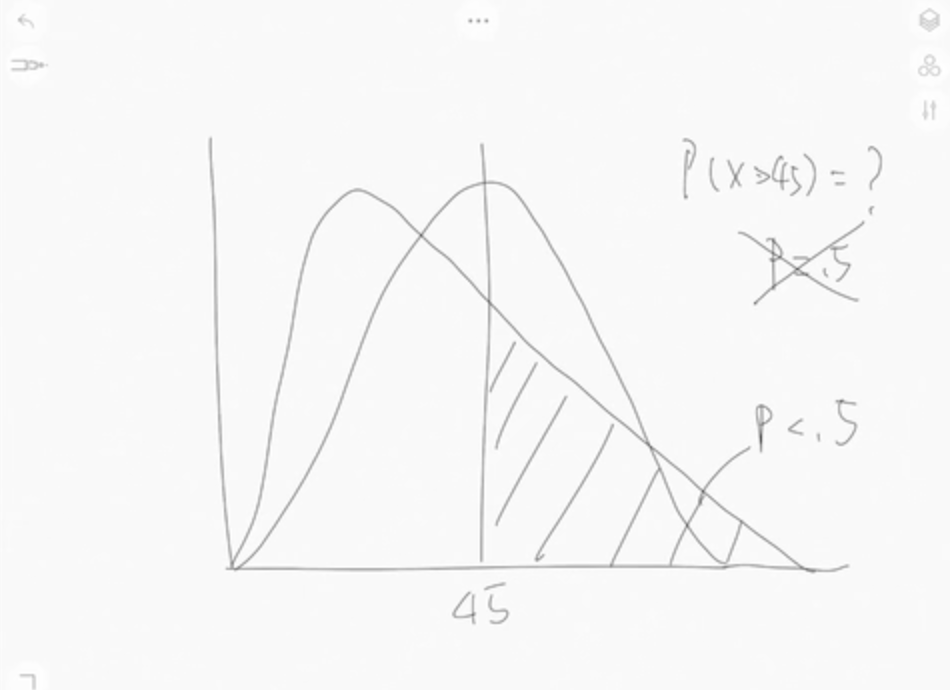 |
| Static Slides | 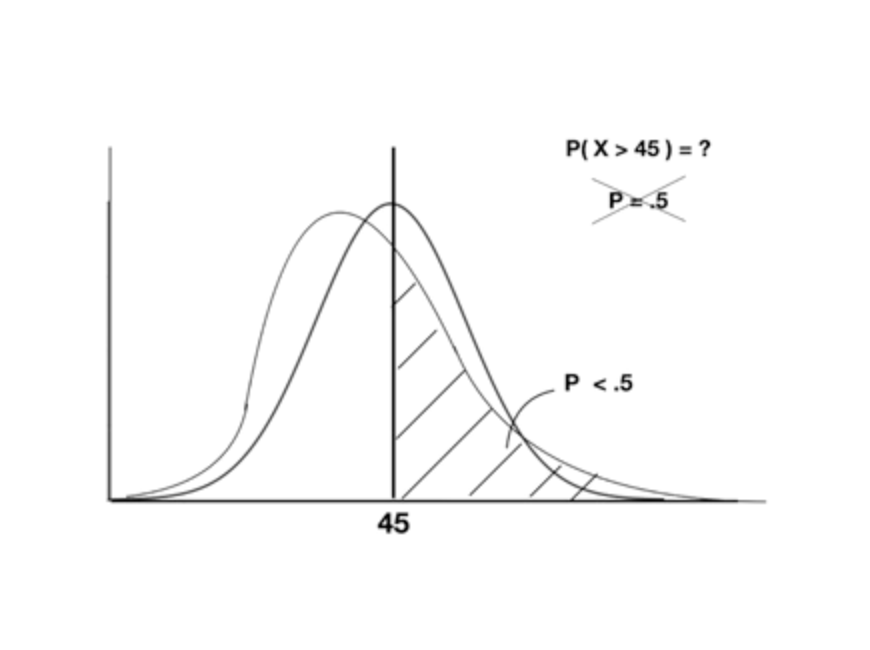 | 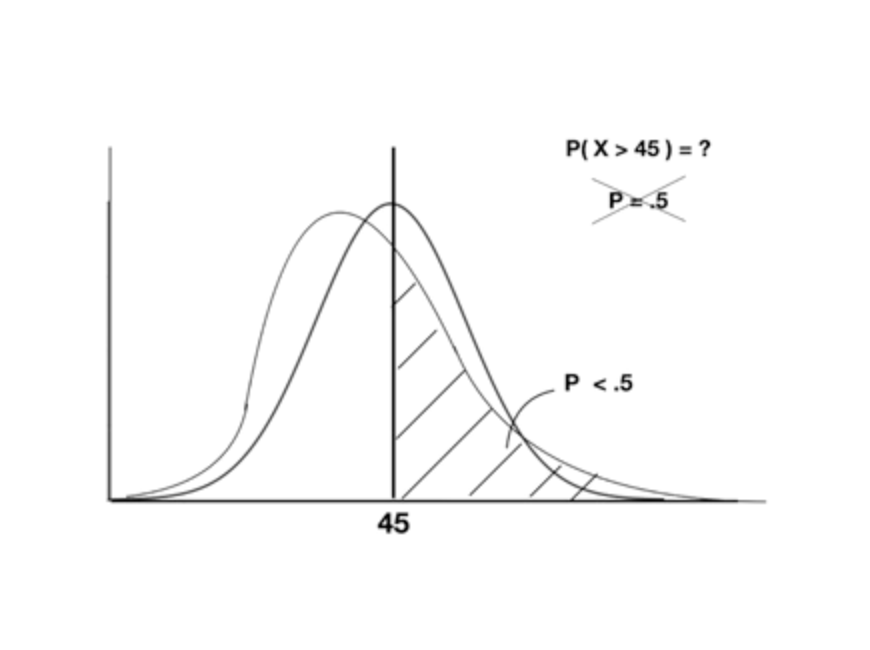 | 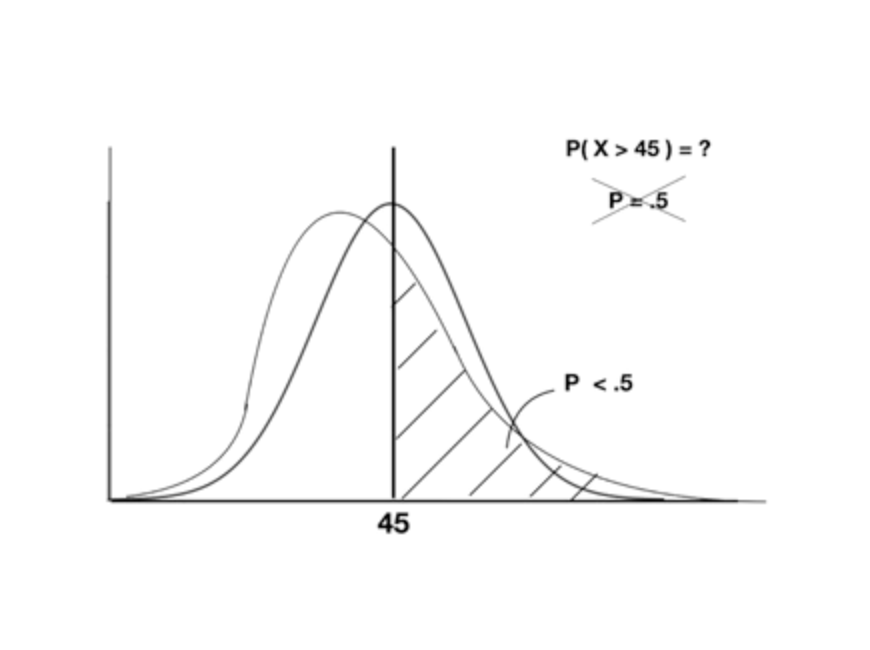 |

## *Measures*

### Pretest. The pretest contained seven questions designed to assess participants’ existing knowledge of normal probability distributions. Four questions were the exact same questions as in Study 1. All seven pretest questions were also included in the immediate posttest. Four of the questions were included in all three tests: pre-, post-, and delayed posttest. A full list of these questions is included in Appendix B.

***Accuracy of Judgment of Learning.*** After watching the video, participants rated their understanding of the video on a scale of 0 to 100 in percentage terms. Following previous literature’s convention, we calculated the accuracy of participants' judgment of learning by subtracting actual post-test performance (between 0% and 100%) from self-rated understanding (between 0% and 100%) (i.e., the bias measure; Griffin et al., 2009; Maki et al. [2005](https://link.springer.com/article/10.1007/s12144-013-9198-x#ref-CR28)). For example, an overly confident participant who rated their understanding to be 80% and scored 70% correct on the post-test would obtain a score of 80%-70%=10% for their accuracy of judgment of learning. Thus, 0% corresponds to perfect accuracy of judgment of learning, a positive score indicates overestimation of learning, and a negative score indicates underestimation.

***Immediate Posttest.*** The immediate posttest contained 17 questions, 7 of which were identical to those on the pretest. These questions were designed based on students’ misconceptions identified in Study 1, which the instructional videos were designed to address. In addition to directly asking students to recall the concepts, we also designed questions where students needed to apply what they have learned about normal probability distributions to novel contexts. The questions were a combination of multiple choice and free responses questions designed to assess students’ conceptual understanding of areas under the normal curve and their corresponding probabilities, the symmetry of the normal distribution, the relationship between curve width and probabilities, the features of a faceted histogram, and the use of the normal distribution as a data-generating model. A complete list of these questions is included in Appendix C. Cronbach’s alpha for the 17 questions was .73.

***Delayed Posttest.*** The delayed posttest contained 17 questions. Seven of these questions were duplicates of questions included on the immediate posttest, and the rest were new questions that required students to engage in inference and transfer (see Appendix D). Cronbach’s alpha for the 17 questions was .76.

### Scoring of Tests

Three trained coders, blind to each participant's experimental condition, scored students’ responses on the pretest, the immediate posttest, and the delayed posttests independently from each other. Each question was randomly assigned to be scored by two of the three coders. Disagreements in scoring were discussed in a group meeting, also blind to condition, until a consensus was reached. Coders reached an average of 89% consistency after the first round of coding. The final consistency rate after the group meeting was 100%.

Participants were given one point for each correct response. Summary scores for each participant and each of the three tests were calculated by summing the points earned across all questions of that test. Scores on the pretest, therefore, could range from 0 to 7 and, on each of the two posttests, 0 to 17. In the figures below (Figure 6, 7, and 8), we present the percentage of correct responses instead of raw scores to facilitate comparisons across the tests.

## Results

### Video Ratings

On average, participants rated their understanding of the video they watched as 76.6 on a 100-point scale (SD = 17.5). The mean rating for the *Drawing+Hand* group was 77.5 (SD = 13.5), for the *Drawing Only* group, 80.52 (SD = 17.2), and for the *Static Slides* group, 72.3 (SD = 19.8). A one-way ANOVA revealed no significant difference across the three conditions (*F*(2,60) = 1.35, *p* = .267, *η^2^* = .00).

***Pretest Performance***

Participants on average correctly answered 70% of the pretest questions (i.e., 4.87 of 7 questions). The *Drawing+Hand* group answered 71% of the questions correctly (SD = 0.24); the *Drawing Only* group answered 67% of the questions correctly (SD = 20); and the *Static Slides* group answered 70% of the questions correctly (SD = 0.27). The three groups did not differ significantly on their pretest performance (*F* (2,60) = .153, *p* = .858, *η^2^* = 0.01).

***Accuracy of Judgement of Learning by Condition***

Figure 6 shows participants’ accuracy of judgment of learning. (Note that both positive and negative numbers are less accurate than 0.) Descriptively, the *Drawing+Hand* group’s judgment of learning is the closest to 0. A one-way ANOVA did not find a significant effect of condition on participants’ accuracy of judgment of learning (*F*(2,60) = 2.56, *η^2^*= .08 , *p* = .086). Post-hoc pairwise comparisons revealed that the *Drawing+Hand* group was significantly different in their judgment of learning compared to both the *Drawing Only* group (*t*(60) = 3.08, *p_adj_* = .009) and the *Static Slides* group (*t*(60) = 2.53, *p_adj_* = .041). There was no significant difference in accuracy of judgment of learning between the *Drawing Only* group and *Static Slides* group (*t*(60) = 0.63, *p_adj_* = 1). (Note: the *p*-values were adjusted for multiple comparisons using Bonferroni.)

We also tested, using one sample t-tests, whether each group’s average judgments of learning were significantly different from 0. The Static Slides group significantly overestimated their learning (*t*(23) = 2.72, *p* = .012), as did the Drawing Only group (*t*(22) = 4.00, *p* < .001). The Drawing+Hand group’s judgment of learning did not significantly differ from 0 (*t*(15) = 0.18, *p* = .863).

**Figure 6**

*Accuracy of Judgement of Learning by Condition*


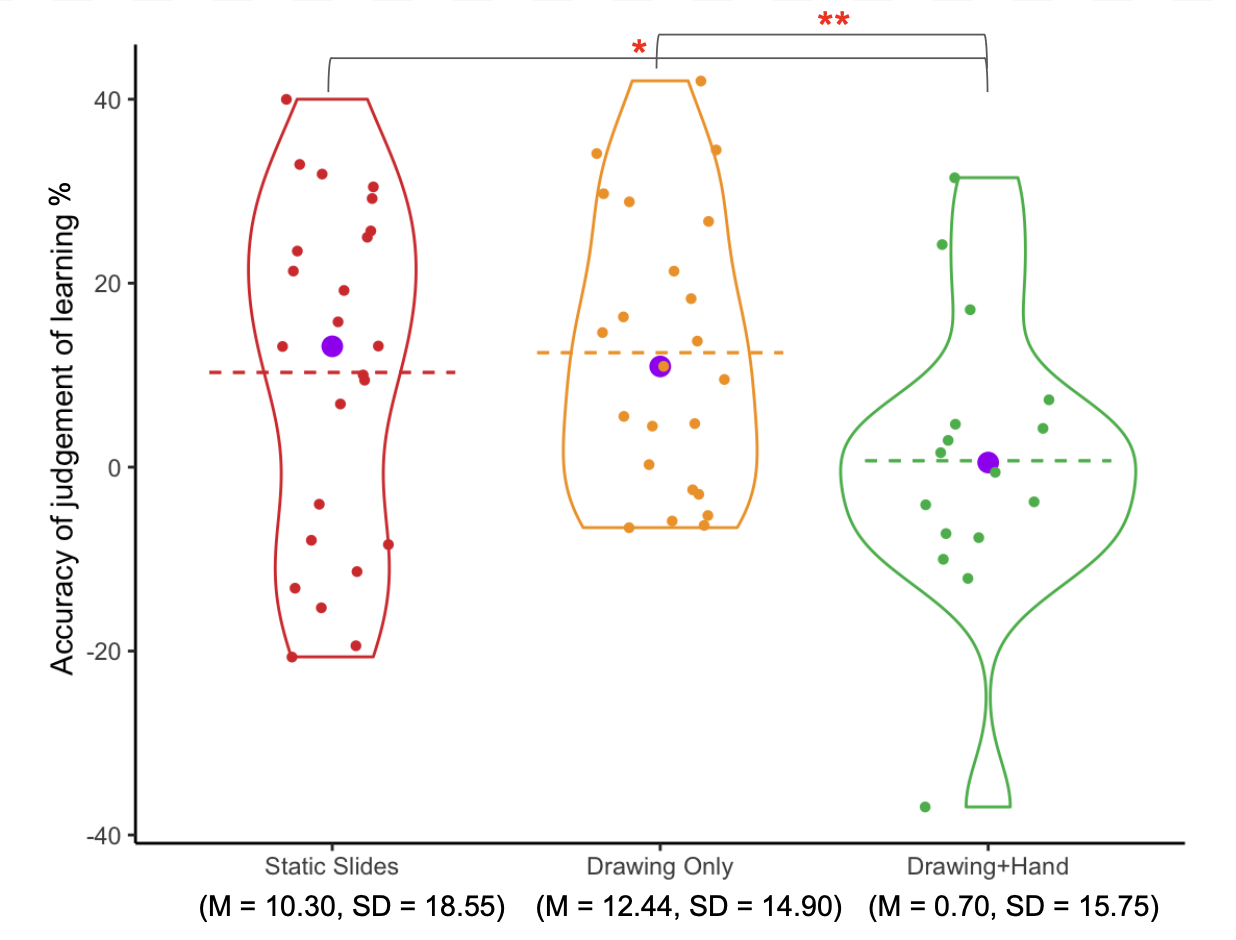


### Immediate Posttest Performance

Scores on the immediate posttest for each of the three conditions are shown in Figure 7. Students in the *Drawing+Hand* condition had higher scores, on average, than those in the other two conditions. We used a one-way ANalysis of COVAriance (ANCOVA) to explore the impact of condition on students’ immediate posttest performance while controlling for performance on the pretest. The complete ANCOVA table is shown in Table 3. The one-way ANCOVA revealed a significant difference in posttest performance across the three groups (*F* (2,59) = 4.57, *p* = .014, *η_p_^2^* = .14; Levene’s test and normality checks were carried out, and the data met the assumptions).

**Table 2**

*Descriptive Statistics Immediate and Delayed Posttest Performance for three Conditions*

| Measure | Static Slides  (n=24) | | Drawing Only  (n=23) | | Drawing+Hand  (n=16) | |
| --- | --- | --- | --- | --- | --- | --- |
|  | M | SD | M | SD | M | SD |
| Immediate Posttest Score | 11.77 | 3.44 | 12.93 | 2.94 | 14.59 | 2.40 |
| Delayed Posttest Score | 8.96 | 3.14 | 9.91 | 3.50 | 11.03 | 2.97 |

**Table 3**

*ANCOVA Results (Study 2, Immediate Assessment)*

|  | *df* | *MS* | F | *η_p_^2^* | PRE | p |
| --- | --- | --- | --- | --- | --- | --- |
| Condition | 2 | 0.10 | 4.57 | .14 | .13 | .014 |
| Pretest Performance | 1 | 0.19 | 8.51 | .13 | .14 | .005 |

##

We conducted post-hoc pairwise analysis using the "supernova" package in R (version 2.5.6; Blake et al., 2023) using pooled error variance. Post-hoc pairwise comparisons of immediate performance controlling for pretest performance showed that the *Drawing+Hand* group significantly outperformed both the *Static Slides* group (*t* (59) = 4.34, *p_adj_* <.001) and the *Drawing Only* group (*t* (59) = 2.53, *p_adj_* = .042). There was no significant difference between the *Static Slides* group and the *Drawing Only* group (*t* (59) = 1.98, *p_adj_* = .157). (Note: the *p*-values were adjusted for multiple comparisons using Bonferroni.)

**Figure 7**

*Violin Plots Showing Immediate Posttest Scores by Condition.*

**
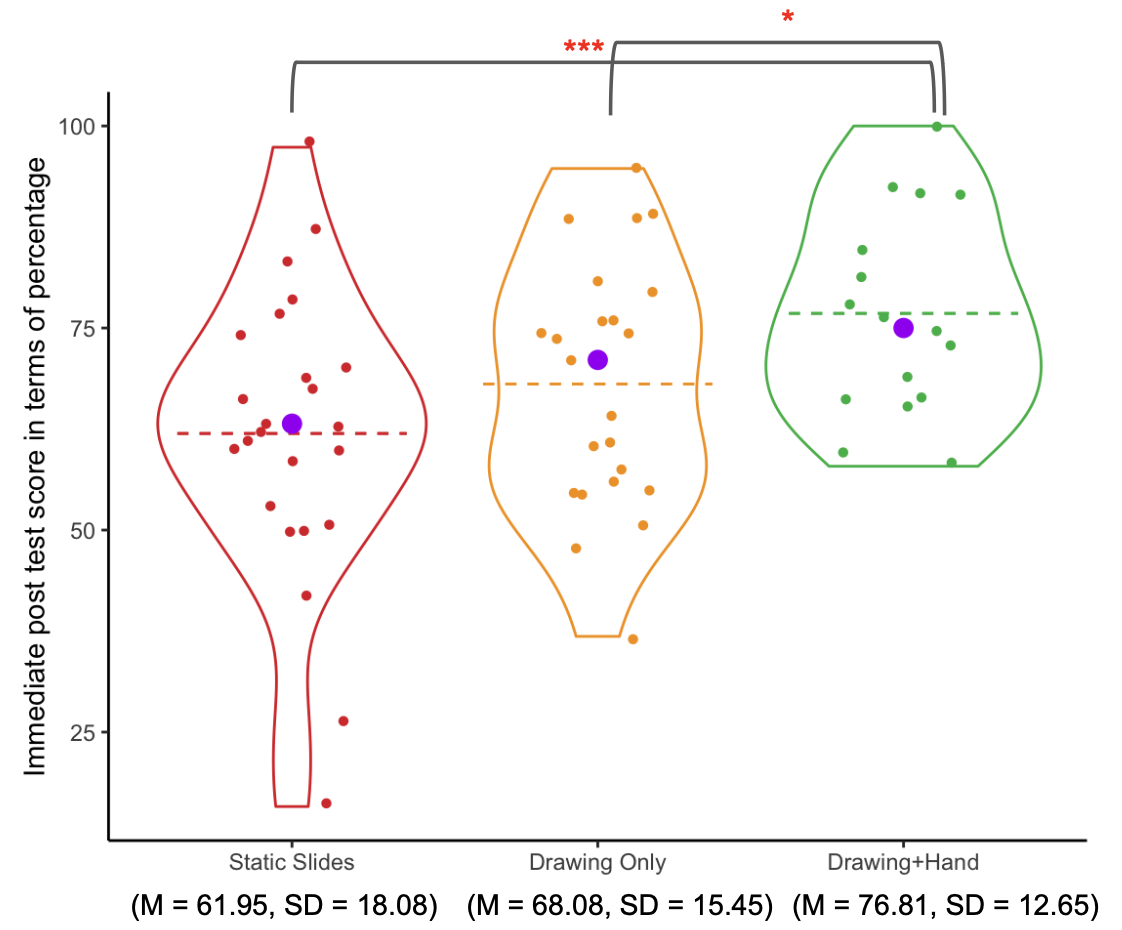
**

*Note.* Dashed lines are means; purple dots are medians.

**p* < .05. ***p* <. 01. ****p* < .001, two tailed with Bonferroni correction.

### Delayed Posttest Performance

Scores on the delayed posttest for each of the three conditions are shown in Figure 8. A summary of students’ immediate and posttest performance by condition is shown in Table 2. Descriptively, the ordering of the three groups remained the same as for the immediate posttest, with the *Drawing+Hand* group scoring the highest, and the *Static Slides* group the lowest. A one-way ANCOVA controlling for pretest performance found no significant differences across conditions (*F* (2,59) =2.03 *p* = .140, *η_p_^2^* = .06; Table 4).

**Table 4**

*ANCOVA Results (Study 2, Delayed Assessment)*

|  | *df* | *MS* | F | *η_p_^2^* | PRE | p |
| --- | --- | --- | --- | --- | --- | --- |
| Condition | 2 | 0.06 | 2.03 | .07 | .06 | .141 |
| Pretest Performance | 1 | 0.13 | 4.08 | .06 | .06 | .048 |

Whereas the ANCOVA evaluated the data against the hypothesis that the group means of the three populations were identical, we were also interested in specific differences between particular groups. In some cases, post-hoc tests can be powerful enough to find significant differences between group means even if the overall ANOVA has a p-value greater than the defined significance level (Hsu, 1996; Maxwell et al., 2017). Post-hoc pairwise comparisons with Bonferroni corrections while controlling for pretest performance were used to provide a more focused and powerful analysis of whether the *Drawing+Hand* group performed better than any of the other two groups. The analyses revealed a significant difference between the *Drawing+Hand* group and the *Static Slides* group (*t*_(59)_ = 2.88, *p_adj_* = .017), but not between the *Drawing+Hand* group and the *Drawing Only* group (*t*_(59)_ = 1.54, *p_adj_* = .388), nor between the *Static Slides* group and the *Drawing Only* group (*t*_(59)_ = 1.47, *p_adj_* = .445). (Note: the *p*-values were adjusted for multiple comparisons using Bonferroni.)

**Figure 8**

*Violin plots showing delayed posttest scores by condition*

**
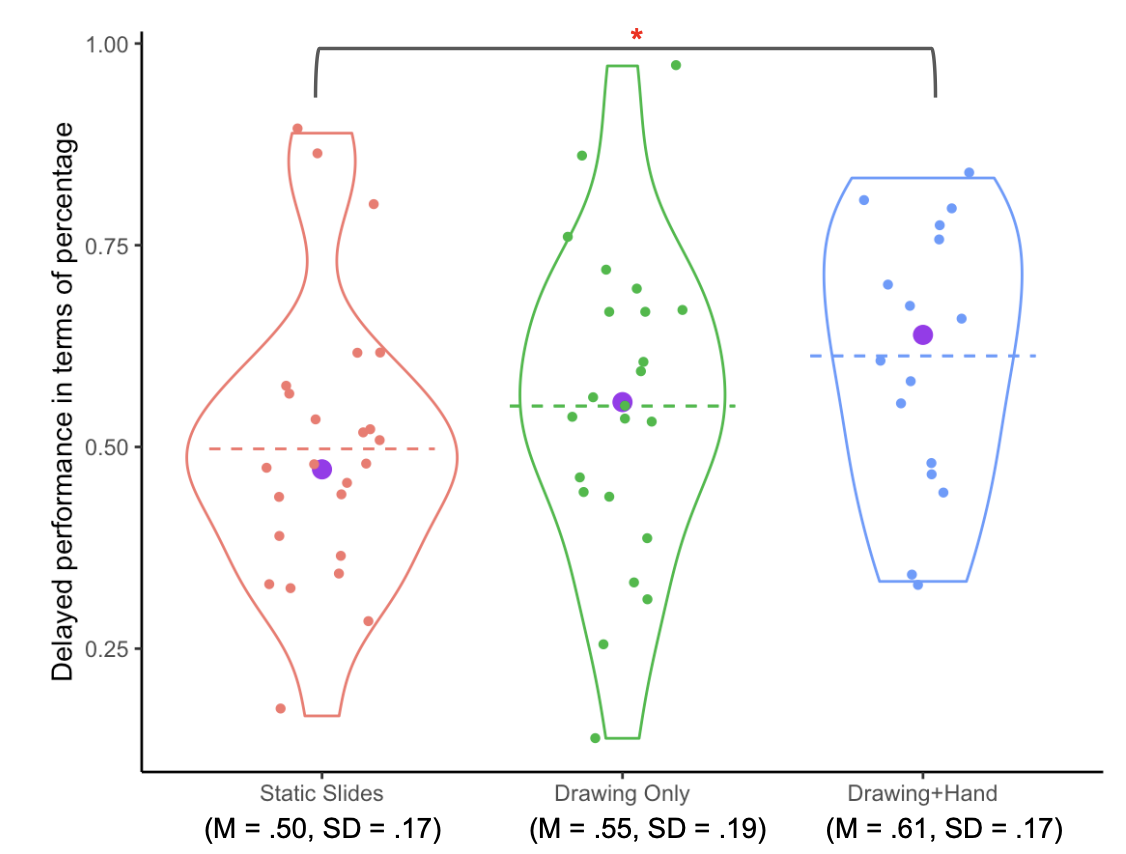
**

*Note.* Conventions are the same as in Figure 6.

## Discussion

In Study 2, we used a brief intervention to teach students concepts related to probability distributions. Specifically, we created three versions of instructional videos: a *Drawing+Hand* video, being both dynamic and embodied; a *Drawing Only* video, being dynamic but not embodied; and a *Static Slides* video showing static, computer-generated images. We found that the *Drawing+Hand* video improved students’ immediate posttest performance and resulted in more accurate judgments of learning compared to the other conditions. Three weeks after the intervention, only the difference between the *Drawing+Hand* and the *Static Slides* group remained statistically significant.

This pattern of results provides evidence for the potency of presenting drawings in a manner that accurately reflects the process by which they were created, with a hand drawing dynamically over time. We hypothesize that drawing data distributions dynamically with a hand may direct attention more effectively over time to different components of the data visualization, and may even give students more time to put these components together.

The finding that the *Drawing+Hand* group outperformed the *Drawing Only* group on the immediate assessment can be interpreted in two ways. First, it points to the possibility that the inclusion of the hand/body might play a unique role in facilitating learning from drawings. Although it is possible that both the *Drawing+Hand* video and the *Drawing Only* video were "embodied" in that they showed drawings that were indeed generated by a human hand, we maintain that the *Drawing+Hand* video was more embodied. The results of the immediate posttest suggest that additional embodiment can be beneficial to learning. Findings from the embodied cognition literature provides support for the speculation that viewing the human hand activates forms of cognitive processing that are not otherwise present (i.e., sensorimotor representations that include the bodily movements of the instructor), resulting in better learning outcomes (Wilson, [2002](https://cognitiveresearchjournal.springeropen.com/articles/10.1186/s41235-018-0092-9#ref-CR90); see Risko & Gilbert, [2016](https://cognitiveresearchjournal.springeropen.com/articles/10.1186/s41235-018-0092-9#ref-CR69), for an overview). This interpretation is also consistent with Mayer's multimedia learning principles, in which embodiment such as drawing with a visible hand is hypothesized to help learning, especially when it guides or activates helpful cognitive processes (Mayer, 2014).

However, an alternative interpretation, given that both videos are embodied to some degree, is that the presence of the hand primarily served to direct attention to the drawing process. Perhaps having the hand holding a pointy writing device is like having an additional arrow directing the viewer's attention. If that is the case, similar learning benefits might also be achieved by, for example, having an enhanced version of the PowerPoint Slides in which a cursor moves to direct learners’ attention to specific parts of the drawings. This is the idea we tested in Study 3.

It is also worth keeping in mind that there was a difference between what we observed from the immediate assessment and the delayed assessment. The *Drawing+Hand* group did not significantly outperform the *Drawing Only* group after three weeks on a delayed assessment as they did on the immediate assessment, though the overall pattern of means was similar between the two measures. There are two potential explanations for this.

First, this might simply be a result of insufficient power. The recruitment challenges imposed by COVID-19 hindered our ability to meticulously control and attain the desired sample size suggested by a priori power analysis. Our power analysis revealed that with a power of 0.8, our sample size was only sufficient for detecting a very large effect (f = 0.43). Even if a delayed effect did exist, this study might be underpowered to detect such an effect, particularly if the effect size is smaller than that of a large effect.

Second, it is also possible that the effects of embodiment (over and above those of dynamic visualizations) are short-lived. In general, studies should always consider whether the effects of pedagogical techniques would sustain after a delay. Further studies are needed to understand the effects of watching drawings after a realistic period of delay.

Neither the immediate nor the delayed assessment found a significant difference between the *Drawing Only* group and the *Static Slides* group. This is consistent with some findings in dynamic and static visualizations showing no clear benefits of dynamic visualizations over static visualizations (e.g., Tversky et al., 2002). However, there are studies that have found a significant difference between these two types of visualizations. For example, Zhu and [Grabowski](https://www.learntechlib.org/author/Barbara+Grabowski) (2006) found that dynamic visualizations benefited learners with low prior knowledge more than static graphs. Future research might consider looking into how characteristics of the learners interact with the different efficacy of these two types of visualizations. We also note that the static slides we used were somewhat more “cleaned-up” and professional-looking than the drawings used in the other two conditions. The neatness of the static slides might also help learners, compensating for the missed effect from dynamic visualizations.

Study 2 also explored whether drawings (embodied or not) would lead to greater metacognitive judgments of learning. The *Drawing+Hand* group exhibited more accurate judgments of learning compared to the other two groups. Notably, participants in the *Drawing Only* and *Static Slides* groups tended to display more overconfidence in their learning compared to the *Drawing+Hand* group. Although this finding is largely exploratory, this suggests that the embodied benefits to learning may tap into mechanisms connected to metacognitive judgments of learning. Further investigations are warranted to eliminate alternative explanations and explore how judgments of learning might benefit from embodied drawings.

Together, the findings of Studies 1 and 2 offer promise to statistics education, by suggesting that students’ understanding of normal probability distributions can be improved with a brief drawing intervention that could be delivered online, outside of class time, potentially providing students with a scaffold for future learning. This finding may also prompt instructors to consider the use of whiteboards and chalkboards available in their classrooms. Although clean, professional-looking visualizations similar to those found in our *Static Slides* condition might be common in statistics courses, perhaps the more “messy looking” dynamic and embodied drawings might offer more benefits in terms of student learning. These results should also inform instructors who create instructional videos. Going slightly beyond screen captured drawings by including visible hands may be worth the benefits to student learning.

Humans take in information from the environment simultaneously through multiple modalities, but this type of information processing is not always the most efficient in learning scenarios. Sometimes, processing information from multiple modalities creates a split of attention, which sabotages learning (Ayres & Sweller, 2005). Nonetheless, our hand-drawing instructional video, instead of being harmed by its multimodal nature, helped learners process information. These findings pave the way for the development of innovative instructional approaches that leverage multimodal learning strategies (e.g., dynamic and embodied representations) to address various domains of knowledge acquisition.

One important lingering question from Study 2 is whether the presence of the hand merely served as a way to direct attention to parts of the drawing as it unfolded during the drawing processes. If the *Drawing+Hand* video was better than the *Drawing Only* video only because the hand was directing students' attention, the benefits achieved by the *Drawing+Hand* video might be similarly achieved with an instructional video that uses something other than a hand to direct attention.

# Study 3

In Study 3, we explored whether the addition of a dynamic cursor to direct attention to specific parts of the computer-generated images might improve the effectiveness of the *Static Slides* video. We again compared students’ learning among three instructional videos. One video was the same as in Study 2, namely, the *Drawing+Hand* video, which was found to produce the greatest learning in that study. The second video was similar to the *Static Slides* video from Study 2, but was “enhanced” to include a moving cursor. Because this change made the video more dynamic, we refer to this condition as the *Dynamic Slides* condition. We included this condition for two reasons: First, to test whether the role of embodied representations—in this case, the hand in the *Drawing+Hand* condition—was simply to guide students’ attention; adding the moving cursor would similarly guide attention, but without relying on an embodied representation. Second, this version of the video has higher ecological validity compared to the *Static Slides* video, because it mimics the way instructors actually use slides in educational settings – pointing, highlighting, moving a cursor, etc.

The third video was a control condition where students watched an unrelated statistics video. This control condition was added to assess the effect of answering the pretest questions before the posttest. The inclusion of this control condition allows us to examine whether either video (*Dynamic Slides* or *Drawing+Hand*) produced an effect larger than a testing effect (wherein people improve in their answers if they have been tested on similar concepts before; for a review, see Rowland, 2014). In this condition, students watched a video about regression in which no mention was made of probability distributions or the normal curve. (For more information about the videos, see the OSF wiki page: <https://osf.io/af3p9/?view_only=e0668f936b584577b2b5ffacb66d6d2f>).

**Figure 9**

*Screenshots of Videos from the Two Experimental Conditions for Study 3. Note that, in the Dynamic Slides condition, the position of the cursor varies across the three screenshots. (The control condition was not included because the narration was completely different.)*

|  | 13:48: “a common misunderstanding is to think the probability of getting a number higher than the mean is always 0.5” | 13:55: “this is true only when the distribution is normal” | 14:07: “If the distribution is skewed to the left, the probability… is less than 0.5” |
| --- | --- | --- | --- |
| Drawing+Hand | 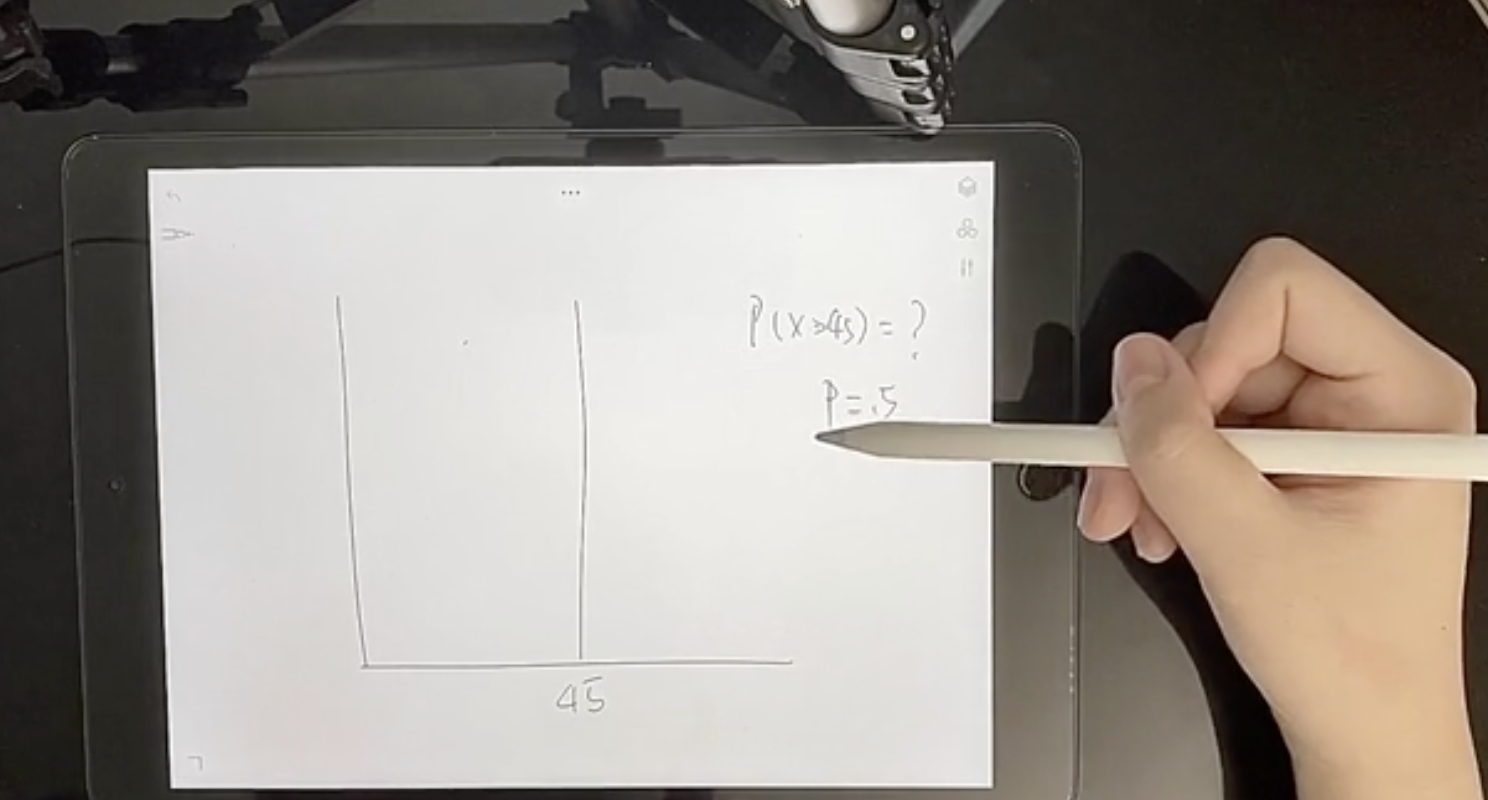 | 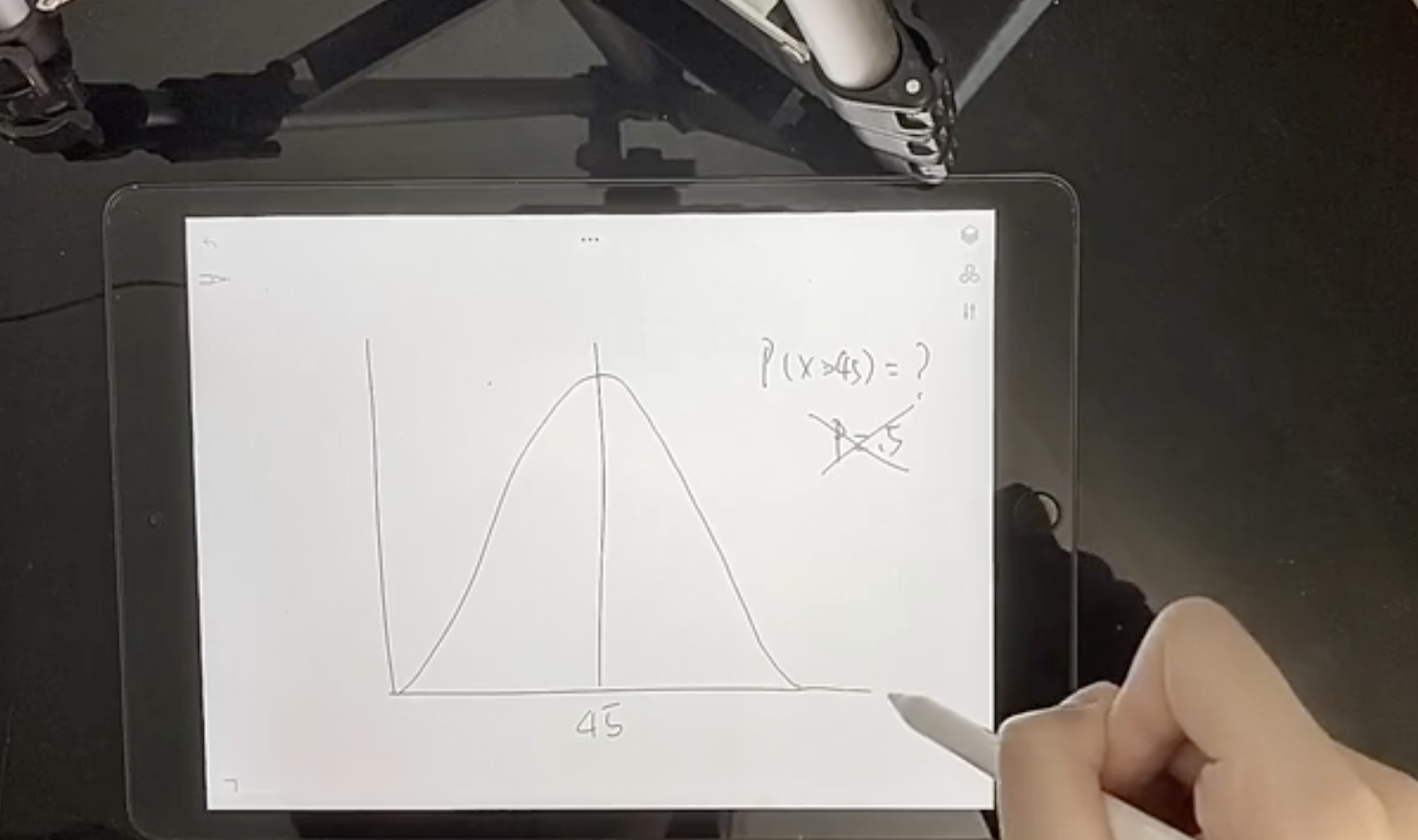 | 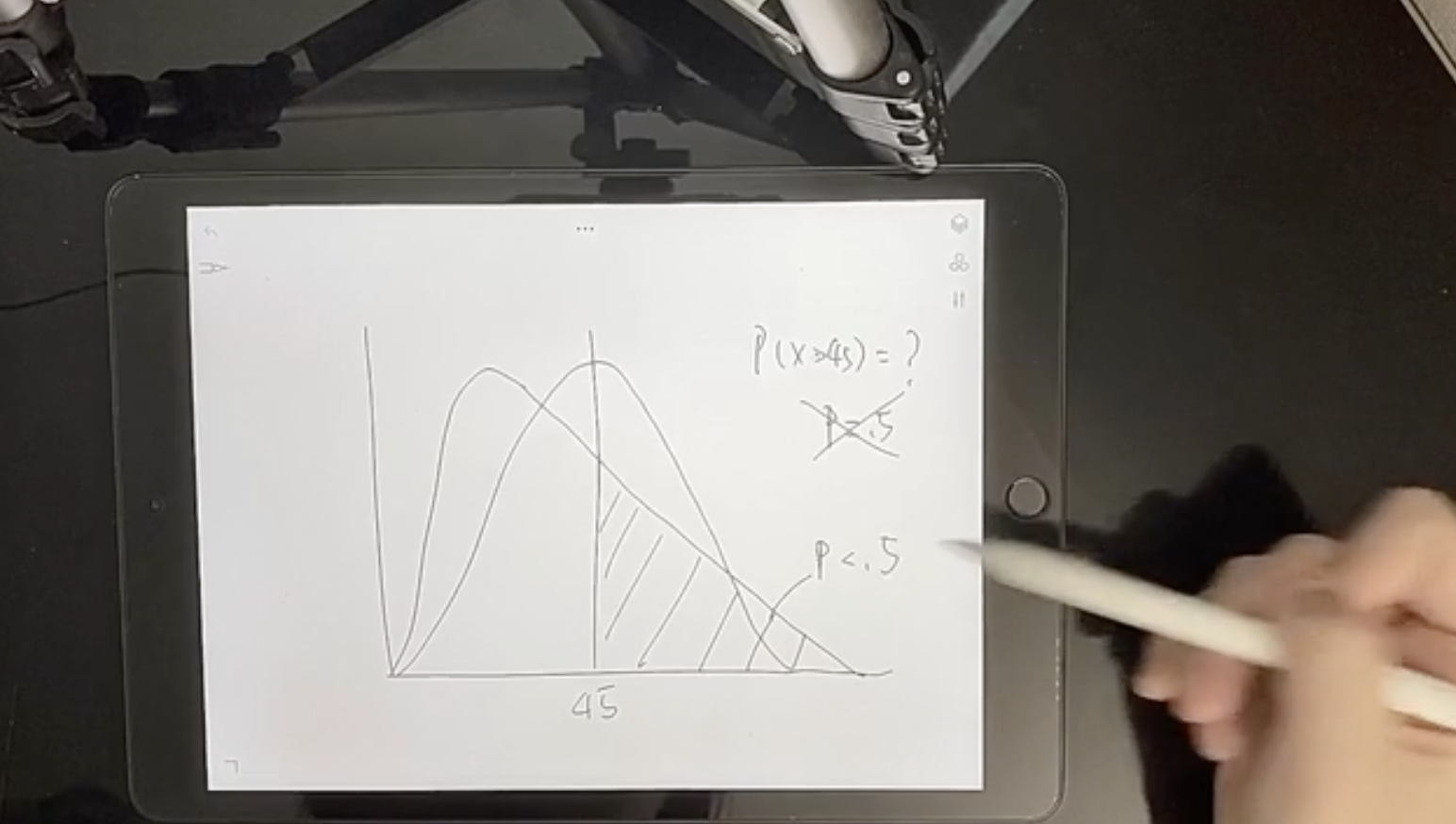 |
| Dynamic Slides | 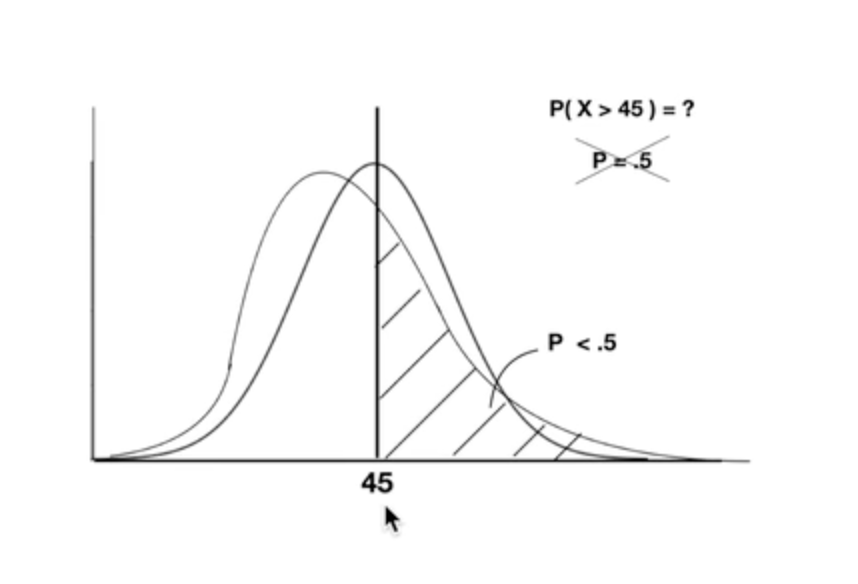 | 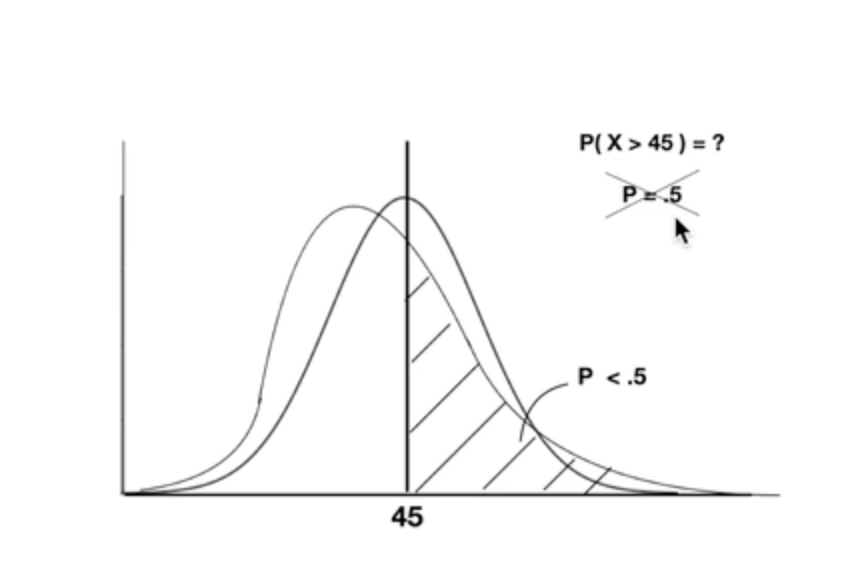 | 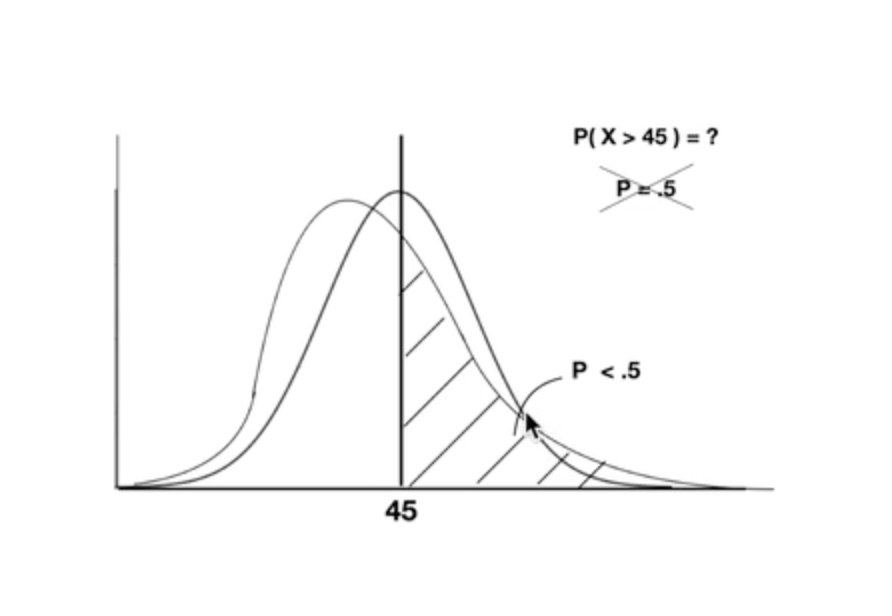 |

We hypothesized that the *Drawing+Hand* group, whose video included embodied information, would perform better than the control group. We also expected the *Dynamic Slides* group to perform better on the posttest than the control group. Of main interest was whether the addition of a dynamic cursor in the *Dynamic Slides* would improve learning to the point of equaling that in the *Drawing+Hand* group.

## Method

### Participants

Participants were 103 undergraduate students taking an introductory statistics course at the same public research institution. Similar to the previous two studies, students were also taking the course online because of COVID 19. Eleven participants were excluded from the study based on the same predetermined exclusion criteria as those used in Study 2, yielding a final sample of 92 participants (*Drawing+Hand*: *n*=33, *Dynamic Slides*: *n*=30, control: *n*=29). Following the same criteria of the power analysis conducted in Study 2 (an α of .05, a power of .80), the obtained sample size of at least 29 participants per group is adequate to detect a Cohen’s f of 0.34.

The sample reflected the diversity of the campus, with an ethnic composition of 31.52% Asian, 1.09% Black or African American, 21.74% Hispanic or Latino, 31.52% White, and 14.13% multiracial or other. Although the sample was recruited from a course with the same name as in Study 2, this course used a different textbook and was taught by a different instructor. As in the previous studies, students volunteered to participate in the study in exchange for extra credit and did not receive any other forms of compensation.

### Measures

**Pretest.** The pretest contained six questions designed to assess participants’ existing knowledge of normal probability distributions (see Appendix E). Four questions in the pretest were the same as in the pretest from Study 2. Two additional questions were added to ask students to further explain their answers and probe their thinking. The first five questions of the pretest were also included in the immediate posttest.

### Accuracy of Judgment of Learning. Similar to Study 2, participants’ accuracy of judgment of learning was calculated by the bias measure (Griffin et al., 2009; Maki et al. [2005](https://link.springer.com/article/10.1007/s12144-013-9198-x#ref-CR28)).Because the control group did not learn about the normal distribution, the judgment of learning measure was administered only to the Dynamic Slides group and Drawing+Hand group.

**Immediate posttest.** The immediate posttest contained 13 questions in total (See Appendix F). We revised the posttest based on how students answered the questions in Study 2, by removing questions that were ambiguous or too difficult. The questions were shown to students in the same fashion as before. Cronbach’s alpha for the 13 questions was .73.

### Scoring of Tests

Three trained coders, blind to each participant's experimental condition, scored 20 participants’ responses on the pretest and the immediate posttest independently from each other. For these 20 participants, each question was randomly assigned to be scored by two of the three coders. The Krippendorff’s Alpha was 0.92, which indicated good interrater reliability. The three coders then divided the responses into three sets and coded the rest of the responses independently, without knowing the participants’ condition assignments.

**Results**

***Pretest Performance***

The average accuracy of the pretest for all groups was 56% (3.38 out of 6 points). The control group scored 53% (SD = 0.28). The *Dynamic Slides* group scored 59% (SD = 0.30). The *Drawing+Hand* group scored 57% (SD = 0.26). Participants’ performance on the pretest did not differ significantly across groups (*F*(2,89) = .34, *p* = .712, η^2^ = .01).

### Accuracy of Judgment of Learning

Two participants were removed from this analysis because they did not provide a valid number for their self-rated understanding of the video. The distribution of participants’ accuracy of judgment of learning for the *Dynamic Slides* group and *Drawing+Hand* group is shown in Figure 10. The *Dynamic Slides* group and *Drawing+Hand* group did not differ significantly in their accuracy of judgment of learning (*t*_(60)_ = 0.77, *p* = .444).

### Figure 10

### Accuracy of Judgment of Learning by Condition. Conventions are the same as in Figure 6.


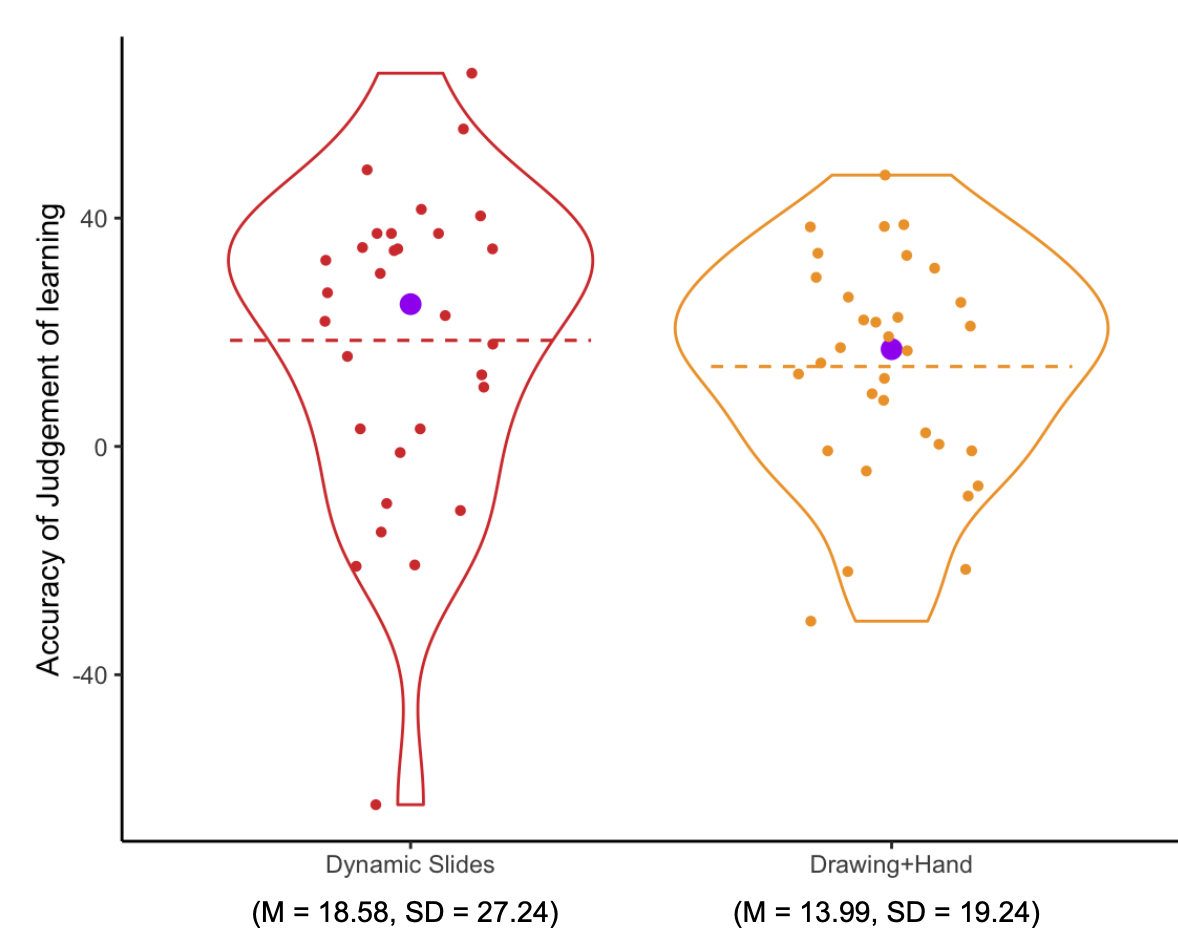


We also tested, using one sample t-tests, whether each group’s average judgments of learning were significantly different from 0. The *Dynamic Slides* group significantly overestimated their learning (*t*(29) = 3.74, *p* < .001), so did the *Drawing+Hand* group (*t*(31) = 4.11, *p* < .001).

### Immediate Posttest Performance

We conducted a one-way ANCOVA to evaluate the impact of condition on student’s posttest performance while controlling for their pretest performance by including it as a covariate. There was a significant group difference on the posttest (*F*_(2,88)_ = 4.48, *p* = .014, *η^2^* = .12) (Figure 11; Table 5).

**Table 5**

*ANCOVA results (Study 3)*

|  | *df* | *MS* | F | *η_p_^2^* | PRE | p |
| --- | --- | --- | --- | --- | --- | --- |
| Condition | 2 | 0.20 | 4.48 | .12 | .09 | .014 |
| Pretest Performance | 1 | 1.77 | 77.33 | .47 | .47 | <.001 |

##

Post hoc pairwise comparisons controlling for pretest performance revealed that students in the *Drawing+Hand* condition scored higher than those in the control condition (*t*_(88)_ = 4.86, *p_adj_* < .001), but did not differ significantly from those in the *Dynamic Slides* condition (*t*_(88)_ = 1,73, *p_adj_* = .264). The *Dynamic Slides* group also outperformed the control group (*t*_(88)_ = 3.08, *p_adj_* = .008). (Note: the *p*-values were adjusted for multiple comparisons using Bonferroni.)

**Figure 11**

*Violin plots showing posttest scores by condition. Conventions are the same as in Figure 6.*


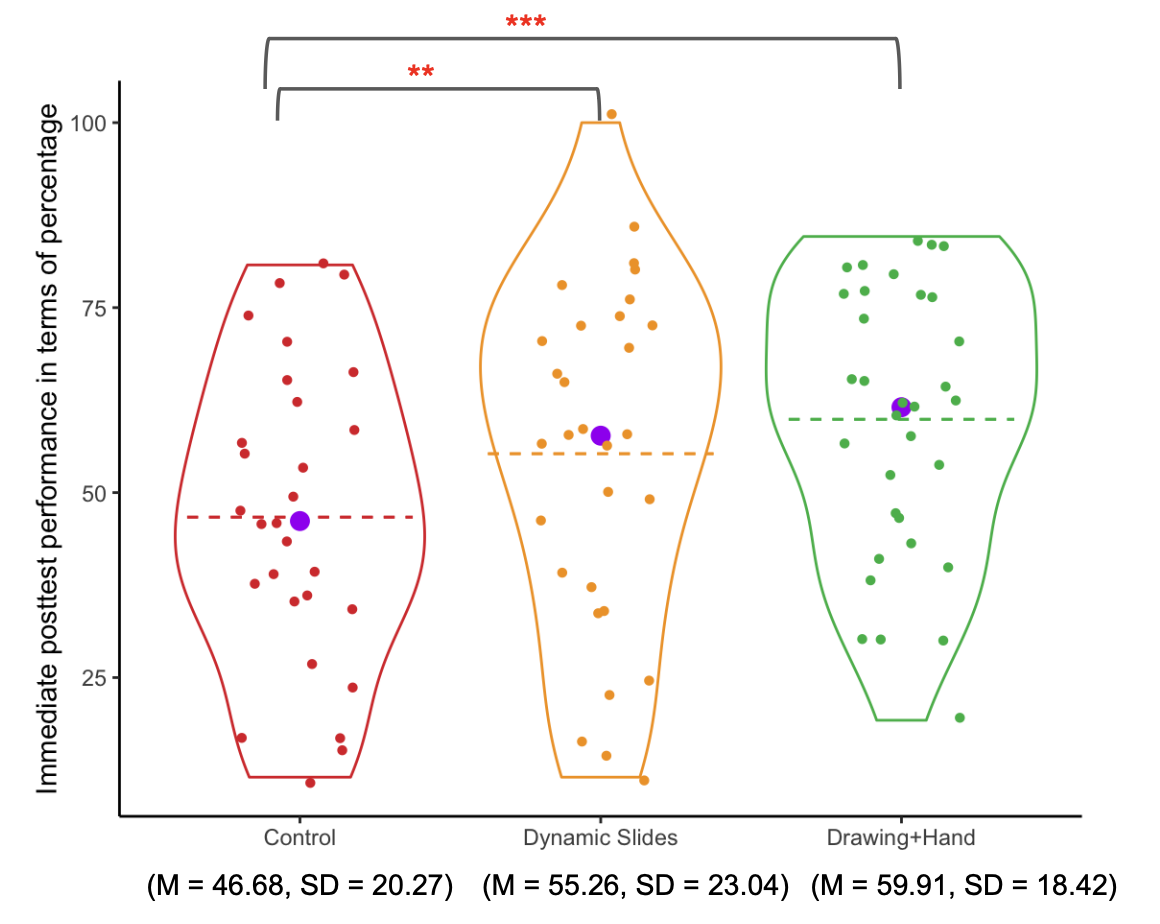


# Discussion

# In Study 3, both the *Drawing+Hand* and the *Dynamic Slides* groups exhibited superior learning outcomes compared to the control group, and did not significantly differ from one another. The findings from Study 3 did not provide sufficient evidence to support the notion that hand drawing with a visible hand possesses a unique advantage over dynamic slides with a moving cursor. This finding suggests that at least in this learning context, a learning benefit can be obtained with either dynamic drawings with a visible hand or through the use of dynamic slides, where the inclusion of a moving cursor and highlighting potentially serves to direct the learner’s focus.

# However, it is also possible that the moving cursor activated a representation of the hand. When people see a cursor moving in their everyday life, it’s moving because their hand is moving to control the cursor. Thus, in the *Dynamic Slides* condition, the effect of embodiment may have been through this pathway. Future studies might explore this hypothesis with a condition (e.g. highlighted dynamic slides) that is less likely to activate the representation of the hand.

# Moreover, Study 3 was only powered to detect large effects, so we cannot exclude smaller differences between embodiment and dynamic slides. If such differences exist, larger sample sizes and more sensitive measures may be required to sort out the importance of the drawing hand and, more generally, embodied representations.

**General Discussion**

Collectively, the three studies presented in this paper shed light on the efficacy of drawing in promoting higher-order cognitive processes. By comparing what is learned from watching a hand draw against other dynamic visualizations (e.g., no-hand drawings, computer-generated animations) and static drawings, these studies advance our understanding of the practical value of drawing during instruction. Study 2 points to the importance of the hand in helping learners reap the benefits of dynamic drawings. Study 3 points to the attentional role of drawings; a cursor moving around has similar benefits when compared to a dynamic hand drawing. It is worth noting that drawing controls the timing of the explanation. Because in both Study 2 and Study 3, the two experimental conditions shared the same audio track that was recorded when the instructor was drawing, the movement of the cursor is also controlled by the hand drawing. In addition to contributing to our basic understanding of the cognitive benefits of observing drawing, our findings also have a practical utility in demonstrating that misunderstandings of the normal distribution can be remedied using relatively brief videos delivered outside the flow of normal classroom activities.

Part of our motivation for these studies is to examine pedagogical techniques already employed by many instructors (drawings, computer-generated visualizations). Instructors often have intuitions about the importance of dynamic visualizations that unfold over time. Some instructors use computer-generated visualizations but reveal parts of them over time in a "step-by-step" manner, closely mimicking continuous drawing. How do these teaching materials compare to our experimental conditions in their efficacy? Given that the drawing scenario is harder and less clean to implement in the classroom, teachers often face the trade-offs between ease of implementation and student improvement. Study 2 and Study 3 provided some initial clues into this challenge, but future research could future delineate ideal trade-offs.

## Limitations and future directions

There are some limitations that the current three studies could not address. First, we note that our specific sampling method, although allowing researchers to examine students’ knowledge after a certain amount of instruction, has several implications. First, the samples for these studies consisted of mostly psychology students from a specific institution. Misunderstandings regarding probability distributions might be different in other populations, such as community college students; they may be either more severe/prevalent, if students already arrive at statistics classes with lower preparation, or less severe, if instructors dedicate more time to cover these topics or can address more individual misunderstanding in smaller classes. Moreover, even if the level and forms of misunderstanding prove to be similar across diverse populations, we cannot at present determine whether the intervention used here would prove as effective for such diverse groups of students. Future studies should explore these questions. Lastly to this first major aspect of having opportunistic sampling by using extra credit to recruit students, we caution other researchers pursuing similar methods of recruitment to make sure the experience of participating in this research provides educational benefits and students are provided with other options of gaining the extra credit if they opt out.

Another aspect of the limitations is that, due to constraints imposed by the COVID-19 pandemic, many students in our sample did not have a disturbance-free workspace for watching the videos (e.g., they reported being in a noisy household where they could not hear the audio clearly). For this reason, we excluded students based on their self-reported level of disturbance, but this exclusion criterion might have biased our results in some way (e.g., causing excessive exclusion of students from a particular socioeconomic status that may be correlated with both less privacy in one’s household and lower college preparation). Although future researchers might learn much by replicating our approach in a more controlled environment, it is also interesting to understand how the natural variation that occurs across students’ remote learning environments might impact their learning. Indeed, future interventions should be designed to fit the circumstances in which they are experienced by students.

**Conclusion**

This paper reports on a set of three studies that identified students’ current struggles with the normal probability distribution and investigated the effectiveness of drawings and other visualizations as scaffolding tools to help students better understand that topic. This research offers valuable insights into the practical application of drawing as a cognitive tool: to put it in the simplest way, dynamic drawing with a visible hand produced better learning outcomes than both static slides and dynamic drawing without a visible hand, but not significantly different from dynamic slides (i.e., a cursor moving around otherwise static slides). The findings suggest drawing is a potent tool in multimedia learning but its benefit might be achieved similarly by dynamic slides, through which learners are engaged by animation and highlighting. These findings have important implications for instructional practices, emphasizing the relevance of incorporating drawing as a powerful tool to facilitate comprehension and enhance learning outcomes.

**Open Practice Statement**

The study was not pre-registered. All video materials can be accessed through the OSF wiki page: <https://osf.io/af3p9/?view_only=e0668f936b584577b2b5ffacb66d6d2f>. Datasets are available upon request.

# References

Adams, D. M., McLaren, B. M., Durkin, K., Mayer, R. E., Rittle-Johnson, B., Isotani, S., & van Velsen, M. (2014). Using erroneous examples to improve mathematics learning with a web-based tutoring system. *Computers in Human Behavior, 36,* 401–411. [https://doi.org/
10.1016/j.chb.2014.03.053](https://psycnet.apa.org/doi/10.1016/j.chb.2014.03.053)

Ainsworth, S. (2006). Deft: a conceptual framework for considering learning with multiple representations. *Learning and Instruction*, *16*(3), 183–198.

Ainsworth, S. (2008). The educational value of multiple-representations when learning complex scientific concepts. In *Visualization: Theory and practice in science education* (pp. 191-208). Springer, Dordrecht.<http://dx.doi.org/10.1007/978>

Ainsworth, S. E., & Scheiter, K. (2021). Learning by drawing visual representations: Potential, purposes, and practical implications. *Current Directions in Psychological Science*, *30*(1), 61-67.

Airey, J., & Linder, C. (2009). A disciplinary discourse perspective on university science learning: Achieving fluency in a critical constellation of modes. *Journal of Research in Science Teaching, 46*(1), 27–49.<http://dx.doi.org/10.1002/tea.20265>

Ayres, P., & Paas, F. (2007). A cognitive load approach to the learning effectiveness of instructional animation. *Applied Cognitive Psychology*, *21*(6).

Ayres, P., Marcus, N., Chan, C., & Qian, N. (2009). Learning hand manipulative tasks: When instructional animations are superior to equivalent static representations. *Computers in Human Behavior, 25*, 348–353.<https://doi.org/10.1016/j.chb.2008.12.013>

Bandura, A. (1986). Fearful expectations and avoidant actions as coeffects of perceived self-inefficacy.

Batanero, C., Tauber, L. M., & Sánchez, V. (2004). Students’ reasoning about the normal distribution. In D. Ben-Zvi & J. Garfield (Eds.), *The challenge of developing statistical literacy, reasoning and thinking* (pp. 257-276). Springer, Dordrecht.

Bartram, D. J. (1980). Comprehending spatial information: The relative efficiency of different methods of presenting information about bus routes. *Journal of Applied Psychology*, *65*(1), 103.

Blake A., Chrabaszcz, J., Son, J.Y., & Stigler J.W. (2023). supernova: Judd, McClelland, & Ryan Formatting for ANOVA Output. R package version 2.5.8, <https://github.com/UCLATALL/supernova>.

Bauer, M. I., & Johnson-Laird, P. N. (1993). How diagrams can improve reasoning. *Psychological science*, *4*(6), 372-378.

Carney, R. N., & Levin, J. R. (2002). Pictorial illustrations still improve students' learning from text. *Educational psychology review*, *14*, 5-26.

Castro-Alonso, J. C., Ayres, P., & Paas, F. (2014). Dynamic visualisations and motor skills. In *Handbook of human centric visualization* (pp. 551-580). Springer, New York, NY. http://dx.doi.org/10.1007/978-1-4614-7485-2_22

Castro-Alonso, J. C., Ayres, P., & Paas, F. (2015). Animations showing Lego manipulative tasks: Three potential moderators of effectiveness. *Computers & Education*, *85*, 1-13. https://doi.org/10.1016/j.compedu.2014.12.022

Chance, B., del Mas, R., & Garfield, J. (2004). Reasoning about sampling distributions. In D. Ben-Zvi & J. Garfield (Eds.), *The challenge of developing statistical literacy, reasoning and thinking* (pp. 295-323). Springer, Dordrecht.

Chandler, P. (2004). The crucial role of cognitive processes in the design of dynamic visualizations. *Learning and Instruction*, *14*(3), 353-357.<http://dx.doi.org/10.1016/> [j.learninstruc.2004.06.009](http://dx.doi.org/10.1016/j.learninstruc.2004.06.009)

Clark, J., & Paivio, A. (1991). Dual coding theory and education. *Educational Psychology Review, 3*(3), 149–210. <https://doi.org/10.1007/bf01320076>.

Cohen, S., & Chechile, R. A. (1997). Probability distributions, assessment, and instructional software: Lessons learned from an evaluation of curricular software. In I. Gal & J. B. Garfield (Eds.), *The assessment challenge in statistics education* (pp. 253-262). IOS Press.

Cromley, J. G., Du, Y., & Dane, A. P. (2020). Drawing-to-learn: does meta-analysis show differences between technology-based drawing and paper-and-pencil drawing?. *Journal of Science Education and Technology*, *29*, 216-229.

Da Rold, F. (2018). Defining embodied cognition: The problem of situatedness. *New Ideas in Psychology*, *51*, 9-14.

de Koning, B. B., & Tabbers, H. K. (2011). Facilitating understanding of movements in dynamic visualizations: An embodied perspective. *Educational Psychology Review*, *23*(4), 501-521.<http://dx.doi.org/10.1007/s10648-011-9173-8>

de Koning, B. B., & Tabbers, H. K. (2013). Gestures in instructional animations: A helping hand to understanding non‐human movements?. *Applied Cognitive Psychology*, *27*(5), 683-689.<https://doi.org/10.1002/acp.2937>

Fiorella, L., & Mayer, R. E. (2016). Effects of observing the instructor draw diagrams on learning from multimedia messages. *Journal of Educational Psychology, 108*(4), 528–546.<https://doi.org/10.1037/edu0000065>

Fiorella, L., & Zhang, Q. (2018). Drawing boundary conditions for learning by drawing. *Educational Psychology Review, 30*(3), 1115–1137. [https://doi.org/10.1007/s10648-
018-9444-8](https://psycnet.apa.org/doi/10.1007/s10648-018-9444-8)

Fiorella, L., Stull, A. T., Kuhlmann, S., & Mayer, R. E. (2019). Instructor presence in video lectures: The role of dynamic drawings, eye contact, and instructor visibility. *Journal of Educational Psychology, 111*(7), 1162–1171.<https://doi.org/10.1037/edu0000325>

Fu, Y., & Franz, E. A. (2014). Viewer perspective in the mirroring of actions. *Experimental brain research*, *232*, 3665-3674.

Fuad, M. M., & Jones, E. J. (2012). Using extra credit to facilitate extra learning in students. *International Journal of Modern Education and Computer Science*, *4*(6), 35.

García, A. M., Hesse, E., Birba, A., Adolfi, F., Mikulan, E., Caro, M. M., ... & Ibáñez, A. (2020). Time to face language: embodied mechanisms underpin the inception of face-related meanings in the human brain. *Cerebral Cortex*, *30*(11), 6051-6068.

Gilbert, J. K. (2005). Visualization: A metacognitive skill in science and science education. In *Visualization in science education* (pp. 9-27). Dordrecht: Springer Netherlands.

Gilbert, J. K., Reiner, M., & Nakhleh, M. (Eds.). (2007). *Visualization: Theory and practice in science education* (Vol. 3). Springer Science & Business Media.

Glenberg, A. M., Goldberg, A. B., & Zhu, X. (2011). Improving early reading comprehension using embodied CAI. *Instructional Science, 39*(1), 27-39.<https://doi.org/10.1007/s11251-> [009-9096-7](https://doi.org/10.1007/s11251-009-9096-7)

Glenberg, A. M., Sato, M., Cattaneo, L., Riggio, L., Palumbo, D., & Buccino, G. (2008). Processing abstract language modulates motor system activity. *Quarterly Journal of Experimental Psychology*, *61*(6), 905-919.<https://doi.org/10.1080/17470210701625550>

Halpern, D. F., & Hakel, M. D. (2002). Learning that lasts a lifetime: teaching for long‐term retention and transfer. *New Directions for Teaching and Learning*, *2002*(89), 3-7.

Hegarty, M. (2004). Dynamic visualizations and learning: Getting to the difficult questions. *Learning and Instruction*, *14*(3), 343-351.<https://doi.org/10.1016/j.learninstruc>[.
2004.06.007](https://doi.org/10.1016/j.learninstruc.2004.06.007)

Höffler, T. N.， & Leutner, D. (2007). Instructional animation versus static pictures: A meta-analysis. *Learning and Instruction, 17*(6), 722-738.<https://doi.org/10.1016/j>[.
Learninstruc.2007.09.013](https://doi.org/10.1016/j.learninstruc.2007.09.013)

Holliday, W. G. (1977). Differential cognitive and affective responses to flow diagrams in science. *Journal of Research in Science Teaching*, *14*(2), 129-138.

Hosler, J., Boomer, K. B., & Kalumuck, K. (2011). Are comic books an effective way to engage nonmajors in learning and appreciating science? *CBE Life Sciences Education, 10*(3), 309–317. <https://doi.org/10.1187/cbe.10-07-0090>.

Hsu, J. (1996). *Multiple comparisons: theory and methods*. CRC Press.

Holliday, W. G. (1977). Differential cognitive and affective responses to flow diagrams in science. *Journal of Research in Science Teaching*, *14*(2), 129-138.

Lowe, R. (1999). Extracting information from an animation during complex visual learning. *European journal of psychology of education*, *14*(2), 225-244.<https://doi.org/10.1007/> [BF03172967](https://doi.org/10.1007/BF03172967)

Lowe, R. (2004). Interrogation of a dynamic visualization during learning. *Learning and Instruction, 14*(3), 257–274.<https://doi.org/10.1016/j.learninstruc.2004.06.003>

Maxwell, S. E., Delaney, H. D., & Kelley, K. (2017). *Designing experiments and analyzing data: A model comparison perspective*. Routledge.

Mayer, R., & DaPra, C. S. (2012). An embodiment effect in computer-based learning with animated pedagogical agents. *Journal of Experimental Psychology: Applied, 18*(3), 239–252. [https://doi.org/10.1037/a0028616](https://psycnet.apa.org/doi/10.1037/a0028616)

Mayer, R., & Moreno, R. (2002). Animation as an aid to multimedia learning. *Educational Psychology Review, 14*(1), 87–99. [https://doi.org/10.1023/A:1013184611077](https://psycnet.apa.org/doi/10.1023/A:1013184611077)

Mayer, R. (2014). Principles based on social cues in multimedia learning: Personalization, voice, image, and embodiment principles. *The Cambridge handbook of multimedia learning*, *16*, 345-370.

Mayer, R. E. (Ed.). (2005). *The Cambridge handbook of multimedia learning*. Cambridge university press.

McElhaney, K. W., Chang, H. Y., Chiu, J. L., & Linn, M. C. (2015). Evidence for effective uses of dynamic visualisations in science curriculum materials. *Studies in Science Education, 51*(1), 49–85.<http://dx.doi.org/10.1080/03057267.2014.984506>

McLaren, B. M., Adams, D. M., & Mayer, R. E. (2015). Delayed learning effects with erroneous examples: a study of learning decimals with a web-based tutor. *International Journal of Artificial Intelligence in Education, 25*(4), 520-542.<https://doi.org/10.1007/s40593-> [015-0064-x](https://doi.org/10.1007/s40593-015-0064-x)

Miller, T. M., & Geraci, L. (2011). Unskilled but aware: reinterpreting overconfidence in low-performing students. *Journal of experimental psychology: learning, memory, and cognition*, *37*(2), 502.

Moreno, R., Mayer, R. E., Spires, H. A., & Lester, J. C. (2001). The case for social agency in computer-based teaching: Do students learn more deeply when they interact with animated pedagogical agents?. *Cognition and instruction, 19*(2), 177-213.

Moreno, R., Reislein, M., & Ozogul, G. (2010). Using virtual peers to guide visual attention during learning: A test of the persona hypothesis. *Journal of Media Psychology: Theories, Methods, and Applications, 22*(2), 52–60. [https://doi.org/10.1027/1864-1105/
a000008](https://psycnet.apa.org/doi/10.1027/1864-1105/a000008)

Quillin, K., & Thomas, S. (2015). Drawing-to-learn: a framework for using drawings to promote model-based reasoning in biology. *CBE—Life Sciences Education*, *14*(1), es2.

Rau, M. A. (2017). How do Students Learn to See Concepts in Visualizations? Social Learning Mechanisms with Physical and Virtual Representations. *Journal of Learning Analytics*, *4*(2), 240–263.<http://dx.doi.org/10.18608/jla.2017.42.16>

Rieber, L. P. (1991). Animation, incidental learning and continuing motivation. *Journal of Educational Psychology, 83*, 318–328.<https://doi.org/10.1037/0022-0663.83.3.318>

Risko, E. F., & Gilbert, S. J. (2016). Cognitive offloading. *Trends in cognitive sciences*, *20*(9), 676-688.

Rother, M. (2009). *Toyota kata*. New York, NY: McGraw-Hill Professional Publishing.

Rowland, C. A. (2014). The effect of testing versus restudy on retention: a meta-analytic review of the testing effect. *Psychological bulletin*, *140*(6), 1432.

Rueckert, L., Church, R. B., Avila, A., & Trejo, T. (2017). Gesture enhances learning of a complex statistical concept. *Cognitive Research: Principles and Implications*, *2*(1), 1-6. <http://dx.doi.org/10.1186/s41235-016-0036-1>

Scanlan, D. A. (1989). Structured flowcharts outperform pseudocode: An experimental comparison. *IEEE software*, *6*(5), 28-36.

Schleinschok, K., Eitel, A., & Scheiter, K. (2017). Do drawing tasks improve monitoring and control during learning from text?. *Learning and Instruction*, *51*, 10-25.

Schmeck, A., Mayer, R. E., Opfermann, M., Pfeiffer, V., & Leutner, D. (2014). Drawing pictures during learning from scientific text: Testing the generative drawing effect and the prognostic drawing effect. *Contemporary Educational Psychology*, *39*(4), 275-286.

Schmidgall, S. P., Eitel, A., & Scheiter, K. (2019). Why do learners who draw perform well? Investigating the role of visualization, generation and externalization in learner-generated drawing. *Learning and Instruction*, *60*, 138-153.

Schuil, K. D., Smits, M., & Zwaan, R. A. (2013). Sentential context modulates the involvement of the motor cortex in action language processing: An fMRI study. *Frontiers in human neuroscience*, *7*, 100.

Sepp, S., Howard, S. J., Tindall-Ford, S., Agostinho, S., & Paas, F. (2019). Cognitive Load Theory and Human Movement: Towards an Integrated Model of Working Memory. *Educational Psychology Review, 31*(2), 293-317. <https://doi.org/10.1007/s10648-019-09461-9>

Shepard, R. N. (1967). Recognition memory for words, sentences, and pictures. *Journal of Verbal Learning and Verbal Behavior, 6*(1), 156–163. <https://doi.org/10.1016/s0022-5371(67)80067-7>.

Son, J. Y., Ramos, P., DeWolf, M., Loftus, W., & Stigler, J. W. (2018). Exploring the practicing-connections hypothesis: Using gesture to support coordination of ideas in understanding a complex statistical concept. *Cognitive Research: Principles and Implications*, *3*(1), 1-13.<http://dx.doi.org/10.1186/s41235-017-0085-0>

Stigler, J. W., Son, J. Y., Givvin, K. B., Blake, A., Fries, L., Shaw, S. T., & Tucker, M. C. (2020). The Better Book approach for education research and development. *Teachers College Record*, *123*(2), 1-32.

Suthers, D. D. (2014). Empirical studies of the value of con- ceptually explicit notations in collaborative learning. In A. Okada, S. J. Buckingham Shum, & T. Sherborne (Eds.), *Knowledge cartography: Software tools and mapping tech- niques* (pp. 1–22). Springer. https://doi.org/10.1007/978- 1-4471-6470-8_1

Sweller, J., Ayres, P., Kalyuga, S., Sweller, J., Ayres, P., & Kalyuga, S. (2011). Measuring cognitive load. *Cognitive load theory*, 71-85.

Thiede, K. W., & Dunlosky, J. (1999). Toward a general model of self-regulated study: An analysis of selection of items for study and self-paced study time. *Journal of experimental psychology: Learning, Memory, and Cognition*, *25*(4), 1024.

Thomas, L. E., & Lleras, A. (2009). Swinging into thought: Directed movement guides insight in problem solving. *Psychonomic Bulletin & Review*, *16*(4), 719-723. <http://dx.doi.org/10>.
3758/PBR.16.4.719

Tran, C., Smith, B., & Buschkuehl, M. (2017). Support of mathematical thinking through embodied cognition: Nondigital and digital approaches. *Cognitive research: principles and implications*, *2*, 1-18.

Tversky, B., Morrison, J. B., & Betrancourt, M. (2002). Animation: can it facilitate?. *International journal of human-computer studies*, *57*(4), 247-262.

Uttal, D. H., & O’Doherty, K. (2008). Comprehending and learning from ‘visualizations’: A developmental perspective. In J. Gilbert (Ed.), *Visualization: Theory and practice in science education* (pp. 53–72). Netherlands: Springer.<http://dx.doi.org/10.1007/978-> [1-4020-5267-5_3](http://dx.doi.org/10.1007/978-1-4020-5267-5_3)

Van Gog, T., Paas, F., Marcus, N., Ayres, P., & Sweller, J. (2009). The mirror neuron system and observational learning: Implications for the effectiveness of dynamic visualizations. *Educational Psychology Review*, *21*(1), 21-30.

van Meter, P., & Firetto, C. M. (2013). Cognitive model of drawing construction. *Learning through visual displays*, 247-280.

van Meter, P., & Garner, J. (2005). The promise and practice of learner-generated drawing: Literature review and synthesis. *Educational psychology review*, *17*, 285-325.

Vekiri, I. (2002). What is the value of graphical displays in learning?. *Educational psychology review*, *14*, 261-312.

Wilson, M. (2002). Six views of embodied cognition. *Psychonomic bulletin & review*, *9*, 625-636.

Wong, A., Marcus, N., Ayres, P., Smith, L., Cooper, G. A., Paas, F., & Sweller, J. (2009). Instructional animations can be superior to statics when learning human motor skills. *Computers in Human Behavior*, *25*(2), 339-347.<https://doi.org/10.1016/j.chb.2008.12>[.
012](https://psycnet.apa.org/doi/10.1016/j.chb.2008.12.012)

Wu, S. P., & Rau, M. A. (2019). How students learn content in science, technology, engineering, and mathematics (STEM) through drawing activities. *Educational Psychology Review*, *31*, 87-120.

Zhang, I., Givvin, K. B., Sipple, J. M., Son, J. Y., & Stigler, J. W. (2021). Instructed hand movements affect students’ learning of an abstract concept from video. *Cognitive Science, 45*(2), e12940.<https://doi.org/10.1111/cogs.12940>

Zhang, Z. H., & Linn, M. C. (2011). Can generating representations enhance learning with dynamic visualizations?. *Journal of research in science teaching*, *48*(10), 1177-1198.

#

# Appendix A: Study 1 Survey

Please get a blank piece of paper (at least letter size, 8.5 x 11 inches). Draw 5 horizontal lines to separate the entire piece of paper into 6 approximately equal parts and name each section A, B, C... (as below). If you feel like writing down some notes/drawing at any point, please use the back of the paper as a scratch paper and write down the question number for each note/drawing.


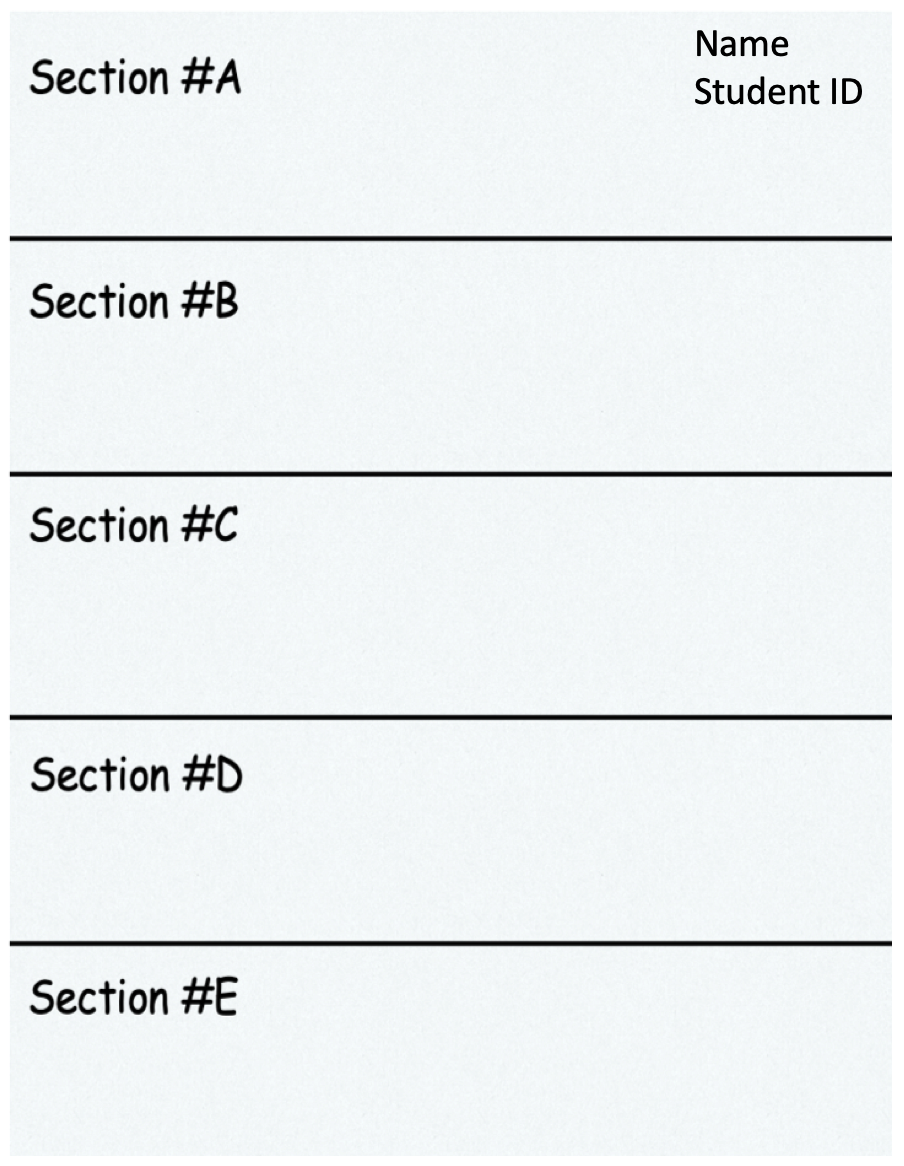


1. This is a normal probability distribution. Could you estimate the probability represented by the shaded region under the curve? If yes, what would your estimate be and why? If no, why not?


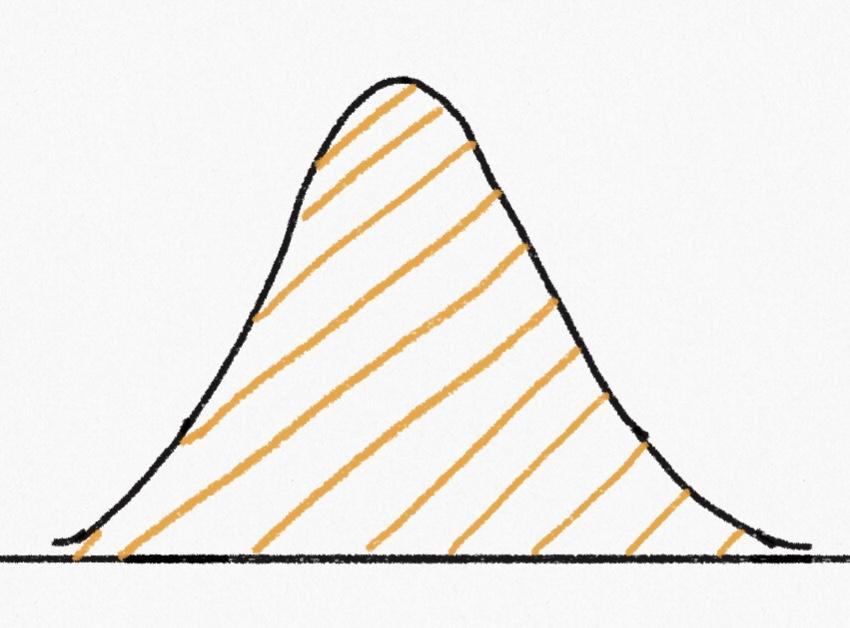


1. If we randomly select a single data point from the distribution, what’s the probability of the point falling somewhere in the shaded region under the normal curve? Explain your answer.


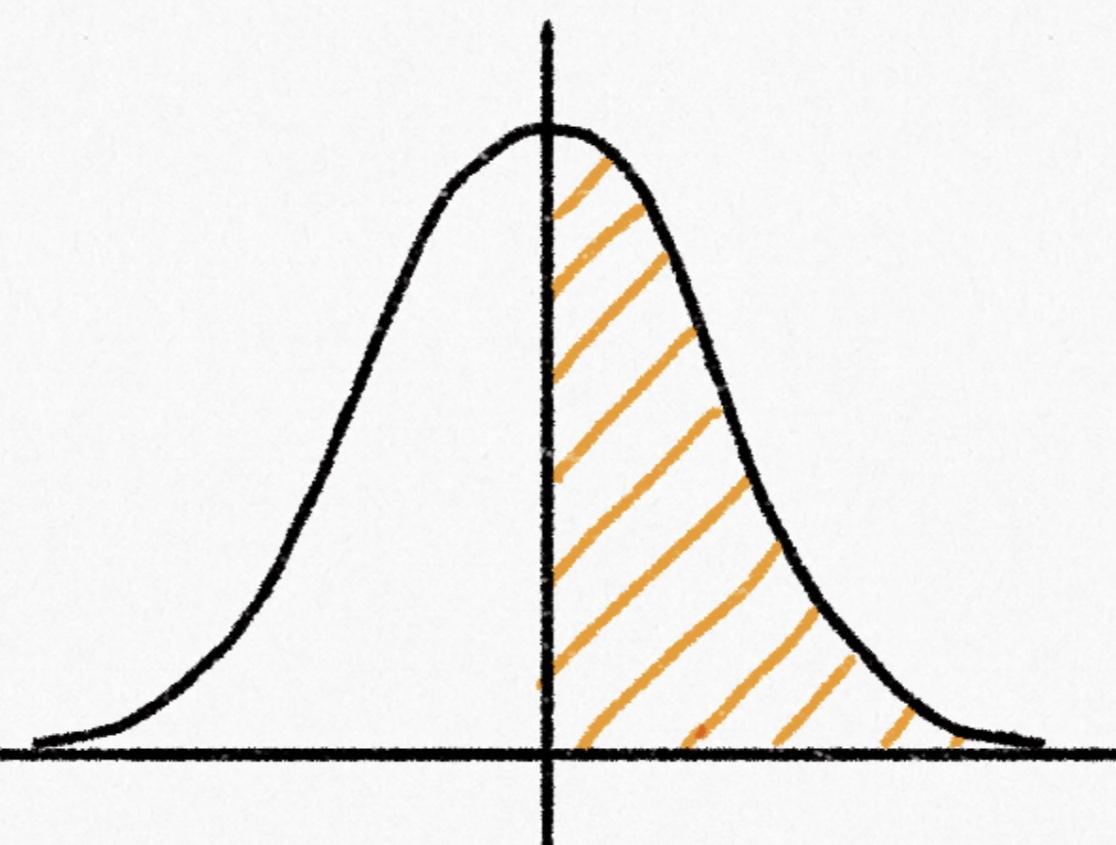


1. If we draw a normal distribution that is wider than the one in Question 1 (as below), how would the probability represented by the shaded part under the distribution change? Explain.


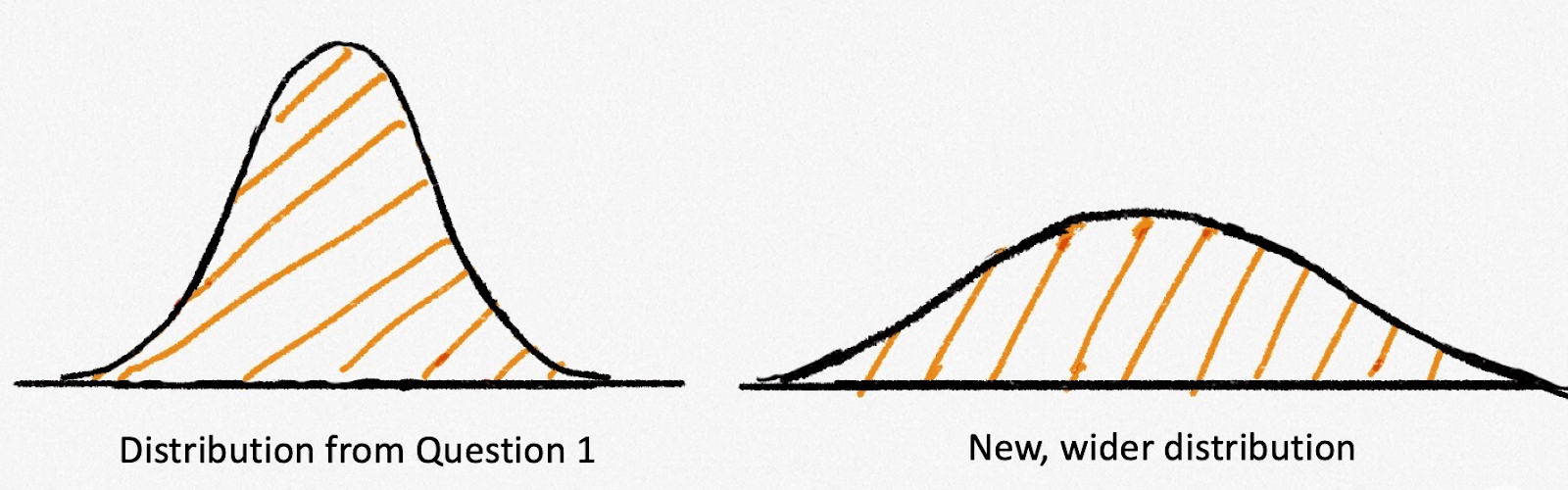


1. Draw a normal distribution in Section #A of the paper you’ve prepared. Draw a solid line to represent where a z score of 1 would fall in this normal distribution. Then, draw a dashed line to represent where a z score of -2 would fall on the same graph.
2. Here’s a drawing of a normally shaped population distribution with a mean of 8. The probability of a randomly sampled data point being **greater than** 10 is 0.2. Based on this, what is the probability of a randomly sampled data point being **greater than** 6? Explain your answer.


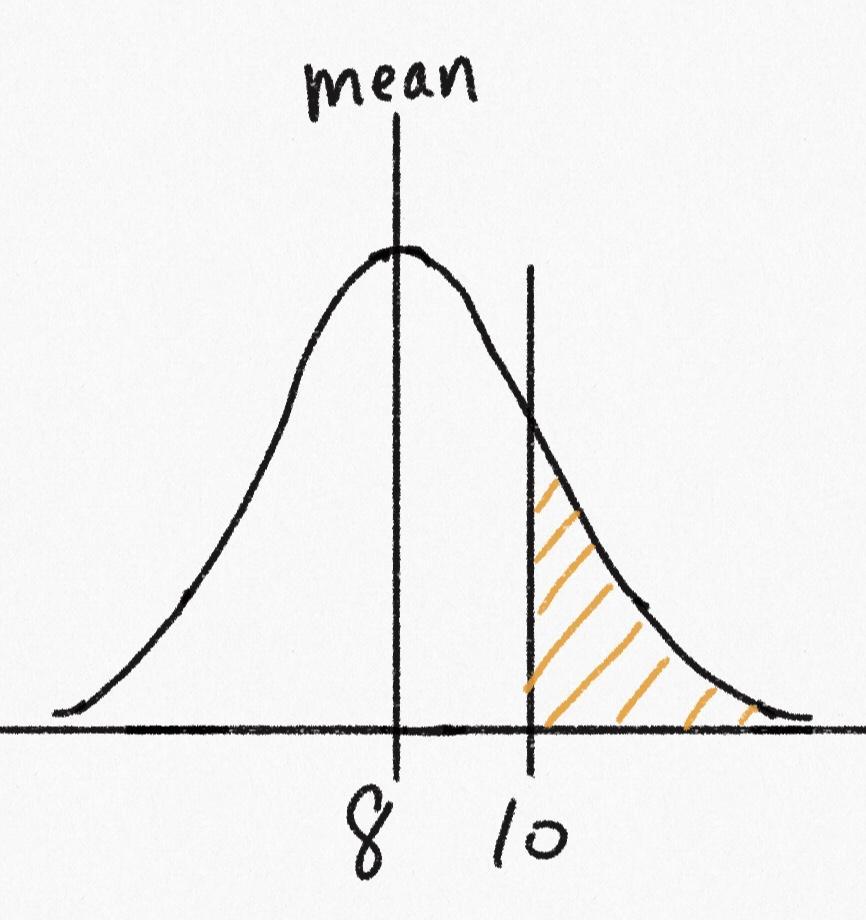


1. Based on what you know from the previous question, if you take a random sample of 30 data points, would the probability of getting a sample mean greater than 10 be greater than 0.2, lower than 0.2 or equal to 0.2? Explain your answer.
2. The figure below depicts the sampling distribution of the mean difference between two groups of an experiment under an empty model. Copy the graph below into Section #B of the paper you’ve prepared.
   1. Where would the center of the distribution be?
   2. Now, draw a line to represent the significance level, alpha = 0.05 (the line does not need to be precise) on the same graph and shade in the rejection region.


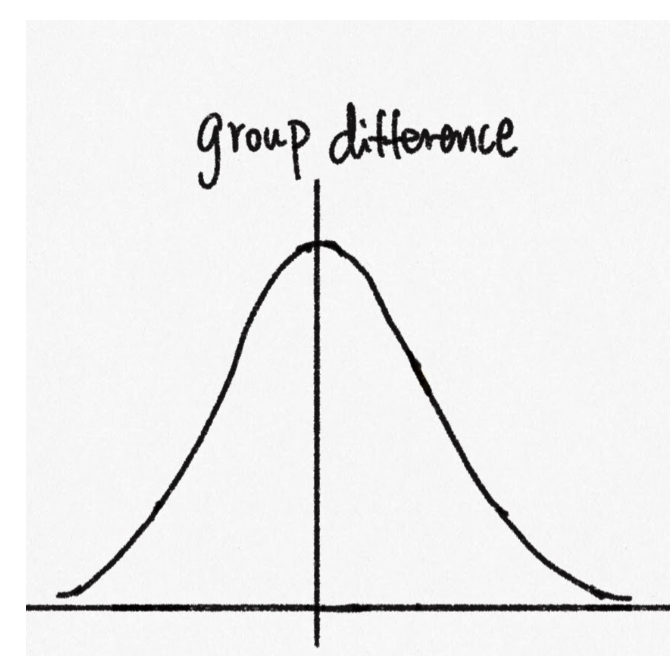


1. If we change the alpha level from 0.05 to 0.1, how would the area of the shaded region from the previous question change? Represent the change in Section #C of the paper you’ve prepared and explain your answer.
2. If we change the alpha level from 0.05 to 0.1, would it increase or decrease the likelihood of rejecting the null model (i.e. the null hypothesis)? Explain your answer.
3. If you conducted an experiment and observed a group difference with a p value of 0.25, what would you conclude about the null model? Explain your answer.
4. Based on the drawing below, which would have a larger p value? The probability of getting a difference between the two groups larger than 3.1 or the probability of getting a difference between the two groups smaller than -3.3? Explain your answer.


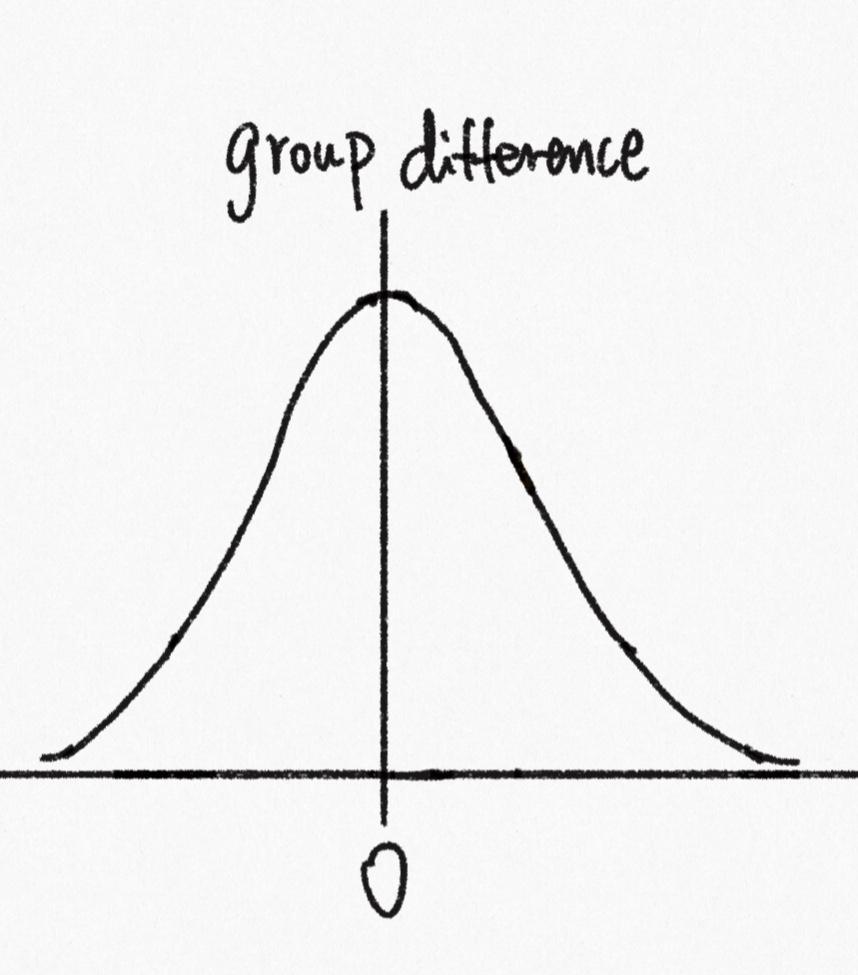


1. Imagine that you conducted a study to compare an outcome between two groups. You rejected the empty model with a p = 0.02. How could you compute the probability that you would again reject the empty model if you replicated the study? Explain your answer. Feel free to draw graphs on the back of the paper you’ve prepared to illustrate your answer.
2. The shaded area in the graph below represents the statistical power of this hypothesis test. Based on the graph and your previous knowledge, explain what you think statistical power is.


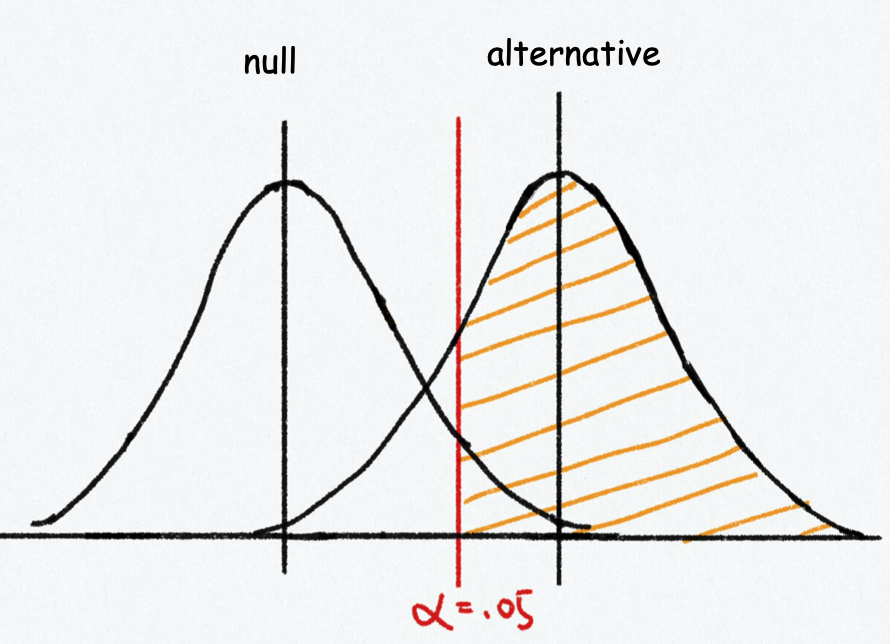


1. Type II error is failing to reject the null when the alternative is in fact true. Copy the drawing below into Section #E of your paper. On the graph, shade the area that represents the probability of making a Type II error and explain your drawing.
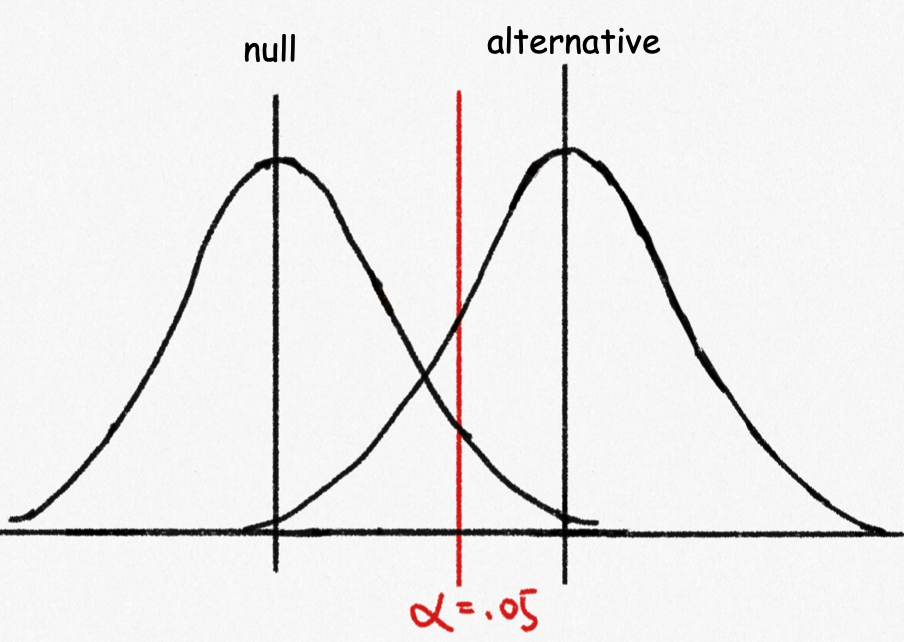

2. If the variability of the sample increases, what do you think would happen to statistical power? Feel free to draw (in Section #F of the paper) and use words to explain.

Appendix B: Study 2 Pretest

1. This is a normal probability distribution. Could you estimate the probability represented by the shaded region under the curve? If yes, what would your estimate be and why? If no, why not?


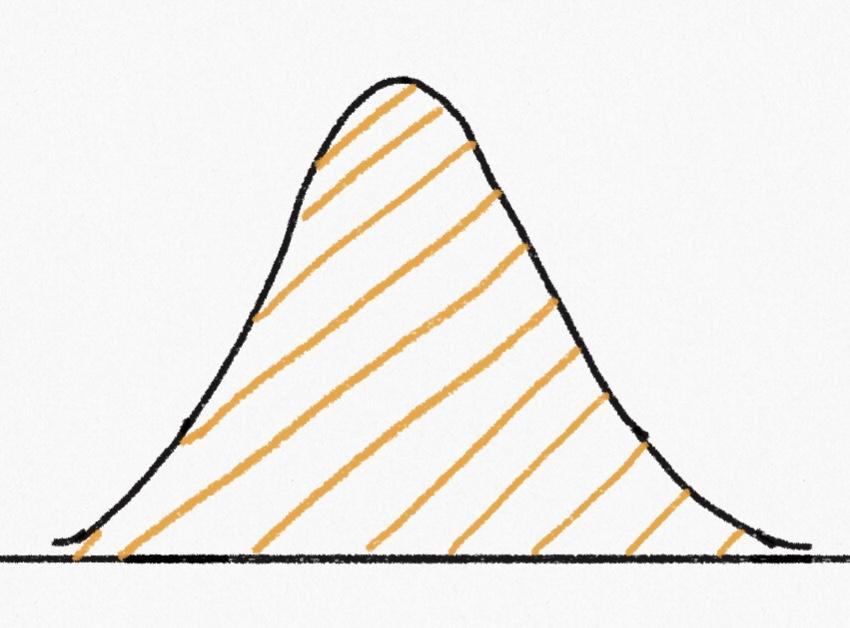


1. Which of the following are equal to 1? (Choose all that apply)
   1. The sum of the heights of all bars in a density histogram
   2. The sum of the areas of all bars in a density histogram
   3. The sum of the heights of all bars in a frequency histogram
   4. The sum of the areas of all bars in a frequency histogram
   5. The area under a normal probability curve
   6. The area under any probability curve
2. What does the area of a bar in a density histogram represent?
3. If we randomly select a single data point from the distribution, what’s the probability of the point falling somewhere in the shaded region under the normal curve? Explain your answer.


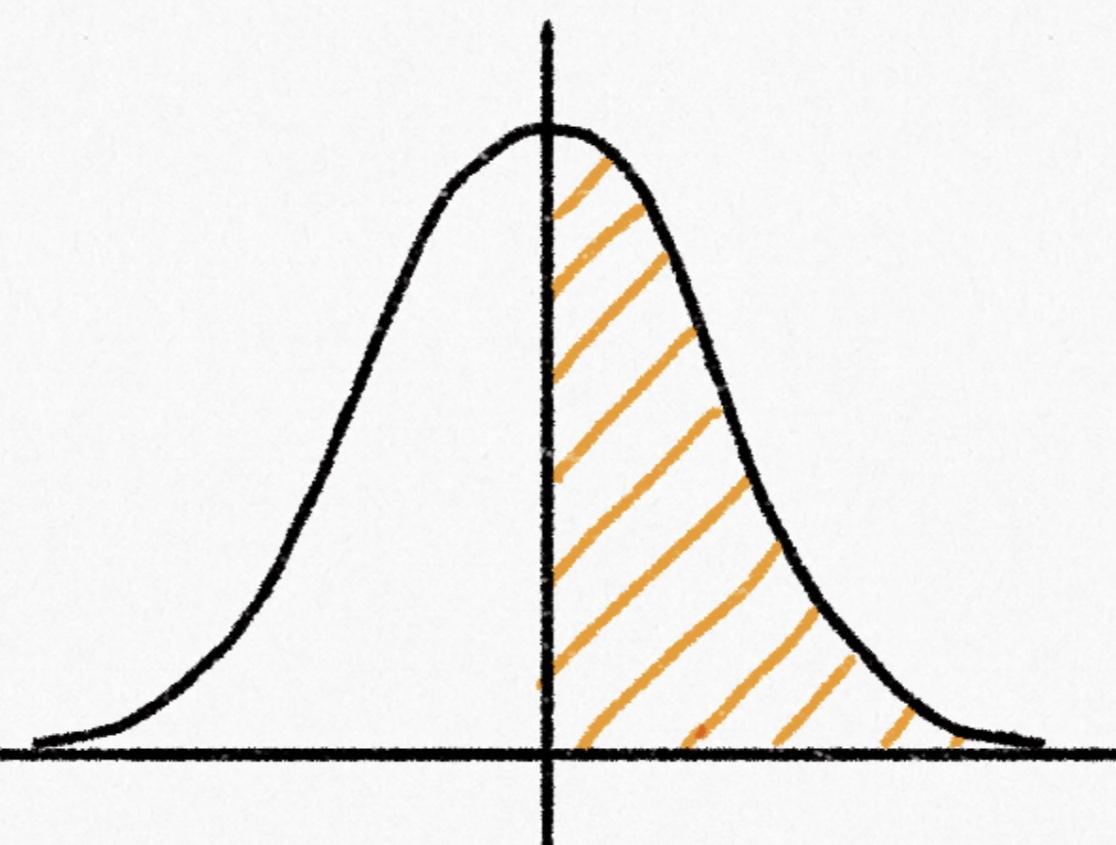


1. If we draw a normal distribution that is wider than the one in Question 1 (as below). How would the probability represented by the shaded part under the distribution change?


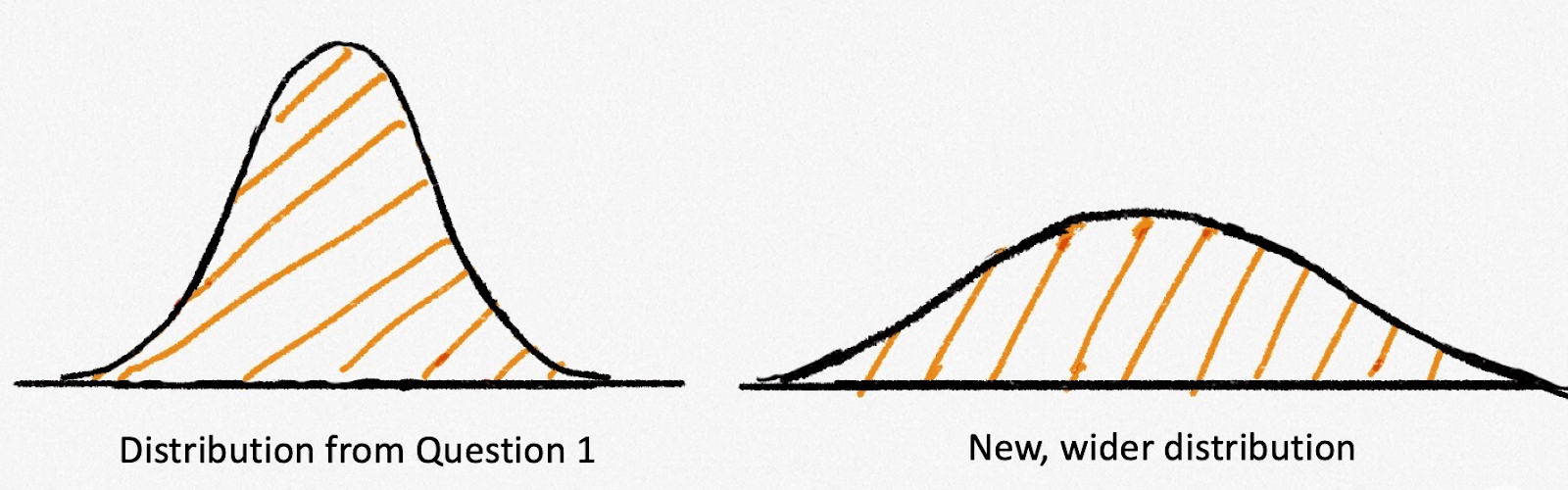


- 1. increase
  2. decrease
  3. stay the same
  4. impossible to tell

1. Explain your answer to the previous question:
2. Here’s a drawing of a normally shaped population distribution with a mean of 8. The probability of a randomly sampled data point being **greater than** 10 is 0.25. Based on this, what is the probability of a randomly sampled data point being **greater than** 6? Explain your answer.


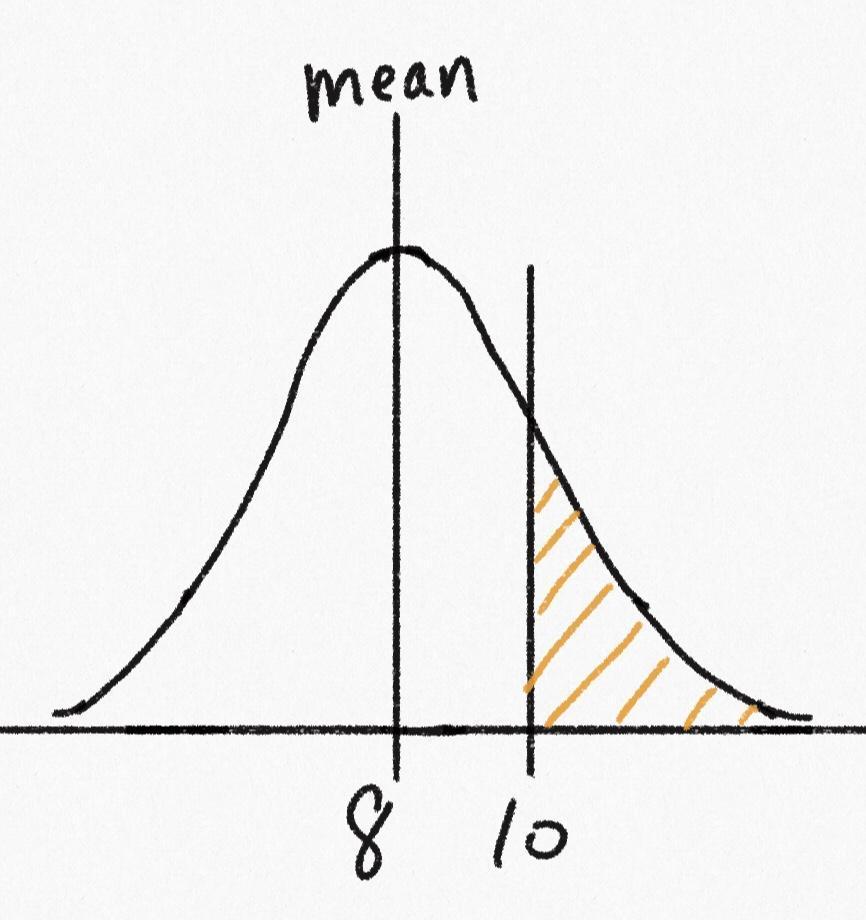


Appendix C: Study 2 immediate posttest

1. This is a normal probability distribution. Could you estimate the probability represented by the shaded region under the curve? If yes, what would your estimate be and why? If no, why not?


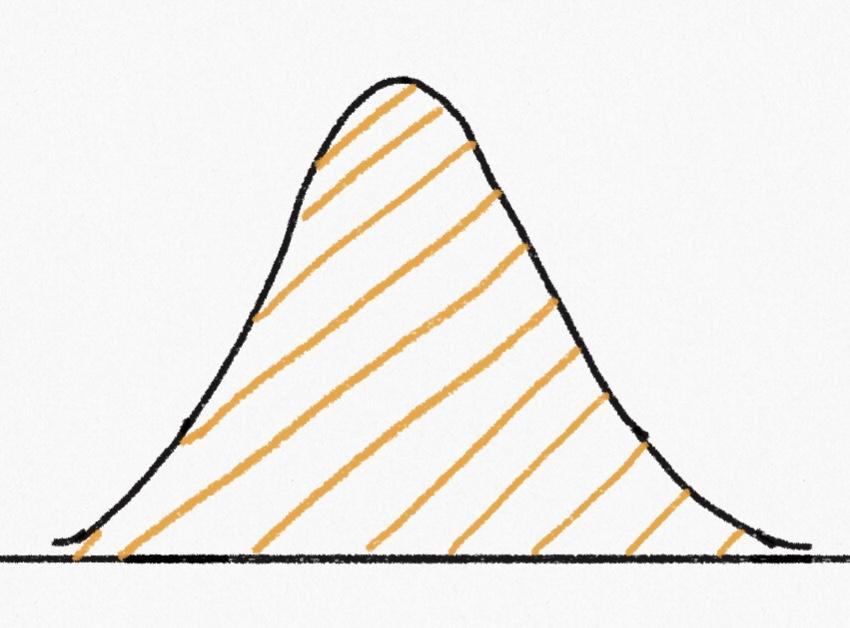


1. Which of the following are equal to 1? (Choose all that apply)
   1. The sum of the heights of all bars in a density histogram
   2. The sum of the areas of all bars in a density histogram
   3. The sum of the heights of all bars in a frequency histogram
   4. The sum of the areas of all bars in a frequency histogram
   5. The area under a normal probability curve
   6. The area under any probability curve
2. What does the area of a bar in a density histogram represent?
3. What will the probability distribution for a uniform histogram look like?
   1. a normal distribution
   2. a horizontal line
   3. a vertical line
   4. a random curve, impossible to determine the exact shape
4. If we randomly select a single data point from the distribution, what’s the probability of the point falling somewhere in the shaded region under the normal curve? Explain your answer.


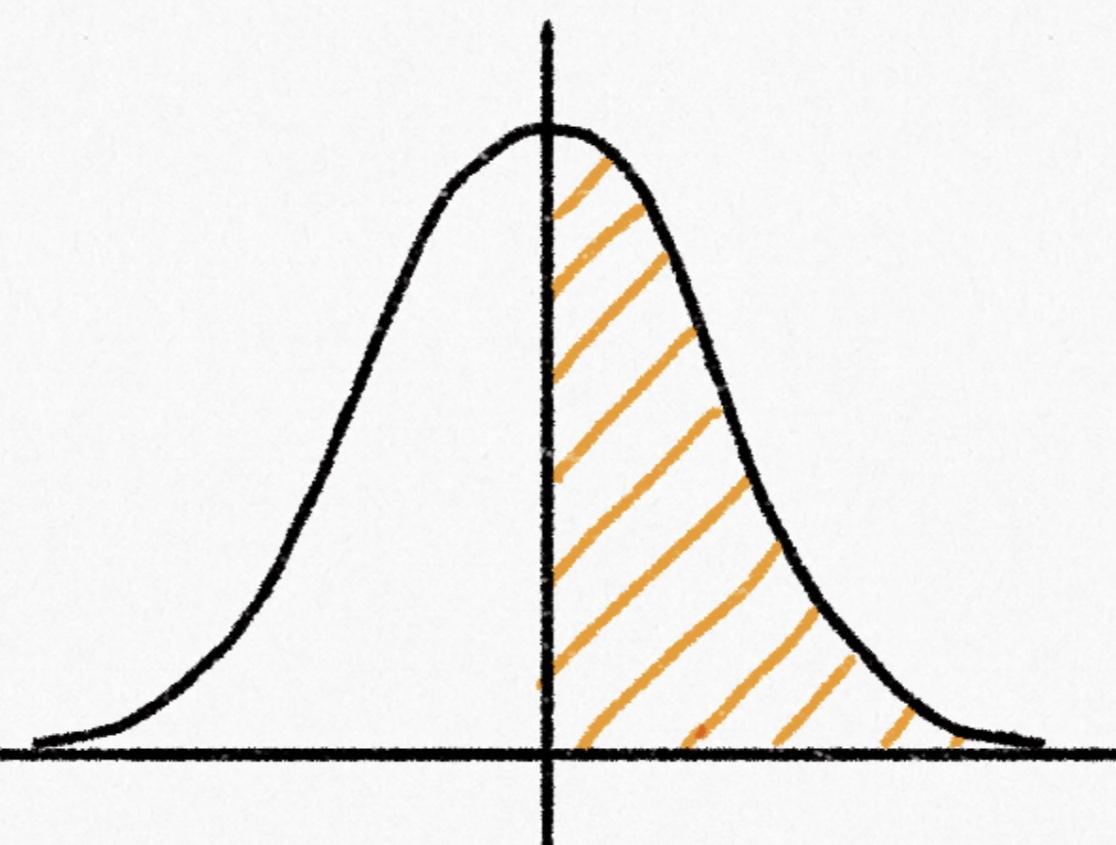


1. If we draw a normal distribution that is wider than the one in Question 1 (as below). How would the probability represented by the shaded part under the distribution change?


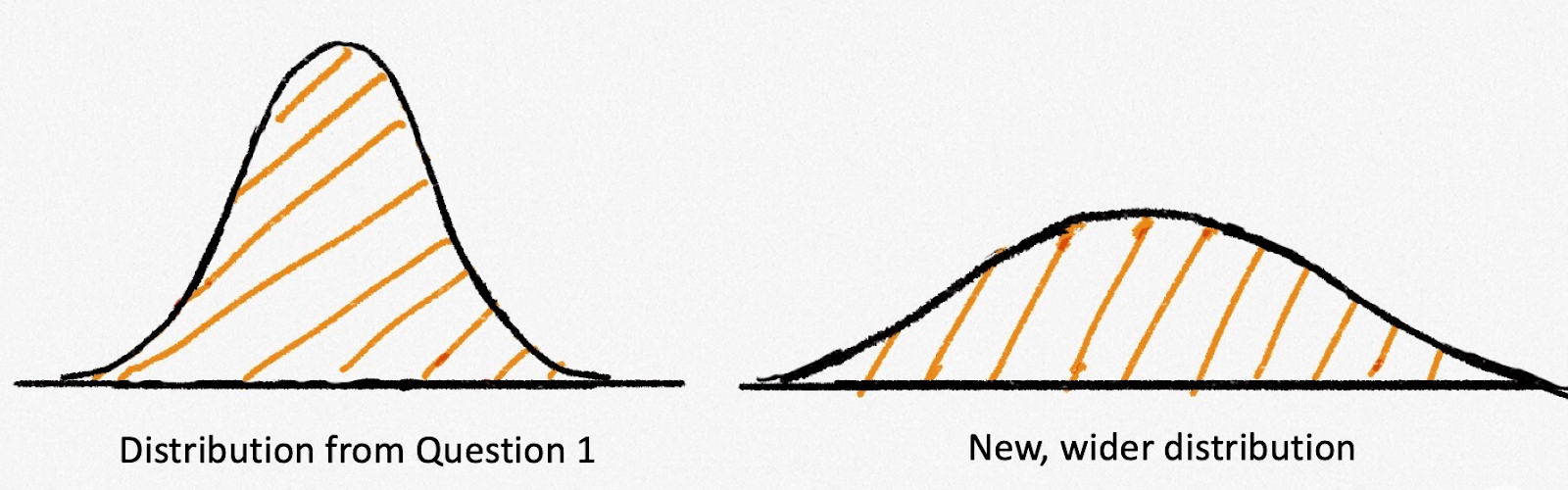


- 1. increase
  2. decrease
  3. stay the same
  4. impossible to tell

1. Explain your answer to the previous question:
2. Here’s a drawing of a normally shaped population distribution with a mean of 8. The probability of a randomly sampled data point being **greater than** 10 is 0.25. Based on this, what is the probability of a randomly sampled data point being **greater than** 6? Explain your answer.


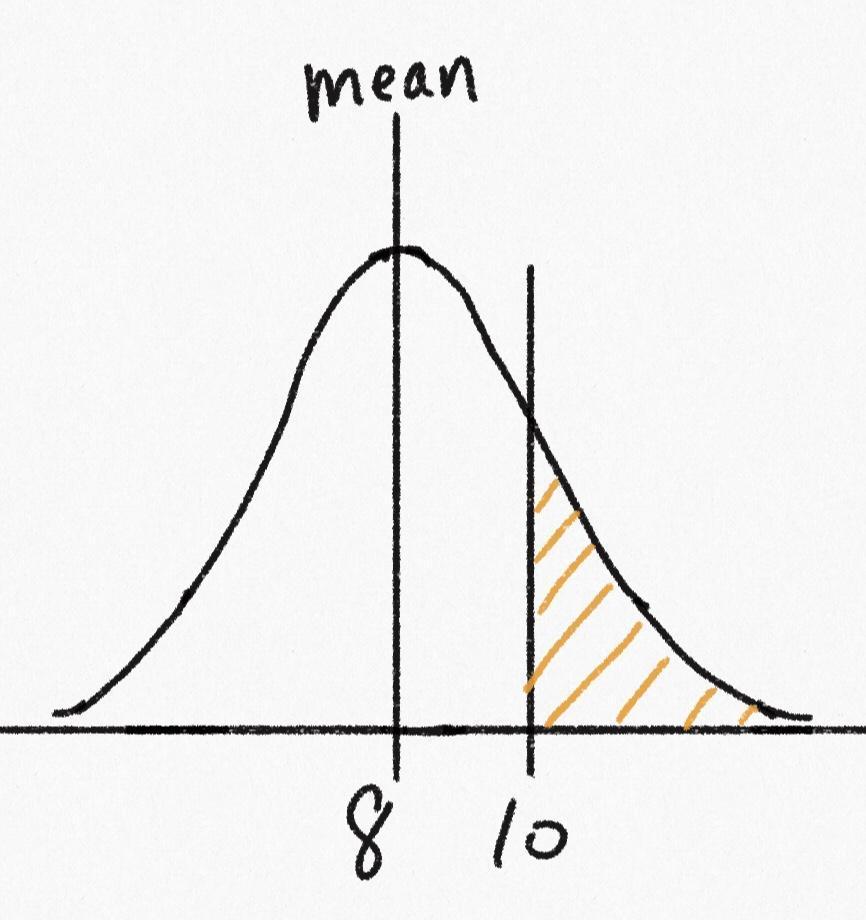


1. Let’s suppose we’ve got a distribution of a group of students’ scores on a test. The sample is normally distributed. The mean of the distribution is 125 and 20% of students scored lower than 100.

1) If the distribution were more variable (i.e., had a higher standard deviation), but still normal, with the mean being the same, what would happen to the probability that a randomly selected student from this distribution would score above the mean (125 or higher)? Explain your answer.

2) If the distribution were more variable (i.e., had a higher standard deviation), but still normal, with the mean being the same, what would happen to the probability that a randomly selected student from this distribution would score 100 or lower? Explain your answer.

3) If this time, the distribution were less variable (i.e., had a lower standard deviation), but still normal, with the mean being the same, what will happen to the probability that a randomly selected student from this distribution would score 100 or lower? Explain your answer.

1. Below is a normal probability distribution. Suppose 6 is a point on the graph like below, which of the following graph correctly represents the change of the graph (from top to bottom) when the distribution gets wider (more varied)?


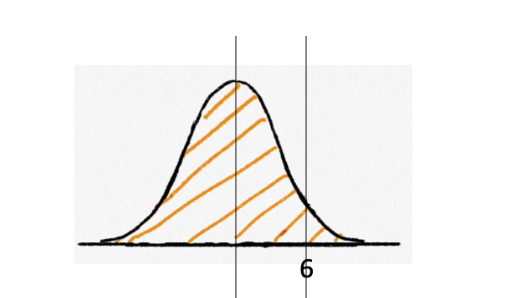


- 1.
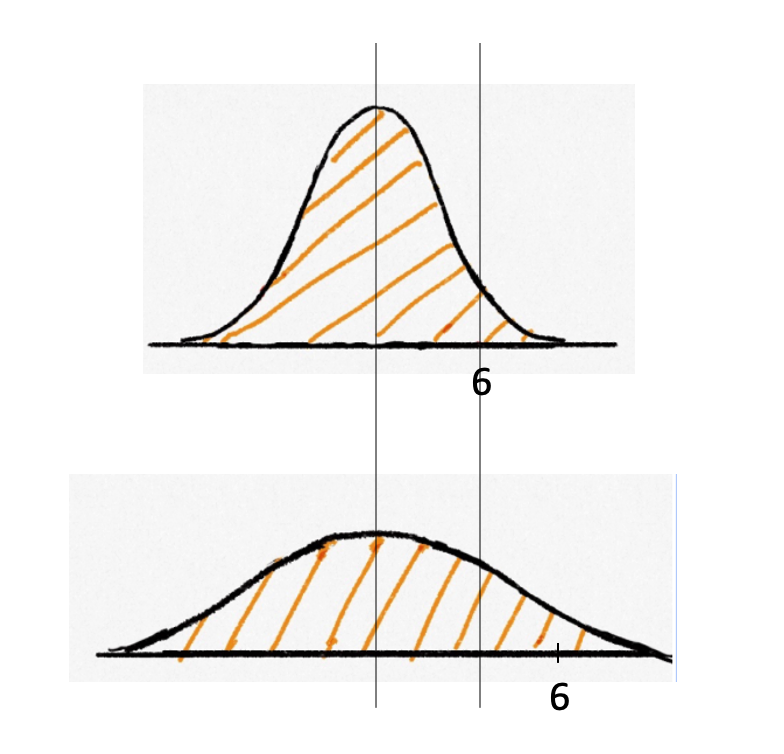

  2.
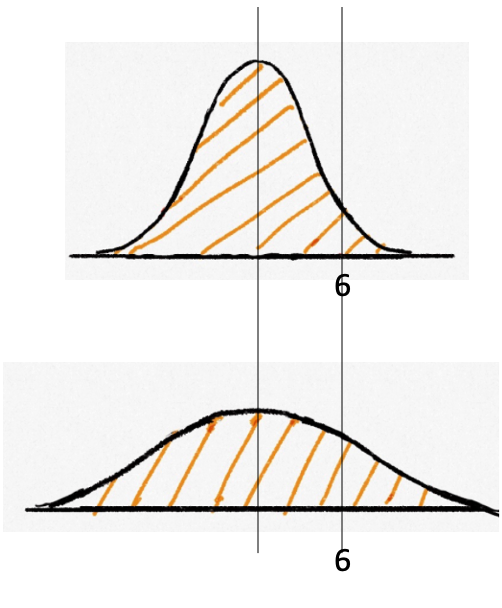

  3.
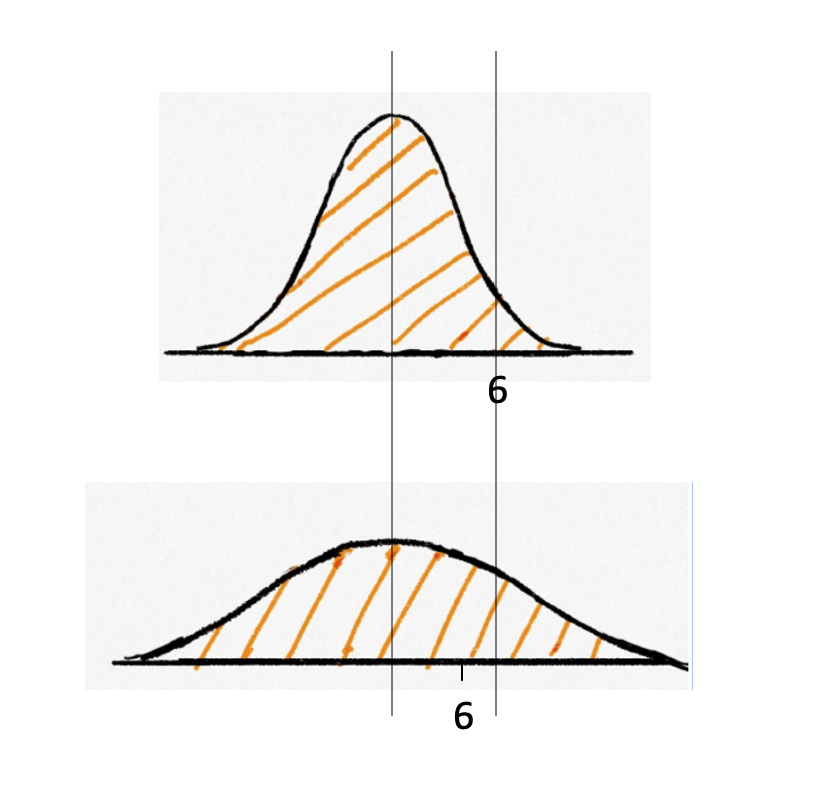


1. Based on your answer to the previous question, when the distribution gets wider,

1) how would the probability of getting a number higher than 6 change?

2) What about the probability of getting a number between the mean and 6?

1. We can use different models to model for a sample data. One of the most common models we use is the normal distribution. However, our sample distribution is not always normal in real life. In the graph below, the sample distribution is a bit skewed to the right. We modeled this sample distribution with a normal distribution. The mean of the sample distribution is 23.


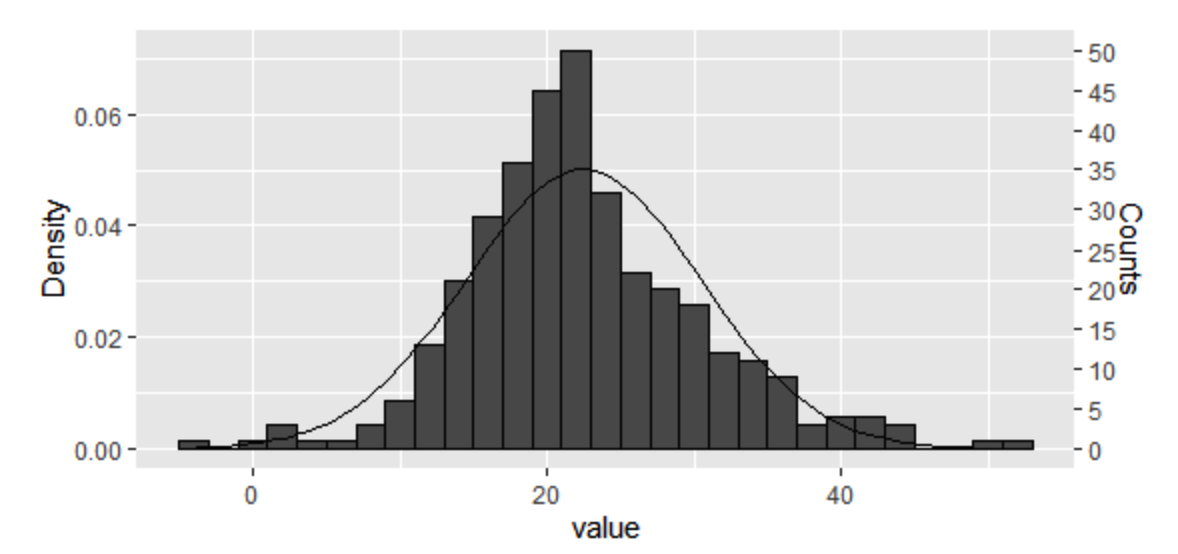


1) Under our normal distribution model, what’s the probability that a randomly selected data point will be larger than 23? Explain your answer.

2 )Does the prediction of our normal distribution model from the previous question hold true in our sample data? Explain your answer.

3) Do you think our normal model is a good model for this sample? Why or why not?

Appendix D: Study 2 Delayed Posttest

1. Which of the following are equal to 1? (Choose all that apply)
   1. The sum of the **heights** of all bars in a **density** histogram
   2. The sum of the **areas** of all bars in a **density** histogram
   3. The sum of the **heights** of all bars in a **frequency** histogram
   4. The sum of the **areas** of all bars in a **frequency** histogram
   5. The area under a **normal** probability curve
   6. The area under **any** probability curve
2. What does the area of a bar in a density histogram represent?
3. Below, there’s a normal distribution on the left. If, on the right, we drew a normal distribution that is wider than the one on the left. How would the probability represented by the shaded part under the distribution change? (Increase, decrease, stay the same or impossible to tell). Explain your answer.


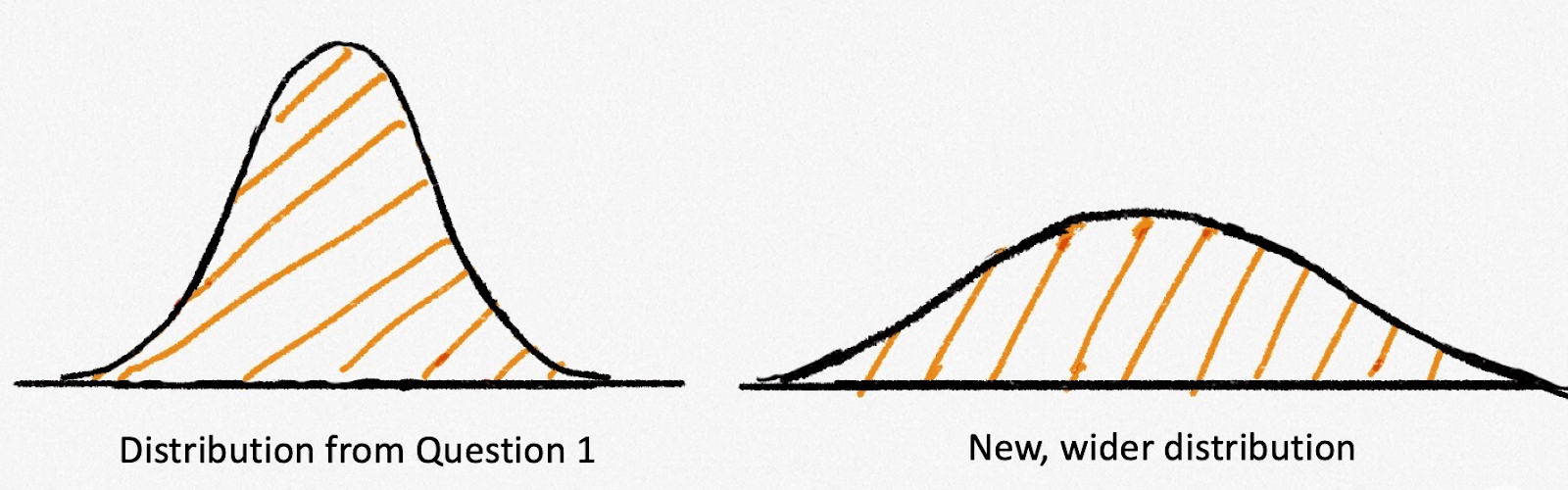


1. Suppose 6 is a point on the graph like below.


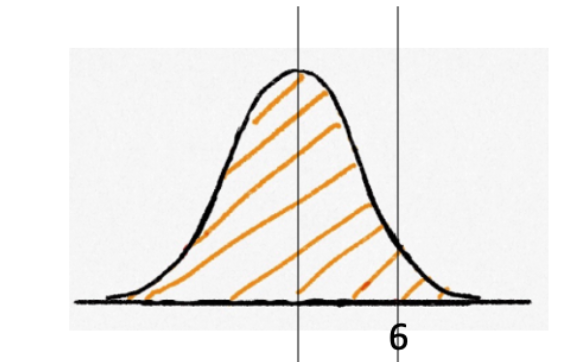


- 1. If the distribution becomes more variable, what would happen to the probability of getting a number higher than 6?

1. In a normal distribution of the birthweight of 100 newborns, the mean of the distribution is 7. If the distribution were **more variable** (i.e., had a higher standard deviation), but still normal, with the mean being the same, what would happen to the probability that a randomly selected newborn from this distribution would weigh 7 or higher?
   1. Increase
   2. Decrease
   3. Stay the same
   4. Impossible to tell
2. Explain your answer to the previous question.
3. Let’s suppose we’ve got a distribution of a group of students’ scores on a test. The sample is normally distributed. 20% of students scored lower than 100 in this distribution. If the distribution were **more variable** (i.e., had a higher standard deviation), but still normal, with the mean being the same, what would happen to the probability that a randomly selected student from this distribution would score 100 or lower?
   1. Increase
   2. Decrease
   3. Stay the same
   4. Impossible to tell
4. Explain your answer to the previous question.
5. Let’s suppose we’ve got here a normal distribution of participants’ performance on a clinical trial. The performance scores are normally distributed with a mean of 10. 10% of the participants scored higher than 15. If the distribution were **less variable** (i.e., had a lower standard deviation), but still normal, with the mean being the same, what will happen to the probability that a randomly selected student from this distribution will score between the mean(10) and 5?
   1. Increase
   2. Decrease
   3. Stay the same
   4. Impossible to tell
6. Explain your answer to the previous question.
7. Suppose there is a normal distribution with a mean of x. And y is a number in the distribution higher than x. If the distribution becomes less variable, but still normal, what would happen to the probability of getting a number higher than y ?
   1. Increase
   2. Decrease
   3. Stay the same
8. Explain your answer to the previous question
9. Suppose there’s a normal distribution with a mean of 8. The probability of a randomly sampled data point being **greater than** 10 in this normal distribution is 0.2. Suppose you take a random sample of 30 data points, and calculate the mean of these 30 data points. What would happen to the probability of getting **a sample that has a mean** greater than 10?
   1. Greater than 0.2
   2. Lower than 0.2
   3. Equal to 0.2
10. Explain your answer to the previous question.
11. Suppose there’s a sampling distribution of the means of newborns’ weight with a mean of 7. The probability of getting a number lower than 6 in this sampling distribution is 0.2. What would happen to the probability of a randomly selected baby from the population getting a number lower than 6?
    1. Greater than 0.2
    2. Lower than 0.2
    3. Equal to 0.2
12. Explain your answer to the previous question.
13. Which is more likely to be closer to the mean of the population?
    1. The average of a randomly selected sample of 30 data points
    2. A randomly selected data point
    3. Equally likely
    4. Impossible to tell

Appendix E: Study 3 Pretest

1. This is a normal probability distribution. Could you estimate the probability represented by the shaded region under the curve? If yes, what would your estimate be and why? If no, why not?


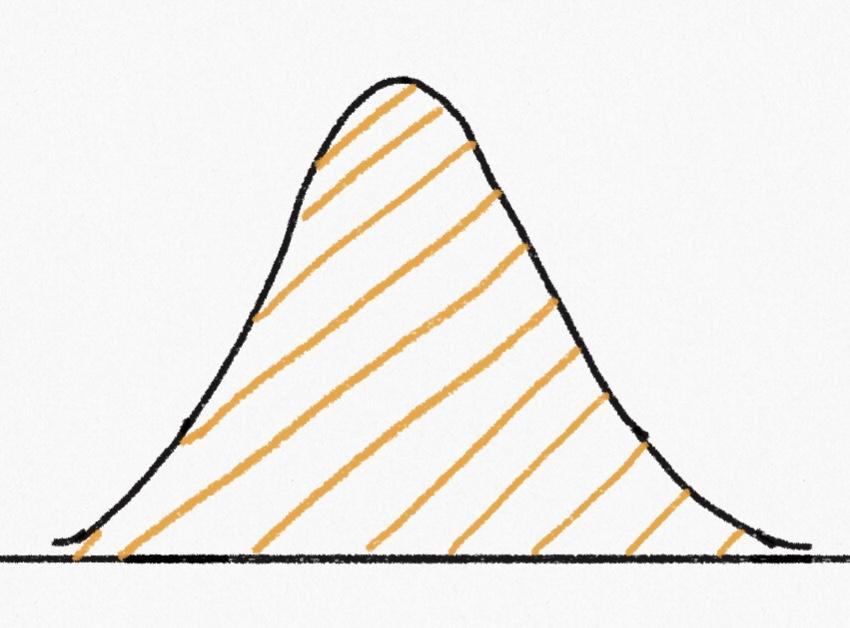


1. If we randomly select a single data point from the distribution, what’s the probability of the point falling somewhere in the shaded region under the normal curve? Explain your answer.


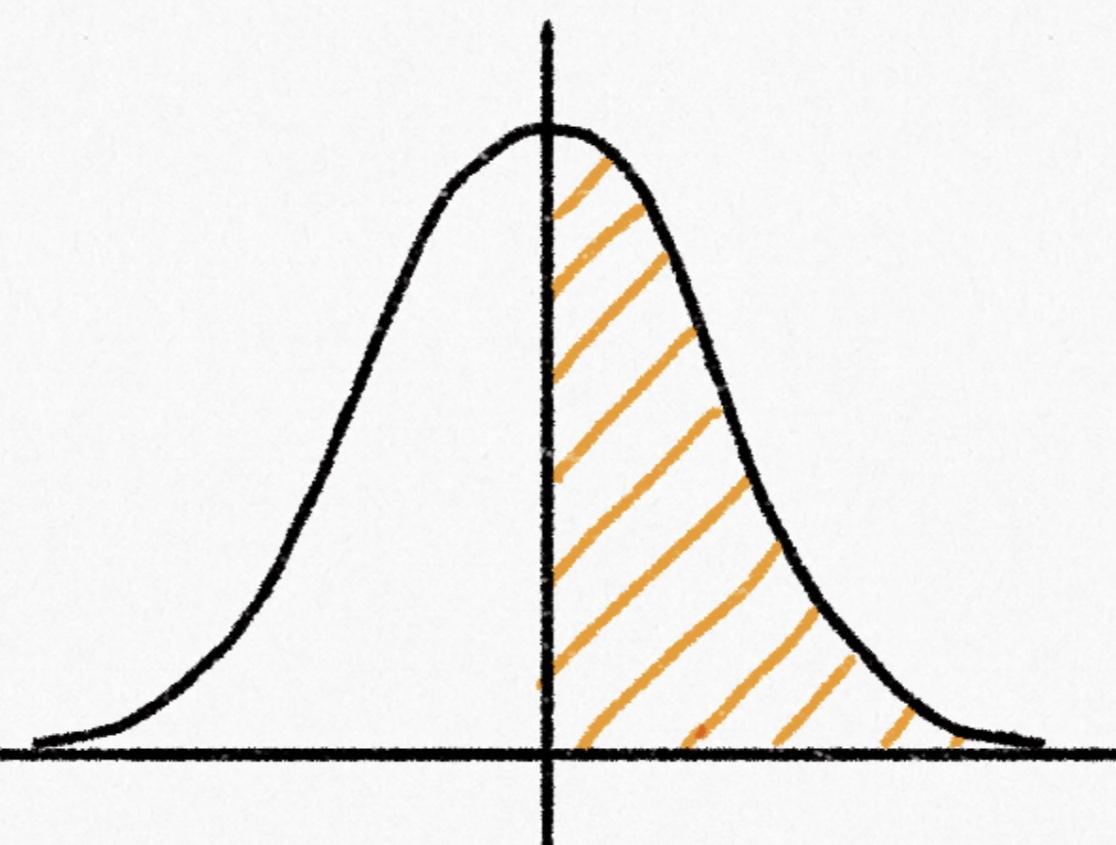


1. If we draw a normal distribution that is wider than the one in Question 1 (as below). How would the probability represented by the shaded part under the distribution change?


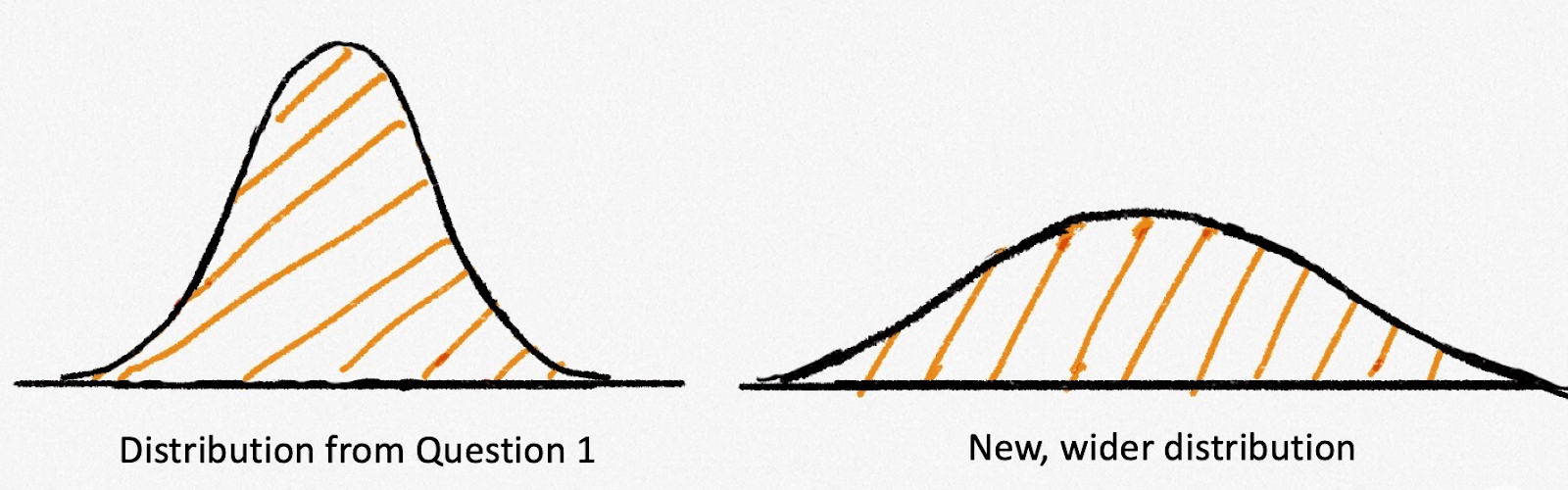


- 1. increase
  2. decrease
  3. stay the same
  4. impossible to tell

1. Explain your answer to the previous question:
2. Here’s a drawing of a normally shaped population distribution with a mean of 8. The probability of a randomly sampled data point being **greater than** 10 is 0.25. Based on this, what is the probability of a randomly sampled data point being **greater than** 6? Explain your answer.


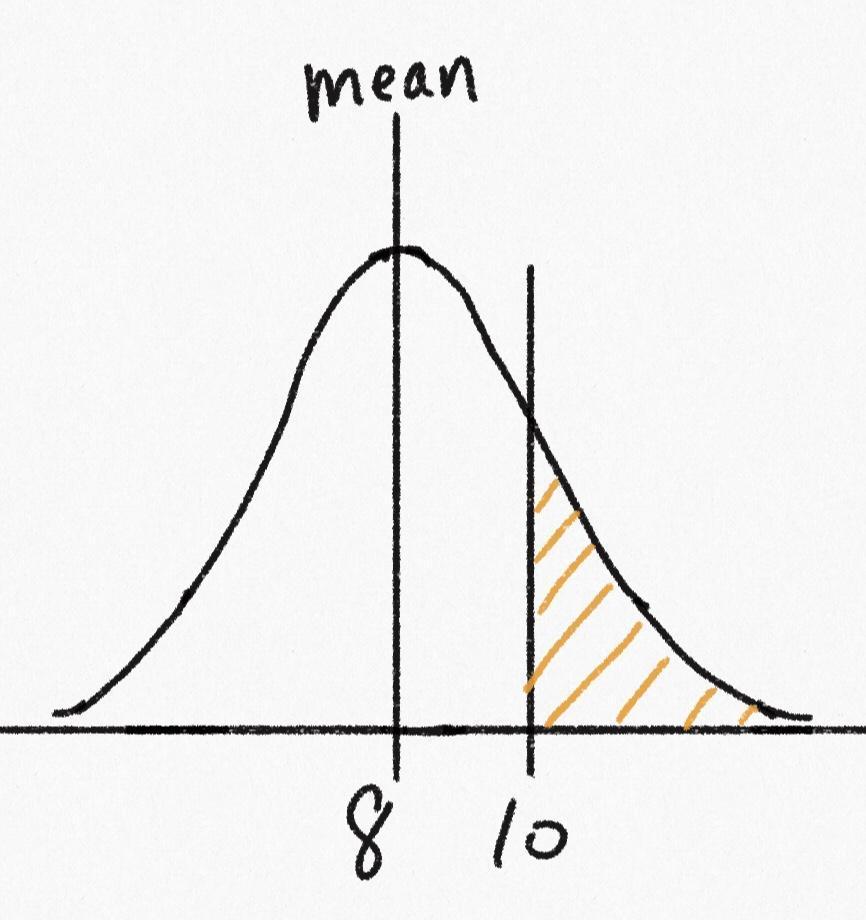


1. Based on what you know from the previous question, if you take a random sample of 30 data points, would the probability of getting a sample mean greater than 10 be greater than 0.25, lower than 0.25 or equal to 0.25? Explain your answer.

Appendix F: Study 3 Immediate Posttest

1. This is a normal probability distribution. Could you estimate the probability represented by the shaded region under the curve? If yes, what would your estimate be and why? If no, why not?


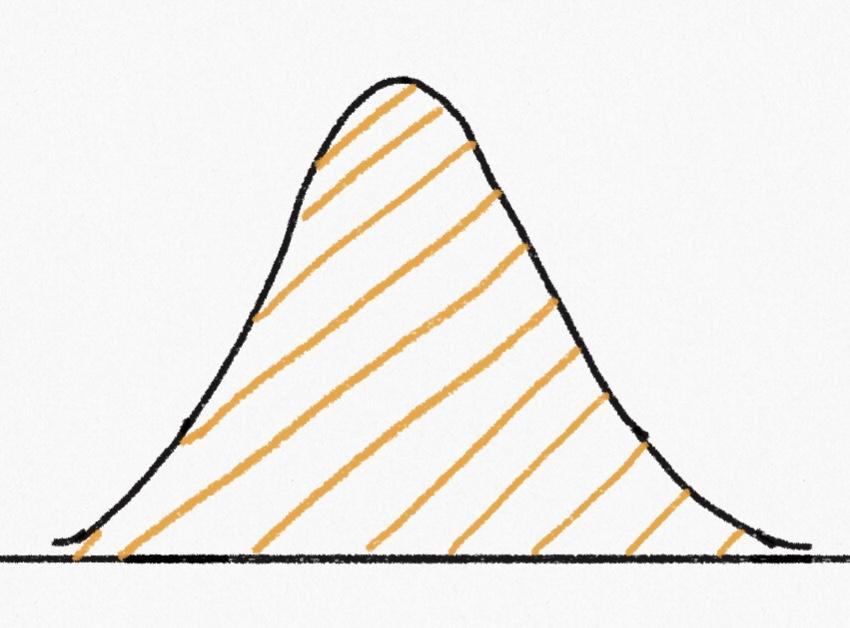


1. Which of the following are equal to 1? (Choose all that apply)
   1. The sum of the heights of all bars in a density histogram
   2. The sum of the areas of all bars in a density histogram
   3. The sum of the heights of all bars in a frequency histogram
   4. The sum of the areas of all bars in a frequency histogram
   5. The area under a normal probability curve
   6. The area under any probability curve
2. What does the area of a bar in a density histogram represent?
3. What will the probability distribution for a uniform distribution look like?
   1. a normal distribution
   2. a horizontal line
   3. a vertical line
   4. a random curve, impossible to determine the exact shape
4. If we randomly select a single data point from the distribution, what’s the probability of the point falling somewhere in the shaded region under the normal curve? Explain your answer.


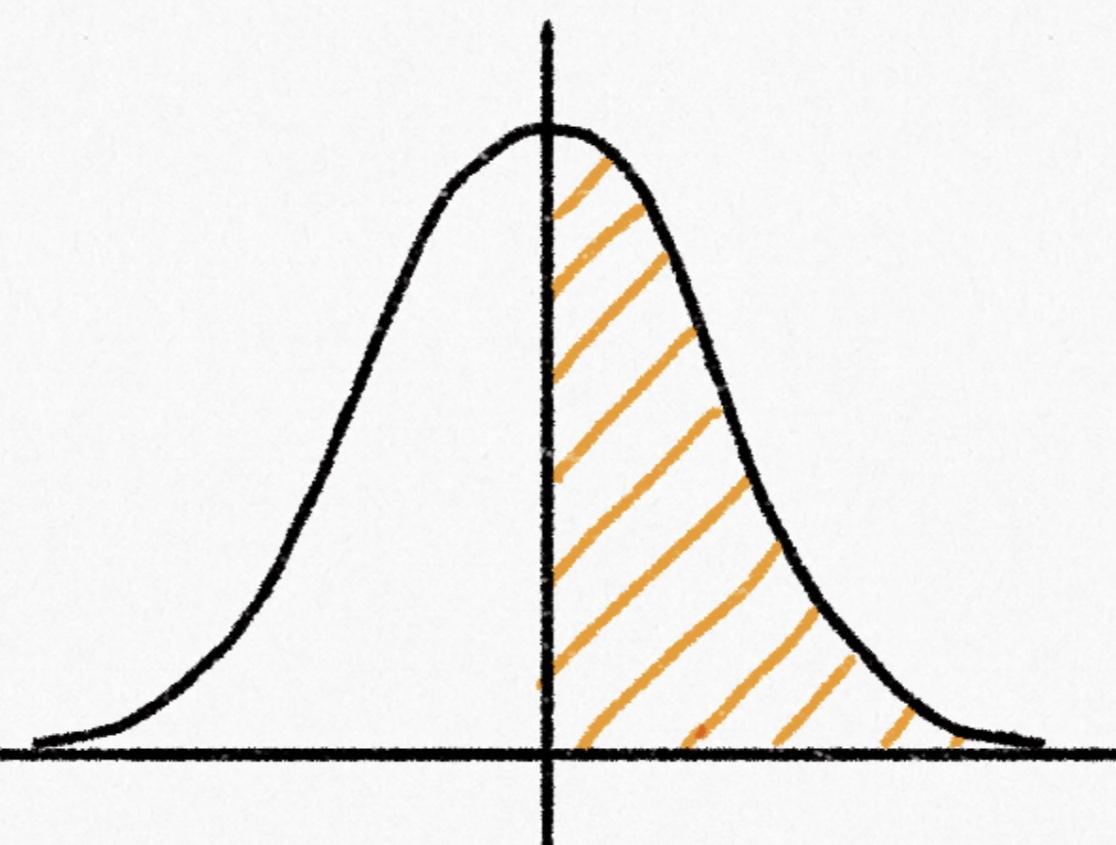


1. If we draw a normal distribution that is wider than the one in Question 1 (as below). How would the probability represented by the shaded part under the distribution change?


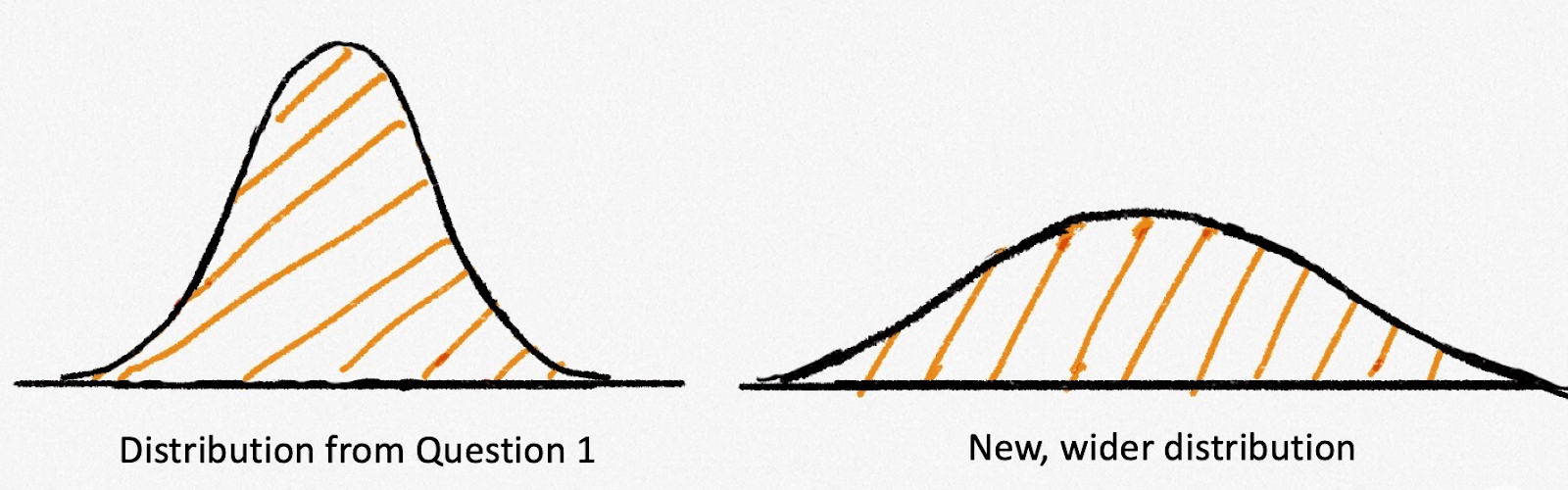


- 1. increase
  2. decrease
  3. stay the same
  4. impossible to tell

1. Explain your answer to the previous question:
2. Here’s a drawing of a normally shaped population distribution with a mean of 8. The probability of a randomly sampled data point being **greater than** 10 is 0.25. Based on this, what is the probability of a randomly sampled data point being **greater than** 6? Explain your answer.


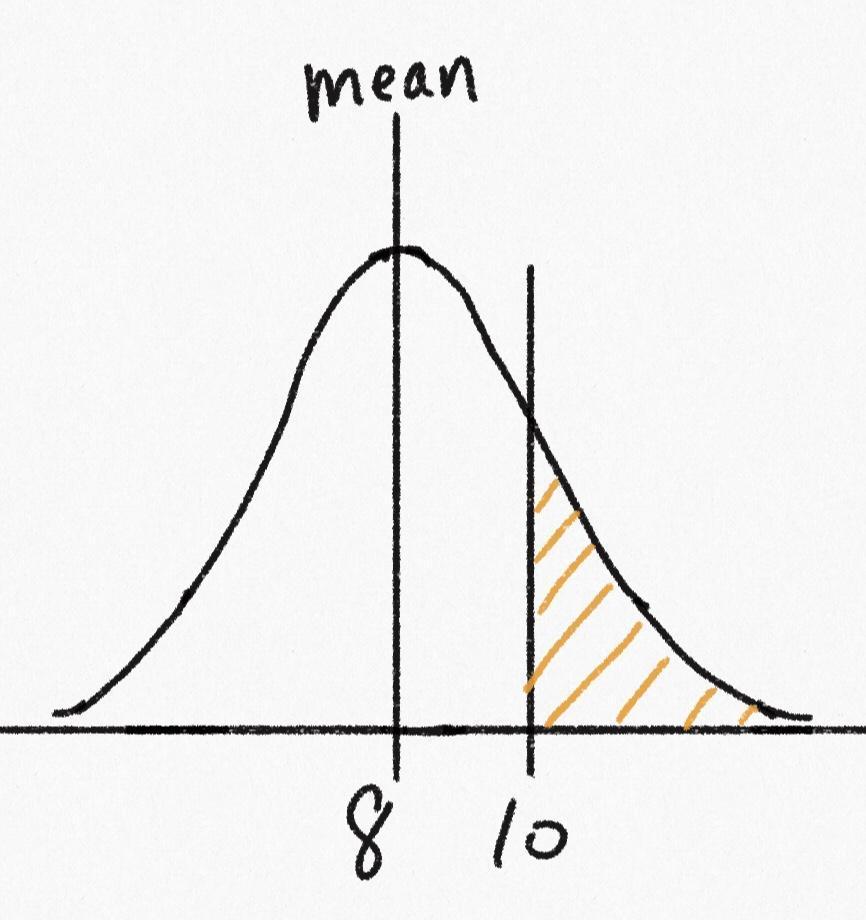


1. Let’s suppose we’ve got a distribution of a group of students’ scores on a test. The sample is normally distributed. The mean of the distribution is 125

If the distribution were more variable (i.e., had a higher standard deviation), but still normal, with the mean being the same, what would happen to the probability that a randomly selected student from this distribution would score above the mean (125 or higher)? Explain your answer.

1. Let's suppose we've got distribution of people's happiness rating. The sample is normally distributed. 30% of people rated their happiness to be lower than 50.

If the distribution were more variable (i.e., had a higher standard deviation), but still normal, with the mean being the same, what would happen to the probability that a randomly selected people from this distribution would have a happiness rating of 50 or lower? Explain your answer.

1. Below is a normal probability distribution. Suppose 6 is a point on the graph like below, which of the following graph correctly represents the change of the graph (from top to bottom) when the distribution gets wider (more varied)?


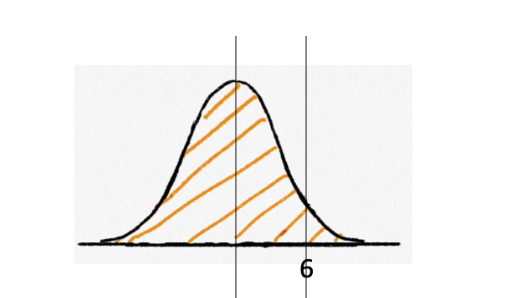


- 1.
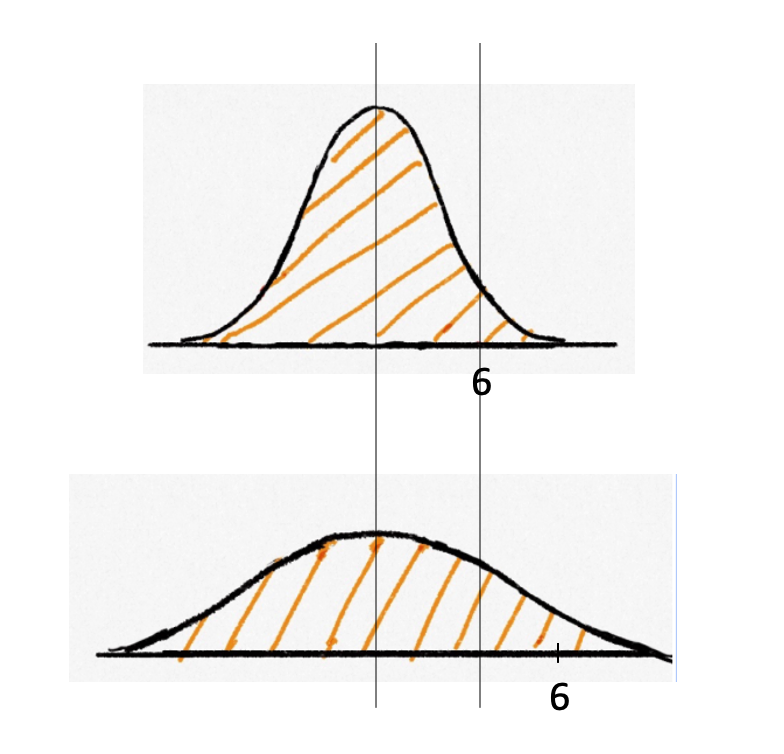

  2.
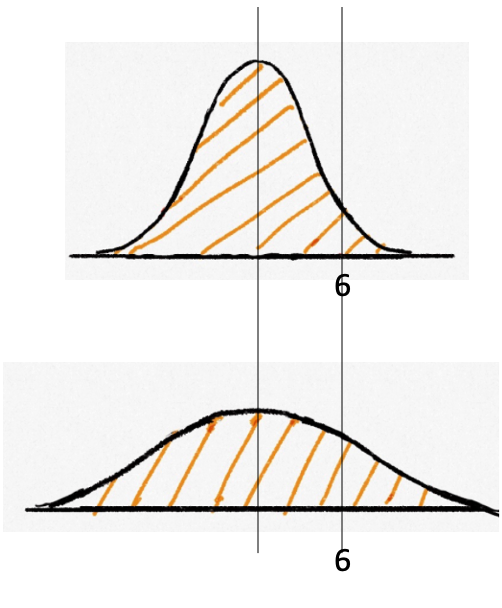

  3.
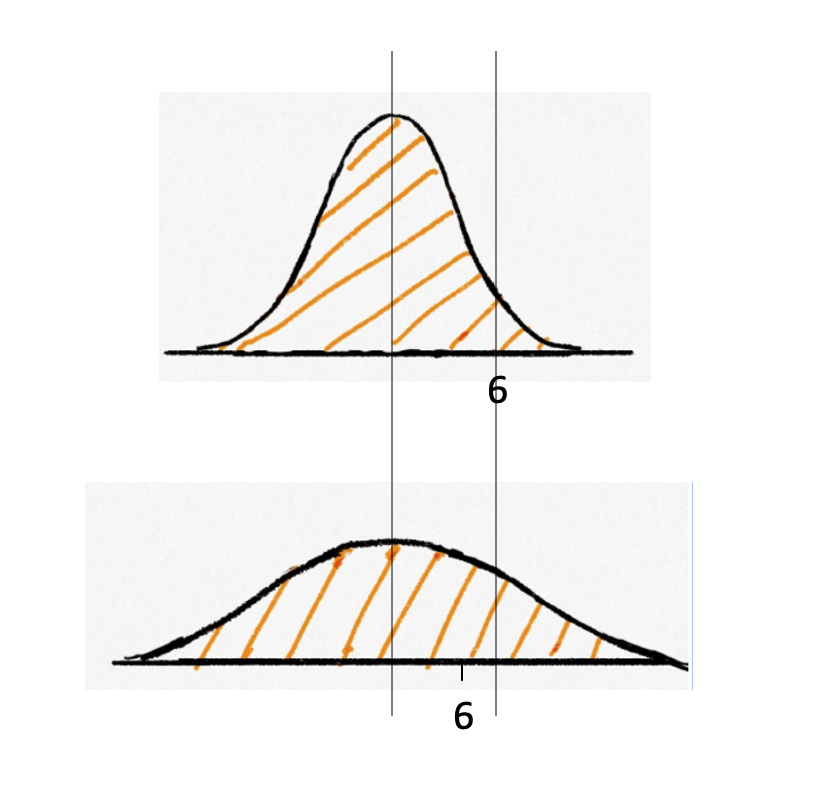


1. Based on your answer to the previous question, when the distribution gets wider, how would the probability of getting a number higher than 6 change?
2. We can use different models to model for a sample data. One of the most common models we use is the normal distribution. However, our sample distribution is not always normal in real life. In the graph below, the sample distribution is a bit skewed to the right. We modeled this sample distribution with a normal distribution. The mean of the sample distribution is 23.


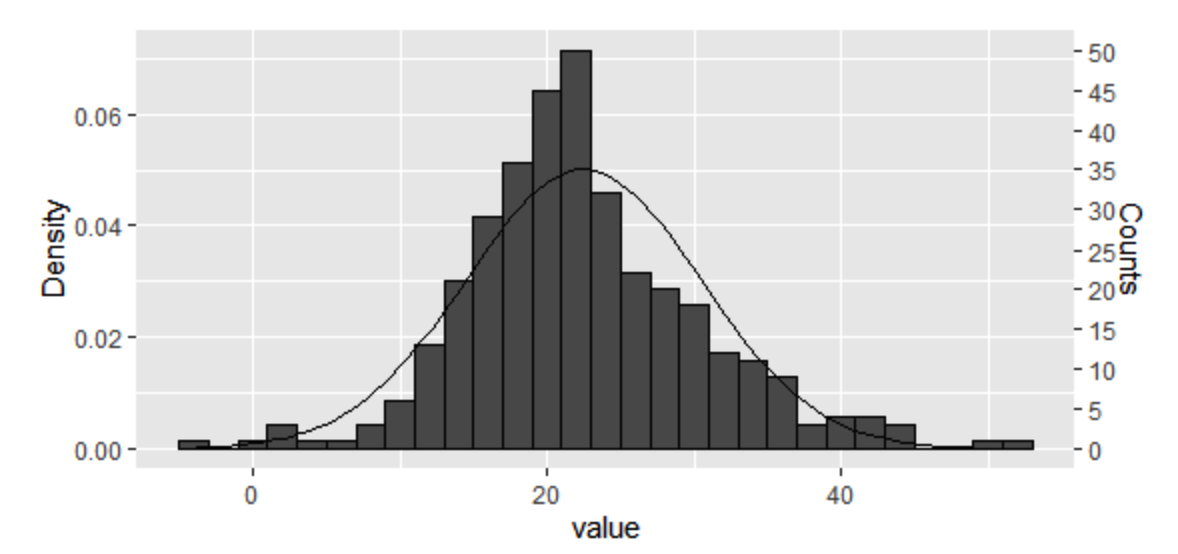


Under our normal distribution model, what’s the probability that a randomly selected data point will be larger than 23? Explain your answer.
